# ANTXR1 blockade enhances cardiac function in preclinical models of heart failure

**DOI:** 10.1038/s44161-025-00725-y

**Published:** 2025-10-02

**Authors:** Nicola Boccella, GuoJun Yu, Steven Seaman, Yang Feng, Jaewon Lee, Francesco Tomassoni-Ardori, Liping Yang, Kuo-Sheng Hsu, James M. Dunleavey, Jodi Becker, Mary Beth Hilton, Karen Morris, Niza Borchin, Daeho So, Pradip Bajgain, Sudhirkumar Yanpallewar, Ryan T. Gross, Krish C. Dewan, Dawn E. Bowles, Darren A. Yuen, Lino Tessarollo, Brad St. Croix

**Affiliations:** 1 Neural Development Section, Mouse Cancer Genetics Program (MCGP), National Cancer Institute (NCI), NIH, Frederick, MD USA; 2 Tumor Angiogenesis Unit, MCGP, NCI, NIH, Frederick, MD USA; 3 Basic Research Program, Leidos Biomedical Research Inc., Frederick National Laboratory for Cancer Research (FNLCR), Frederick, MD USA; 4 Department of Surgery, Duke University, Durham, NC USA; 5 Keenan Research Centre for Biomedical Science, Li Ka Shing Knowledge Institute, St. Michael’s Hospital, and Department of Medicine, University of Toronto, Toronto, Ontario Canada

**Keywords:** Myocardial infarction, Antibody therapy

## Abstract

Heart disease, a leading cause of mortality worldwide, is in urgent need of improved therapies. Fibrosis, an accumulation of collagen-rich extracellular matrix in response to injury, is a hallmark of heart disease, but clinical agents that can interfere with the fibrotic pathway do not yet exist. Here we show that ANTXR1/TEM8, a pathology-induced transmembrane protein required for collagen removal, exacerbates injury in multiple models of heart failure. Genetic disruption of *Antxr1* and treatment with human neutralizing antibodies prevented heart deterioration following acute myocardial infarction. ANTXR1 pharmacological blockade also improved heart function in models of pressure overload and obesity-induced heart disease with preserved ejection fraction. Improved heart function was accompanied by enhanced exercise tolerance. Mechanistic studies revealed an ANTXR1-antibody-driven improvement in post-infarct scar formation followed by attenuation of late-stage, chronic TGFβ-mediated extracellular matrix remodeling. Thus, ANTXR1-mediated collagen turnover during heart failure is both maladaptive and druggable, providing avenues for therapeutic intervention.

## Abstract (web summary)

Boccella, Yu and colleagues reveal that the transmembrane protein ANTXR1 regulates post-infarction fibrotic remodeling, and its inhibition blocks collagen turnover and improves heart function.

## Main

Ischemic heart disease is the leading global cause of mortality, accounting for ~9 million deaths annually^1,2^. Unfortunately, many survivors of myocardial infarction (MI) experience progressive cardiac decline afterwards, with mortality approaching 20% within the first year. Acute MI, usually caused by coronary artery occlusion, induces cardiomyocyte apoptosis followed by cardiac fibroblast (CF) activation and scar formation. Because cardiomyocytes regenerate poorly, prompt fibrotic scar development is critical for preventing rupture and stabilizing the ventricular wall. Hypertension, affecting over 1.2 billion adults worldwide, is a major contributor to cardiovascular disease, including heart failure (HF) with reduced ejection fraction (HFrEF) and preserved ejection fraction (HFpEF). Neurohormonal antagonists improve outcomes in HFrEF, but HFpEF (representing roughly half of HF hospitalizations) has remained refractory to effective therapy^3^. Thus, new approaches to limit progressive HF are urgently needed.

Fibrosis, defined by excess collagen-rich extracellular matrix (ECM) deposition, is a hallmark of all major forms of heart disease, including MI, hypertrophic cardiomyopathy (HCM), dilated cardiomyopathy (DCM) and HFpEF^4,5^. This fibrotic response is triggered by diverse insults (ischemia, hypertension, diabetes, obesity, genetic causes or a combination of these and other factors). Transforming growth factor (TGF)β converts CFs into collagen-secreting myofibroblasts, driving maladaptive remodeling, yet systemic TGFβ inhibition remains challenging, primarily due to its pleiotropic interactions with other nonfibroblast cell types^6,7^. ANTXR1 (anthrax toxin receptor 1), also known as tumor endothelial marker 8 (TEM8), is a conserved integrin-like collagen receptor previously implicated in fibrosis^8,9^. Initially identified in the tumor vasculature, ANTXR1 is also enriched in cancer-associated fibroblasts (CAFs)^9–12^. In tumors, nutrients stress and ischemia induce ANTXR1, which promotes angiogenesis and tumor growth^9,13^. While expression in normal tissues is low, it is inducible in fibroblasts and endothelial cells under stress^9,13–15^. Global ANTXR1 disruption protects against cancer and causes progressive collagen accumulation in multiple organs^8,9,15,16^, suggesting a role in regulating collagen deposition.

Despite these insights, the function of ANTXR1 in cardiovascular disease is unknown. Given its regulation by ischemia and involvement in collagen uptake^9,14^, we postulated that ANTXR1 may minimize heart disease by restraining excessive fibrosis after MI. Contrary to our initial hypothesis, studies in *Antxr1* knockout (KO) mice revealed the opposite; post-MI ANTXR1 activity was predominantly deleterious. After discovering it was harmful, we then investigated the impact of antibody-mediated ANTXR1 blockade and used single-cell transcriptomics, conditional CF-specific deletion and biochemical and histological analyses to define its mechanism. These experiments showed that injury-induced ANTXR1 amplifies maladaptive canonical TGFβ signaling in CFs, ultimately driving HF.

## Results

### ANTXR1 promotes HF after MI

**Fig. 1:**
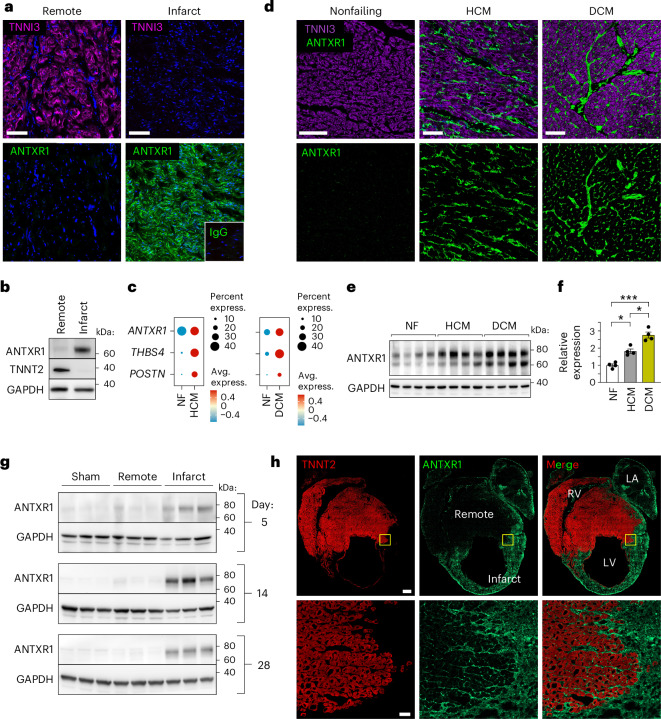
Expression of ANTXR1 in heart post-injury. **a**, IF staining of ANTXR1 (green) and TNNI3 (cardiac troponin; magenta) in human LV following MI. The ANTXR1-positive image was taken from a ‘focal hotspot’ in the infarct zone. The inset shows the nonbinding IgG control captured at the same magnification. Image is representative of three independent samples. Scale bar, 100 µm. **b**, Immunoblotting of remote or infarct region from LV of a failing human heart after MI. TNNT2; cardiac troponin T. **c**, scRNA-seq dotplots comparing *ANTXR1* levels in CFs from hypertrophic cardiomyopathy (HCM), dilated cardiomyopathy (DCM) and nonfailing (NF) hearts. *THBS4* and *POSTN*, markers of activated fibroblasts, served as positive controls. **d**, IF staining of ANTXR1 (green) and TNNI3 (violet) in LV specimens from patients with NF hearts, HCM or DCM. Image is representative of four independent samples per group. Scale bar, 100 µm. **e**,**f**, Immunoblots detecting ANTXR1 protein in human NF, HCM and DCM LV specimens (**e**) and quantification relative to NF heart samples (**f**). *n* = 4 biological specimens per group. **g**, Immunoblotting for ANTXR1 in mouse sham controls and MI hearts at different time points post-LAD ligation. Three specimens per group are shown and are representative of at least six per group. GAPDH served as a loading control in **b**, **e** and **g**. **h**, IF staining for ANTXR1 (green) and TNNT2 (red) in mouse heart 7 days post-MI. Image is representative of at least ten specimens. RV, right ventricle. Scale bar, 500 µm (top) or 50 µm (bottom). Data are shown as mean ± s.e.m. The statistical analysis in **f** was performed using a one-way analysis of variance (ANOVA) with Tukey’s multiple-comparisons test. *P < 0.05, ***P < 0.001. Source data

**Extended Data Fig. 1:**
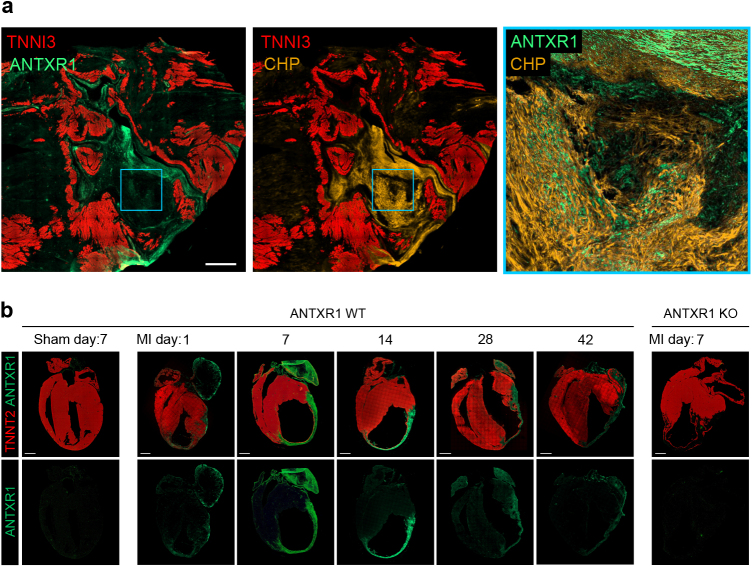
Localization of ANTXR1 post-myocardial infarction. **a**) Immunofluorescence staining was performed on border zone (region between scar and remote area) of a left ventricle (LV) sample taken from the failing heart of a 55-year-old transplant patient ~2.5 months following myocardial infarction. Image is representative of 3 independent samples. Note that ANTXR1 (green) was generally enriched near regions with high levels of denatured collagen, as detected with a collagen hybridizing peptide (CHP, orange). Anti-TNNI3 (cardiac troponin) antibodies were used to label cardiomyocytes (red). Bar: 2 mm. **b**) Localization of ANTXR1 in mouse hearts was examined by performing immunofluorescence staining for ANTXR1 (green) and TNNT2 (red) on hearts taken at various time points following myocardial infarction (MI). Images are representative of at least 3 independent samples per time point. Sham controls (surgery without MI) and MI hearts taken from *Antxr1* KO mice were both taken at d7 post-surgery and used to demonstrate specificity. Bar: 1 mm.

To assess ANTXR1 in ischemic heart disease, we first examined protein expression in hearts from post-MI transplant recipients. Co-immunofluorescence (IF) staining showed low ANTXR1 in cardiomyocyte-rich [troponin1 (TNNI3)-positive] non-infarct regions but high expression in TNNI3-negative scar ‘hotspots’ (Fig. 1a and Extended Data Fig. 1a). At 2 months after MI, focal ANTXR1 staining was most prominent at sites of active collagen remodeling, as indicated by colocalization with denatured collagen detected by a collagen hybridizing peptide (CHP) (Extended Data Fig. 1a). Immunoblotting confirmed ANTXR1 overexpression in infarct regions, inversely correlating with troponin (Fig. 1b). Public single-cell RNA sequencing (scRNA-seq) datasets^17,18^ also revealed elevated *ANTXR1* messenger RNA in CFs from patients with HCM and DCM (Fig. 1c). Consistently, IF and immunoblotting showed higher ANTXR1 in HCM and DCM compared to nonfailing hearts, localized to interstitial stromal cells (Fig. 1d–f and Supplementary Table 1). Together, these data demonstrate widespread induction of ANTXR1 in human heart disease.

We next analyzed ANTXR1 in a mouse model of coronary artery occlusion wherein MI is evoked by coronary artery ligation. ANTXR1 expression in infarct lysates peaked 5–14 days post-MI, but remained low in distal regions and left ventricle (LV) from non-infarcted sham controls (Fig. 1g). IF confirmed localized ANTXR1 expression in the scar, peaking between days 7–14 and declining by day 42 post-MI (Fig. 1h and Extended Data Fig. 1b) and absent in shams and *Antxr1* KO MI hearts (Extended Data Fig. 1b).

**Fig. 2:**
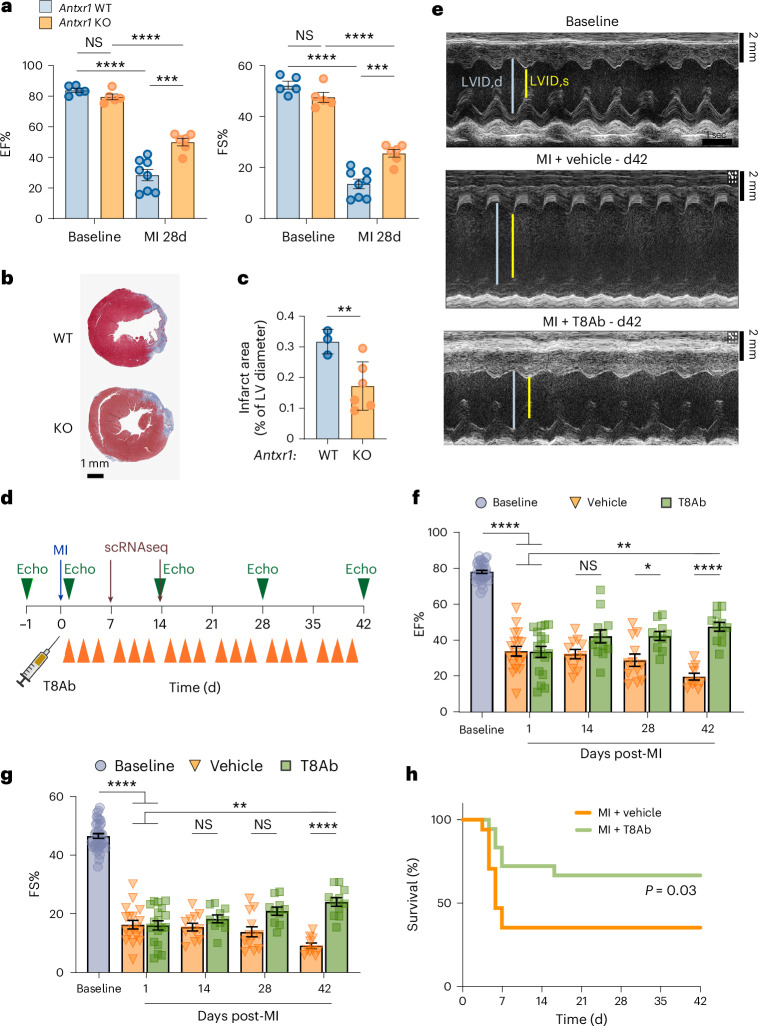
Genetic and pharmacological antagonism of ANTXR1 improves outcomes post-MI. **a**, Echocardiography showing left ventricular ejection fraction (EF%) and fractional shortening (FS%) 28 days post-MI in *Antxr1* WT and KO mice. *n* = 5 per group (baseline), or 6 (KO) or 8 (WT) per group (day 28). **b**, Representative Masson’s trichrome stain of *Antxr1* WT and KO heart 28 days post-MI. **c**, Quantification of infarct area from hearts stained as shown in **b**. *n* = 3 (WT) or 6 (KO) per group. **d**, Experimental design for MI model with T8Ab treatment. Vehicle (control) or T8Ab treatments (orange arrowheads) began 1 day post-MI. **e**, Representative echocardiography tracings at baseline and 42 days post-MI with and without T8Ab. **f**,**g**, EF% (**f**) and FS% (**g**) at baseline and day 1 or 42 post-MI. *n* = 18 per group. **h**, Kaplan–Meier survival analysis post-MI in mice treated with vehicle or T8Ab. *P* value was from a log-rank (Mantel–Cox) test. *n* = 17 (vehicle) or 18 (T8Ab). Data are shown as mean ± s.e.m. (**a**,**f**,**g**) or mean ± s.d. (**c**); statistical analysis was performed using one-way ANOVA with Tukey’s multiple-comparisons test (**a**,**f**,**g**) or Welch’s two-tailed *t*-test (**c**). NS, not significant. *P < 0.05, **P < 0.01, ***P < 0.001, ****P < 0.0001. The syringe in **d** created with BioRender.com. Source data

To test function, MI was induced in *Antxr1* wild-type (WT) and KO mice. While survival was similar (Supplementary Fig. 1), KO mice exhibited improved cardiac function (higher fractional shortening (FS) and ejection fraction (EF)) and reduced scar size on day 28 post-MI (Fig. 2a–c and Supplementary Table 2). These findings prompted evaluation of therapeutic blockade. For this, we used the L2 fully cross-species (human/mouse) reactive anti-ANTXR1 antibody (herein called T8Ab), previously developed for cancer therapy, which showed no toxicity after 6 weeks of dosing (20 mg kg^−1^, 3× per week)^13^. T8Ab is a human/mouse reverse chimera that combines a human variable domain with a murine constant domain, enabling long-term dosing in immunocompetent mice. Here, male C57BL6 mice received intraperitoneal T8Ab (15 mg kg^−1^, 3× per week) for 6 weeks, starting 24 h post-MI (Fig. 2d). A 24-h post-MI delay in antibody administration was intentionally implemented to model a therapeutic regimen with potential clinical translatability. Compared to vehicle controls where heart function progressively declined, T8Ab treatment significantly improved cardiac function during the same period (Fig. 2e–g and Supplementary Table 3) and improved survival (67% versus 35%, Fig. 2h). In female mice, post-MI dysfunction was less severe, consistent with previous reports^19,20^, but T8Ab still significantly improved EF and other parameters (Supplementary Fig. 2 and Supplementary Table 4).

### ANTXR1 drives hypertensive HF

**Fig. 3:**
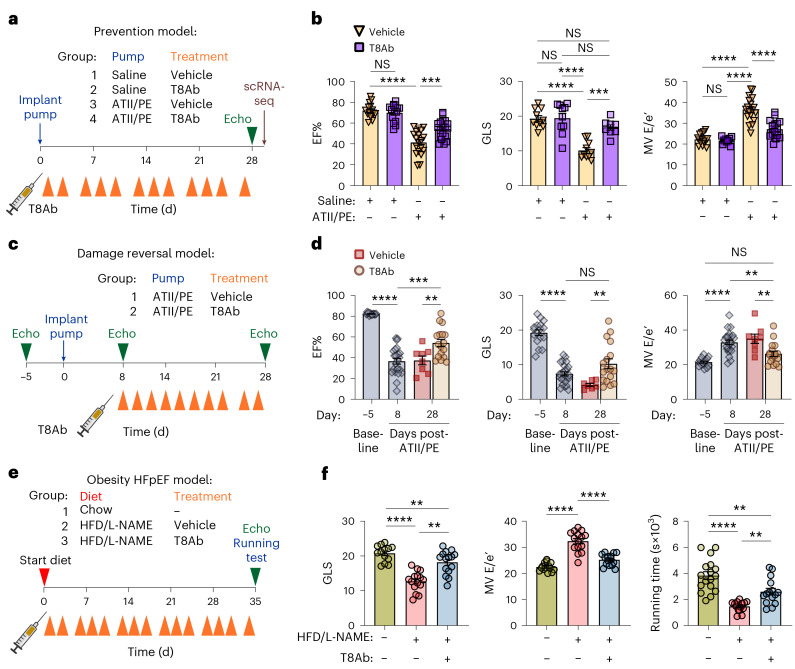
T8Ab improves cardiac performance in models of pressure overload and HFpEF. **a**, Experimental design for ATII/PE prevention model. Vehicle or T8Ab treatments (15 mg kg^−1^ 3× per week, orange arrowheads) began 1 day post-ATII. **b**, EF%, GLS and ratio of early diastolic mitral inflow velocity to early diastolic mitral annulus velocity (MV E/e′) on day 28. *n* = 15 mice per group (saline + vehicle and saline + T8Ab) or 19 mice per group (ATII/PE + vehicle and ATII/PE + T8Ab). **c**, Experimental design for ATII/PE damage reversal model. Vehicle or T8Ab treatments (15 mg kg^−1^ 3× per week, orange arrowheads) began 8 days post-ATII. **d**, EF%, GLS and MV E/e′ ratios for the study outlined in **c**. *n* = 8 (vehicle) or 17 (T8Ab) mice per group. **e**, Experimental design for HFD/L-NAME model of HFpEF. Vehicle or T8Ab treatments (15 mg kg^−1^ 3× per week, orange arrowheads) began 1 day post-ATII. **f**, Cardiac function assessment and endurance test results at 35 days post-HFD/L-NAME. *n* = 15 per group. Results represent mean ± s.e.m. An ordinary one-way ANOVA with Tukey’s post hoc test was used for multiple comparisons (**b**,**d**,**f**). *P < 0.05, **P < 0.01, ***P < 0.001, ****P < 0.0001. The syringe in **a**, **c** and **e** was created in BioRender. Source data

Although undetectable in sham hearts, ANTXR1 was expressed after MI in both the LV scar and non-ischemic left atrium (LA) (Fig. 1h and Extended Data Fig. 1b). LA fibrosis in injured hearts was previously attributed to pressure overload^21^. Together with elevated expression in HCM/DCM (Fig. 1c–f), these findings suggest that ANTXR1 expression mirror fibrosis driven by both ischemia and hypertension. To test its role in pressure overload, we used an angiotensin II (ATII)/phenylephrine (PE)-induced hypertension model (Fig. 3a). Starting 1 day after ATII/PE, mice received vehicle or T8Ab antibody 3× per week for 4 weeks. Echocardiography showed significantly improved systolic and diastolic function in T8Ab-treated mice, accompanied by reduced fibrosis (Fig. 3b, Supplementary Fig. 3 and Supplementary Table 5), indicating that ANTXR1 blockade also protects against hypertensive injury.

Improved function could reflect variable domain (VD)-mediated blockade or Fc-mediated recruitment of FcγR^+^ cells. To test this, we generated an Fc-inactive mutant (T8Ab-FI) with three heavy-chain amino acid substitutions (L234A, L235A and P329G) that disrupt binding to FcγR^22,23^. T8Ab-FI protected against hypertensive injury (Supplementary Fig. 4 and Supplementary Table 6), demonstrating that functional blockade of ANTXR1, not Fc interactions, drives cardioprotection. Together with the improved phenotype of *Antxr1* KO versus WT mice, these data confirm that ANTXR1 inhibition underlies T8Ab efficacy.

**Extended Data Fig. 2:**
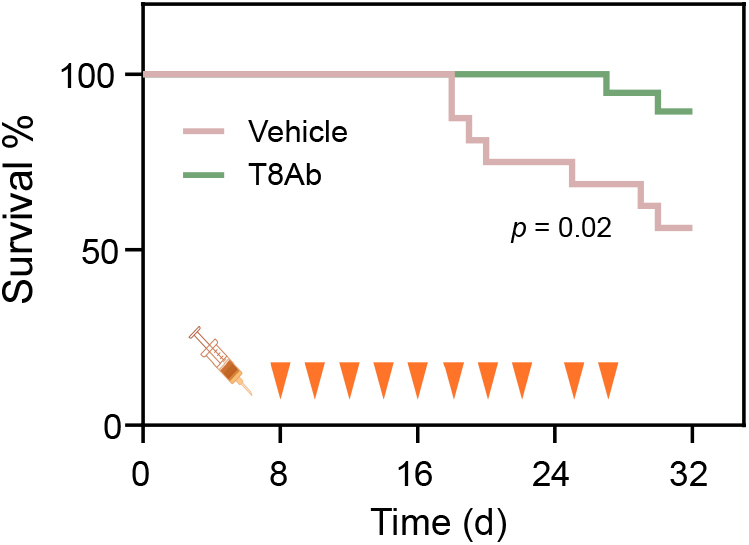
T8Ab increased survival following pressure overload. Kaplan–Meier survival analysis in hypertensive mice treated with vehicle or T8Ab. The experimental design for the ATII/PE study is outlined in Fig. 3c of the main text. Vehicle or T8Ab treatments (15 mg/kg 3x/week, orange arrowheads) began 8 days post-ATII. P value was from a log-rank (Mantel–Cox) test. n=16 (vehicle) or 19 (T8Ab). Source data

As T8Ab was administered 24 h after ATII/PE initiation (before detectable cardiac dysfunction) these experiments primarily model disease prevention. To test efficacy against established hypertensive disease, treatment was delayed until after LV EF declined to 37% (Fig. 3c,d). While vehicle-treated mice showed no functional recovery (EF, 38%), T8Ab significantly improved EF (54%) and survival (Extended Data Fig. 2 and Supplementary Table 7). Thus, ANTXR1 blockade not only prevents but also reverses hypertensive cardiac damage, broadening its potential therapeutic scope.

We next tested T8Ab in an obesity-triggered HFpEF model^3^ (Fig. 3e). Mice were treated for 35 days with vehicle or T8Ab. As expected, EF% was unchanged, but fibrosis was reduced and diastolic function improved, with increased global longitudinal strain (GLS) and decreased mitral valve E/e′ ratios (Fig. 3f, Supplementary Fig. 5 and Supplementary Table 8). Because patients with HFpEF experience shortness of breath, lack of energy and exercise intolerance, we assessed treadmill performance. T8Ab-treated mice ran significantly longer than controls (Fig. 3f). Collectively, these results show that ANTXR1 inhibition improves cardiac function across diverse models of MI, hypertension and HFpEF.

### T8Ab cardioprotection post-MI

**Fig. 4:**
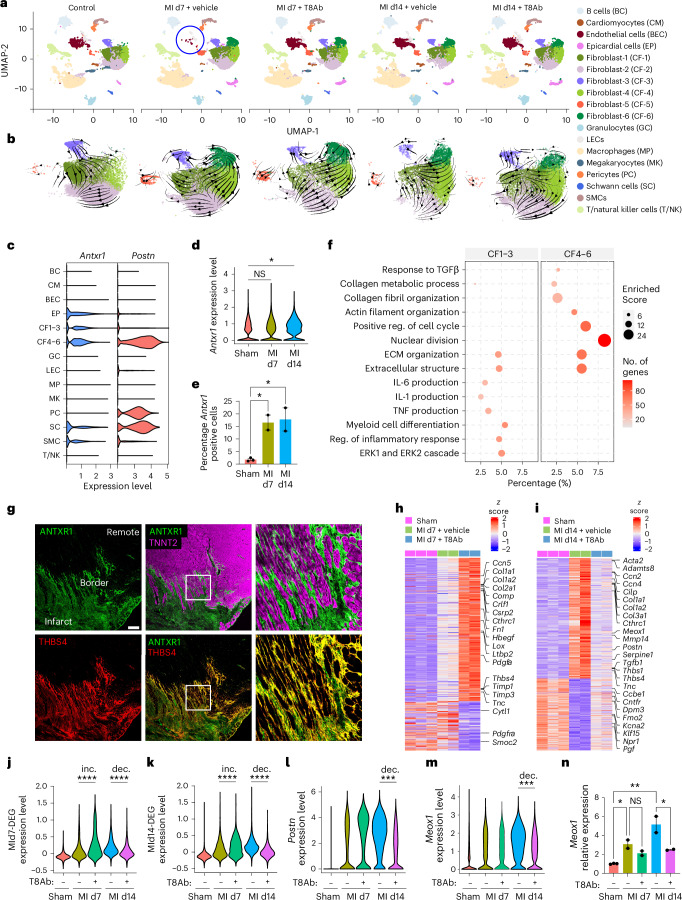
T8Ab treatment following MI promotes a cardioprotective gene signature. **a**, Uniform Manifold Approximation and Projection (UMAP) display of cardiac cell subpopulations. The diminished blood EC (BEC) population at MI d7 is highlighted (blue circle). **b**, Trajectory analysis of the six CF subclusters. **c**, Violin plot showing *Antxr1* and *Postn* expression in each annotated cell type. **d**, Violin plot showing the total *Antxr1* mRNA expression in CFs from MI d7/14 versus sham. **e**, Bar graph showing % increase of *Antxr1*-positive CF4–6 cells after MI d7/14 versus sham. *n* = 3 per group (sham) or 2 per group (MI samples), with each sample derived by pooling two hearts to minimize variability. **f**, GO analysis comparing pathway activation in CF1–3 versus CF4–6 at MI d7. Reg., regulation. **g**, Co-IF staining for ANTXR1 (green), THBS4 (red) and TNNT2 (magenta) in LV on day 7 post-MI. Image is representative of at least four hearts. Scale bar, 200 µm. **h**,**i**, Heat maps showing gene expression changes in the MI d7 + T8Ab and MI d7 + vehicle groups compared to sham (**h**) and the MI d14 + T8Ab and MI d14 + vehicle groups compared to sham (**i**). **j**,**k**, Violin plots showing the average expression of all genes upregulated by T8Ab at MI d7 (**j**) and downregulated by T8Ab at MI d14 (**k**) in **h** and **i**, respectively. inc., increased; dec., decreased. **l**,**m**, Violin plots showing *Postn* (**l**) and *Meox1* (**m**) expression. **n**, qRT–PCR validation of *Meox1* mRNA expression. *n* = 3 per group (sham) or 2 per group (MI samples), with each sample derived by pooling two hearts to minimize variability. Data are shown as mean ± s.e.m. One-way ANOVA (RT–PCR) with Tukey’s multiple-comparisons test (**e**,**n**) or two-tailed Wilcoxon test (violin plots) (**d**,**j**,**k**–**m**). *P < 0.05, **P < 0.01, ***P < 0.001, ****P < 0.0001. NS, nonsignificant. Source data

**Extended Data Fig. 3:**
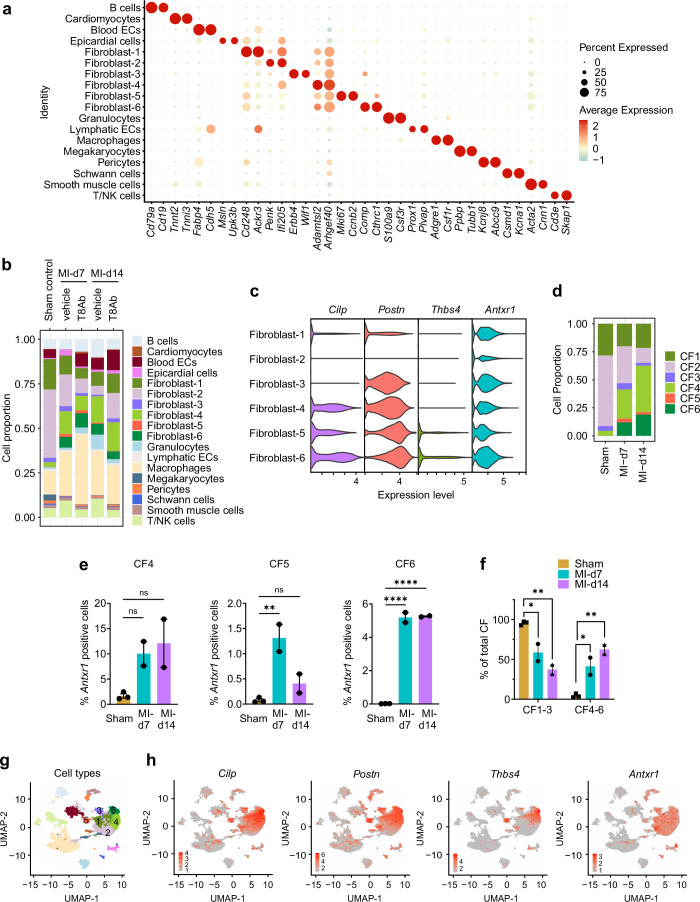
Single-cell characterization of cell populations following myocardial infarction. **a**) A dot plot displays enriched markers for each major cell population. **b**) Proportions of each major cell cluster across the 5 scRNA-seq groups. **c**) Violin plots showing the relative expression of *Cilp*, *Postn*, *Thbs4* and *Antxr1* in each of the 6 cardiac fibroblast subclusters. **d**) Changes in the proportions of each cardiac fibroblast (CF) subcluster in response to MI. **e**) Bar graph showing % increase of *Antxr1*-positive CF4, CF5 and CF6 cells after MI-day 7/14 versus sham. **f**) Perturbations in the CF1-3 and CF4-6 fractions as a % of the total CF population in the Sham, MI-d7 or MI-d14 groups. *P < 0.05, **P < 0.01. **g**) UMAP plot of subclustered cells from all groups in the MI study combined. The key for the color annotations is shown in B, and the six cardiac fibroblasts subclusters are numbered. **h**) UMAP plots showing the relative mRNA expression of *Cilp*, *Postn*, *Thbs4* and *Antxr1* in each of the cell populations. Source data

To gain insight into the mechanisms by which ANTXR1 regulates heart function, next we collected scRNA-seq data on 117,481 cardiac cells derived from five experimental groups using the 10x Genomics platform: sham + vehicle control, MI-d7 + vehicle, MI-d7 + T8Ab, MI-d14 + vehicle and MI-d14 + T8Ab (Fig. 2d). Unsupervised clustering of the scRNA-seq data revealed various cardiac cell subpopulations, including CFs, endothelial cells (ECs), smooth muscle cells (SMCs), macrophages and other hematopoietic cells, as well as a small population of cardiomyocytes (Fig. 4a and Extended Data Fig. 3a,b). The CF population consisted of six subclusters (CF1–6), all of which expressed *Antxr1* mRNA and could be further subdivided into resting (CF1–3) and activated (CF4–6) subpopulations, only the latter of which increased in relative proportion post-MI and expressed the activation markers *Thbs4*, *Postn* and *Cilp* (Fig. 4b,c and Extended Data Fig. 3c–f). While *Antxr1* mRNA was not found in hematopoietic cells, it was detectable in other mesenchymal cells, including SMCs, epicardial cells and Schwann cells (Fig. 4c).

The most prominent population shift following MI was the expansion of the CF4–6 clusters at both days 7 and 14 post-MI (Fig. 4b and Extended Data Fig. 3d–f). This expansion of the *Antxr1*-positive CF4–6 populations was consistent with the increased total ANTXR1 protein in infarcted hearts (compare Fig. 1g with Fig. 4d,e). Gene Ontology (GO) analysis revealed an enrichment of TGFβ and ECM organization-related pathways in CF4–6, whereas the inflammatory and ERK1/2 pathways were enriched in CF1–3 (Fig. 4f), suggesting functional specialization among CF subsets in HF. To determine whether ANTXR1 protein was expressed in the expanding CF4–6 population, co-IF staining was performed for ANTXR1 and THBS4, a marker of activated CF4–6 cells^24^ (Fig. 4g). At 7 days post-MI, ANTXR1 was highly colocalized with THBS4 in the LV scar region (Fig. 4g), whereas THBS4 negative CFs showed little or no ANTXR1 signal, despite detectable *Antxr1* mRNA across all CF subsets. These findings suggest that ANTXR1 protein translation occurs predominantly in *Thbs4*-positive CF4–6 cells, indicative of post-transcriptional regulation, as described for other profibrotic proteins^25^. Although *Antxr1* transcripts were also detected in epicardial cells, Schwann cells and SMCs, T8Ab treatment induced minimal to no transcriptional changes in these populations (Supplementary Fig. 6 and Supplementary Table 9). Hematopoietic cells, which lack *Antxr1* expression, were similarly unaffected (Supplementary Fig. 7). Together, these findings indicate that ANTXR1 is functioning at the protein level most prominently in the CF4–6 population that rapidly expands by 7 days after MI, providing a potential marker for activated CFs in the ischemic heart.

**Extended Data Fig. 4:**
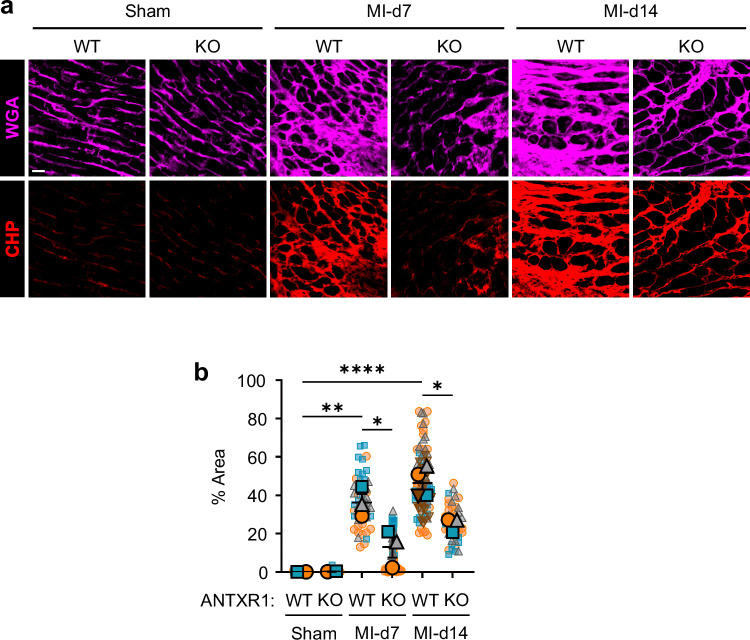
ANTXR1 promotes collagen remodeling following myocardial infarction. **a**) Immunofluorescence staining with a collagen hybridizing peptide (CHP, red) was used to evaluate the levels of denatured collagen in the left ventricular wall of ANTXR1 wildtype (WT) and knockout (KO) at day 7 or 14 post-myocardial infarction (MI). All cardiac fibroblasts were labeled with wheat germ agglutinin (WGA, magenta). Bar = 20 µm. **b**) Quantification of staining shown in A. Data represent the mean ± SEM from independent hearts samples indicated by the use of different colors/shapes. For each sample, multiple fields were imaged (small symbols), and the larger symbol corresponds to the average of that sample. *P* values were assessed using a one-way ANOVA with Tukey’s multiple-comparisons test. *P < 0.05, **P < 0.01, ****P < 0.0001. Source data

Next, we examined gene expression alterations in CFs in response to MI and T8Ab treatment. As expected, MI triggered increased expression of ECM components involved in scar formation, including *Col1a1*, *Col1a2* and *Fn1* (Supplementary Fig. 8). Notably, T8Ab treatment further augmented ECM gene expression by day 7, suggesting that ANTXR1 antagonism accelerates early scar formation (Fig. 4h). T8Ab also rapidly upregulated matricellular proteins involved in scar stabilization, including *Ccn5*, *Comp*, *Cthrc1*, *Pdgfa*, *Thbs4* and *Timp3*, which have been linked to protective matrix remodeling and prevention of early cardiac rupture^26–32^ (Fig. 4h and Supplementary Table 10). In support of a matrix-preserving effect, ANTXR1 ablation also prevented collagen degradation in the MI model (Extended Data Fig. 4). In contrast, genes downregulated by T8Ab treatment, such as *Cytl1*, *Pdgfra* and *Smoc2*, have previously been found to promote cardiotoxicity^33–35^ (Supplementary Table 10). GO analysis further confirmed enhanced ECM production by T8Ab on day 7 (Supplementary Fig. 9a). By day 14 all major pathways that remained activated by MI, including TGFβ induction and collagen production, had reverted back to a baseline state following T8Ab treatment (Supplementary Fig. 9b). To test whether TGFβ signaling was ANTXR1-dependent in vivo, IF staining for nuclear phospho-SMAD3 was performed, revealing reduced SMAD3 activation in *Antxr1* KO hearts on day 14 (Supplementary Fig. 10).

Several of the T8Ab induced cardioprotective genes known to facilitate rapid scar development, such as *Col1a1*, *Col1a2* and *Col3a1*, are also implicated in maladaptive fibrosis during chronic remodeling. Surprisingly, many of these genes were significantly downregulated by T8Ab on day 14 (Fig. 4h,i). Reanalysis of T8Ab-regulated gene sets showed that day 7-upregulated genes (*n* = 331) were globally downregulated at day 14, whereas day 14-downregulated genes (*n* = 294) were relatively elevated on day 7 (Fig. 4j,k), highlighting a time-dependent, bidirectional effect of ANTXR1 blockade. A representative example is *Postn*, encoding periostin, a matricellular protein essential for early scar formation but also a driver of late-stage maladaptive cardiac fibrosis^36,37^, which was induced on day 7 and suppressed on day 14 (Fig. 4l). Moreover, T8Ab also prevented the MI-induced downregulation of several cardioprotective genes, including *Klf15*, *Npr1* and *Pgf* on day 14^38–40^ (Fig. 4i and Supplementary Table 10). Together, these results indicate that ANTXR1 modulates ECM homeostasis and that its inhibition promotes early scar stabilization while limiting late-stage maladaptive fibrosis.

MEOX1, a transcription factor activated through TGFβ signaling, has emerged as a central regulator of fibroblast activation in cardiac disease^41^. While BET bromodomain inhibitors were able to block MEOX1, reversing CF activation and HF in mouse models, their broad off-target effects in normal tissues poses challenges for their clinical application^41^. Notably, T8Ab treatment selectively suppressed *Meox1* expression in CFs on day 14, a finding confirmed by RT–PCR (Fig. 4m,n). These studies highlight the potential of T8Ab to reverse MEOX1-driven fibrosis without widespread off-target effects.

Another prominent alteration caused by MI was a transient reduction in the size of the blood EC (BEC) cluster by day 7, which recovered by day 14, whereas the nearby lymphatic EC (LEC) cluster remained unchanged (Fig. 4a). T8Ab treatment preserved blood EC numbers on day 7 (Supplementary Fig. 11a). EC loss may result from injury-induced endothelial-to-mesenchymal transition (EndMT), which increases collagen-producing myofibroblasts at the expense of impaired angiogenesis^26,42,43^. Consistent with EndMT, the blood EC cluster in the infarcted heart showed reduced expression of endothelial genes (for example *Kdr*, *Flt* and *Fabp4*), and increased expression of mesenchymal genes (for example *Fn* and *Vim*) (Supplementary Fig. 11b,c). T8Ab largely prevented these transcriptional changes at both time points and preserved cardioprotective EC genes including *Egln1*, *Timp3*, *Timp4*, *Epas1*, *Flt1* and *Bmp6*^44–49^ (Supplementary Table 10). Moreover, angiogenesis-related pathways were enriched in CFs following T8Ab treatment by day 7 (Supplementary Fig. 11b), and IF staining revealed increased vascular density following ANTXR1 antagonism (Supplementary Fig. 11d,e). Although *Antxr1* mRNA expression was negligible in cardiac macrophages (Fig. 4c), T8Ab treatment increased macrophage cell numbers on day 7 but not day 14, suggesting an indirect modulation of this cell population (Supplementary Fig. 7a). On day 7, T8Ab treatment also altered macrophage gene expression, including activation of pro-angiogenic pathways (Supplementary Fig. 12).

### T8Ab cardioprotection post-hypertension

**Fig. 5:**
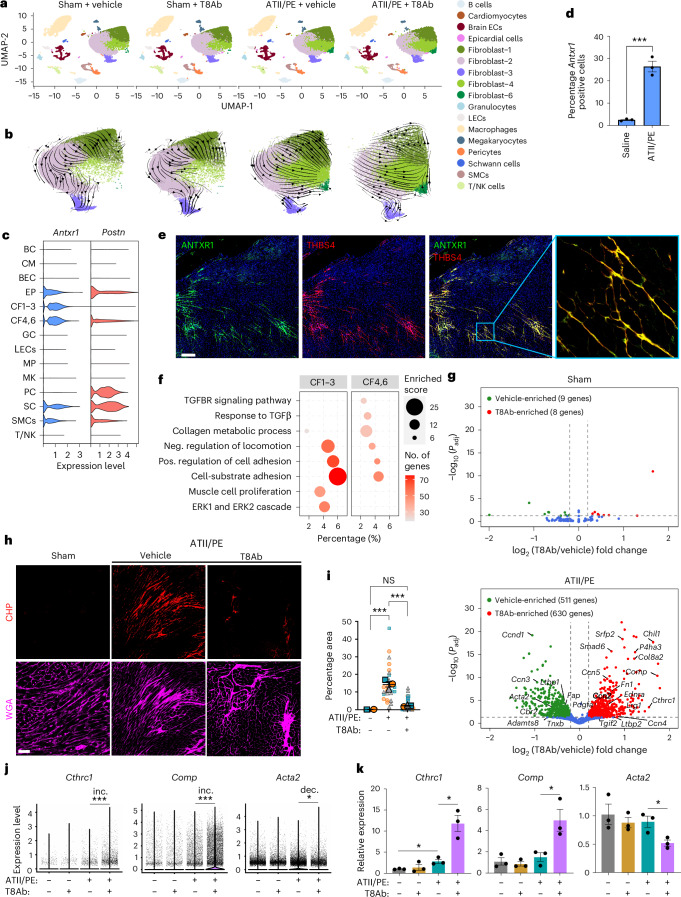
T8Ab treatment promotes a cardioprotective gene signature in hypertension. **a**, UMAPs display cardiac cell subpopulations for each treatment group. **b**, Trajectory analysis of the CF subclusters. **c**, Violin plot showing the relative *Antxr1* and *Postn* expression in each annotated cell type. **d**, Bar graph indicates % increase in *Antxr1*-positive CF4,6 cells versus all the cells after 28 days of ATII/PE. *n* = 3 per group, and each individual sample was derived by pooling together two hearts to minimize variability. Data represent mean ± s.e.m. **e**, Co-IF staining for ANTXR1 (green) and THBS4 (red) in the LV 28 days post-ATII/PE. Image is representative of at least three hearts. Scale bar, 200 µm. **f**, GO analysis comparing enriched pathways in CF1–3 versus CF4,6 post-ATII/PE exposure. **g**, Volcano plot showing gene expression changes in response to T8Ab treatment in the sham control (top) and ATII/PE treated (bottom) groups. **h**, Co-IF staining for CHP (red) and WGA (magenta) in LV after 28 days of ATII/PE. Image is representative of at least three hearts. Scale bar, 50 µm. **i**, Quantification of CHP staining. Data represent mean ± s.e.m. from three independent biological samples, each depicted with a distinct color/shape. For each sample, multiple fields were imaged (small symbols), and the larger symbol corresponds to the average of that sample. **j**, Violin plot showing *Cthrc1*, *Comp* and *Acta2* expression. **k**, qRT–PCR validation of *Cthrc1*, *Comp* and *Acta2* mRNA expression. *n* = 3 per group, and each individual sample was derived by pooling together two hearts to minimize variability. *P* values were assessed using an unpaired, two-tailed *t*-test (**d**), one-way ANOVA with Tukey’s multiple-comparisons test (**i**,**k**) or two-tailed Wilcoxon test (**j**). *P < 0.05, ***P < 0.001. Source data

**Extended Data Fig. 5:**
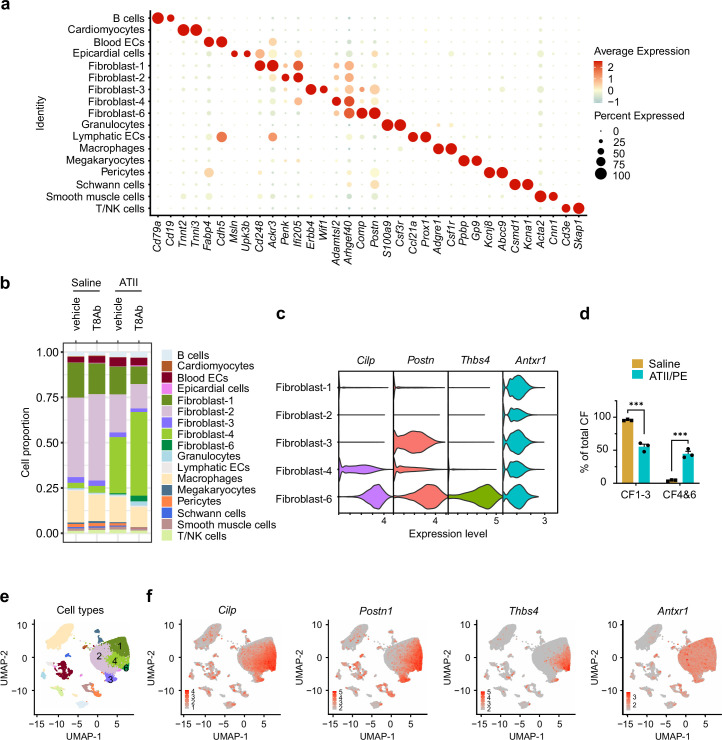
Single-cell characterization of cardiac cell populations following ATII/PE. **a**) Dot plot displaying enriched markers for each major cell population. **b**) Proportions of each major cell cluster across the 4 scRNA-seq groups. **c**) Violin plots showing the relative expression of *Cilp*, *Postn*, *Thbs4* and *Antxr1* in each of the indicated cardiac fibroblast subclusters. **d**) Perturbations in the CF1-3 and CF4&6 clusters as a % of the CF population caused by ATII/PE treatment compared to sham controls. ***p < 0.001. **e**) UMAP plot of subclustered cells from all groups in the ATII/PE study combined. The key for the color annotations is shown in **b** and numbers indicate the cardiac fibroblast subclusters. **f**) UMAP plots showing relative mRNA expression of *Cilp*, *Postn*, *Thbs4* and *Antxr1* in each of the cell populations. Source data

To explore the impact of ANTXR1 in cardiac function under hypertensive stress, scRNA-seq was performed on hearts subjected to 4 weeks of ATII/PE, with or without T8Ab treatment (Fig. 3a). In total, 106,443 processed cells were analyzed and, as in the MI model, all major cardiac cell types were identified by unsupervised clustering (Fig. 5a and Extended Data Fig. 5a,b). However, the small CF5 cluster, which was enriched post-MI and marked by high expression of cell cycle genes, was absent following ATII/PE, likely reflecting a reduced population of cycling cells. Trajectory analysis suggested that the CF4 and CF6 populations, which expanded upon ATII/PE treatment, likely originated from the CF1–3 clusters (Fig. 5b), consistent with observations from the MI model. Although *Antxr1* mRNA was again detected in epicardial cells, Schwan cells, SMCs and all fibroblast subclusters, only the activated CF4 and CF6 populations expanded following ATII/PE treatment, leading to an overall increase in *Antxr1*-positive CFs (Fig. 5c,d, and Extended Data Fig. 5c–f). Co-IF revealed strong ANTXR1 and THBS4 colocalization within the expanding CF population following hypertension (Fig. 5e), recapitulating the pattern observed post-MI. Gene expression profiling of the CF4 and CF6 subsets revealed upregulation of pathways associated with TGFβ signaling and collagen biosynthesis, which were absent in CF1–3 (Fig. 5f). As in the MI model, ATII/PE treatment induced robust ECM production and activation of the TGFβ pathway in CFs (Supplementary Fig. 13).

T8Ab treatment in the ATII/PE model led to the upregulation of 630 genes and downregulation of 511 genes in CFs, with minimal effects in sham controls (Fig. 5g). Several upregulated genes overlapped with those induced in the MI model, including *Ccn2*, *Ccn4*, *Ccn5*, *Comp*, *Cthrc1* and *Pdgfa;* all associated with cardioprotective fibrosis^26–28,30,50–52^ (Fig. 5g, bottom and Supplementary Table 10). To determine whether the T8Ab was also able to block collagen degradation, CHP IF staining was performed. ATII/PE treatment induced a ~15-fold increase in collagen degradation, which was reduced to near baseline levels by T8Ab (Fig. 5h,i). Some genes involved in proliferation, myofibroblast formation and/or fibrosis were also reduced by T8Ab treatment such as *Ccnd1*, *Acta2* (αSMA), *Fap*, *Ccn3* and *Adamts8* (refs. ^53–55^) (Fig. 5g, bottom and Supplementary Table 10). RT–qPCR validated both upregulated and downregulated transcripts identified in the scRNA-seq dataset (Fig. 5j,k). In sharp contrast to the post-injury response, treatment of sham controls with T8Ab had little to no impact on cell proportions (Extended Data Fig. 5b) and gene expression in CFs (Fig. 5g, top) or all cardiac cells (Supplementary Fig. 14), suggesting that ANTXR1 activity is preferentially engaged under pathological conditions.

### ANTXR1 functions in cardiac fibroblasts

**Fig. 6:**
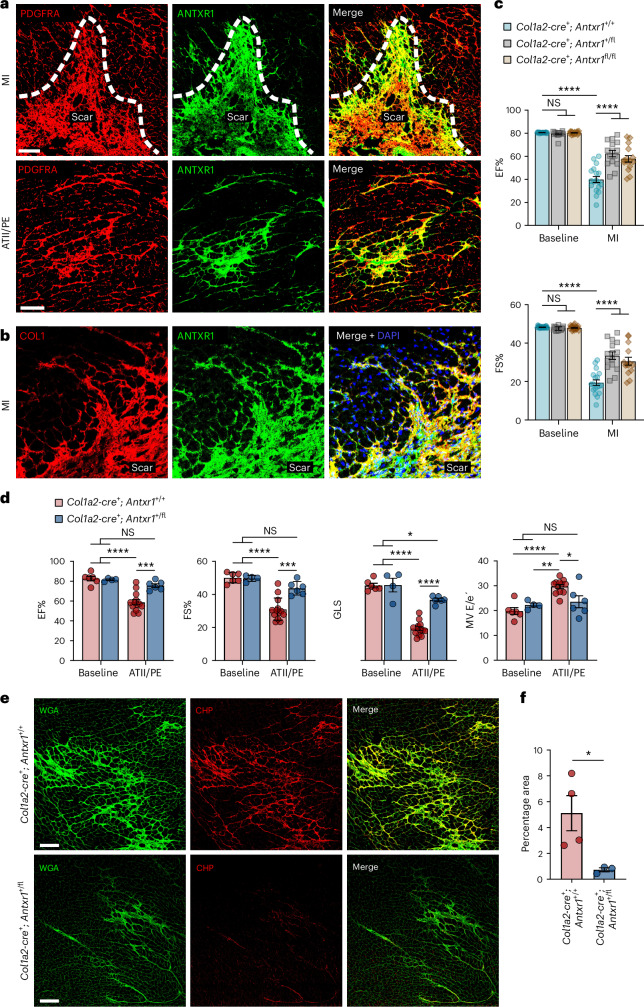
ANTXR1-positive cardiac fibroblasts promote heart damage in vivo. **a**, Co-IF staining for ANTXR1 (green) and PDGFRA (red) in the LV 14 days following MI or 28 days of treatment with ATII/PE. Scale bar, 100 µm. **b**, Co-IF staining for ANTXR1 (green) and COL1 (red) in the LV 14 days following MI. Image is representative of at least three biological specimens. Scale bar, 100 µm. **c**, Cardiac EF% and FS% 5 weeks post-MI in fibroblast conditional *Antxr1* KO mice. *n* = 18 (*Antxr1*^+/+^ and *Antxr1*^+/fl^) or 19 (*Antxr1*^fl/fl^) mice per group at baseline. **d**, Cardiac EF%, FS%, GLS and ratio of early diastolic mitral inflow velocity to early diastolic mitral annulus velocity (MV E/e’) following 28 days of ATII/PE treatment in fibroblast conditional KO mice. Baseline: *n* = 6 (*Antxr1*^+/+^) or 4 (*Antxr1*^+/fl^); ATII/PE: *n* = 13 (*Antxr1*^+/+^) or 6 (*Antxr1*^+/fl^)/mice group. **e**, Co-IF staining for CHP (red) and WGA (green) in LV after 28 days of ATII/PE. Scale bar, 100 µm. **f**, Quantification of CHP staining. *n* = 4 (*Antxr1*^+/+^) or 3 (*Antxr1*^+/fl^) hearts per group. Results represent mean ± s.e.m. *P* values were derived from a one-way ANOVA with Tukey’s post hoc test (**c**,**d**) or an unpaired, two-tailed *t*-test (**f**). *P < 0.05, **P < 0.01, ***P < 0.001, ****P < 0.0001. Source data

**Extended Data Fig. 6:**
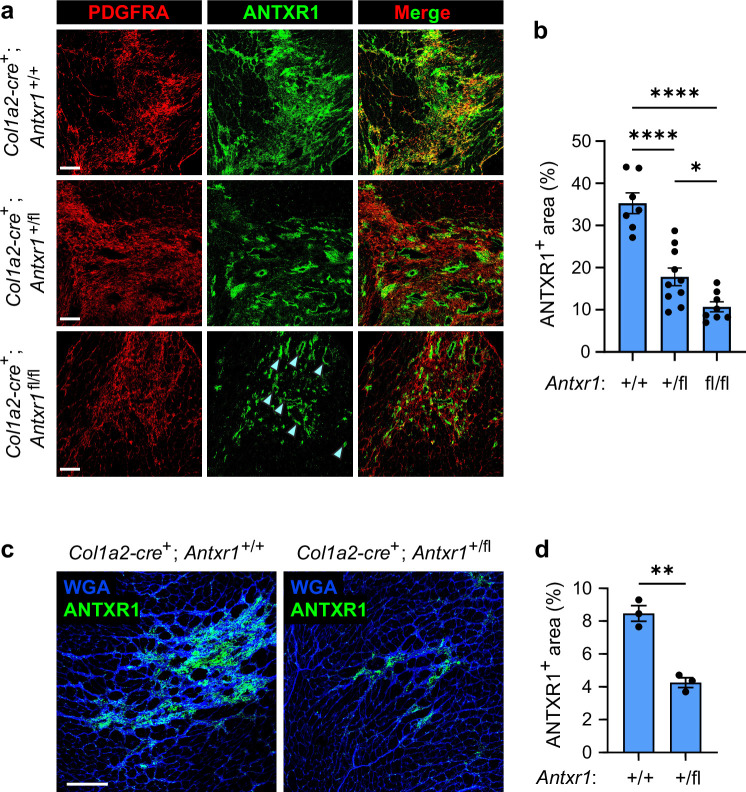
ANTXR1 expression in cardiac fibroblasts of conditional KO mice. To evaluate ANTXR1 expression, immunofluorescence staining was performed on cardiac tissue from fibroblast-specific conditional KO mice expressing the tamoxifen-inducible *Col1a2-cre* transgene. **a**) PDGFRA (red), a marker of fibroblasts, and ANTXR1 (green) were examined in the LV infarct region 5 weeks post-MI. Arrowheads mark ANTXR1 expression in vascular cells where *Antxr1* deletion is not expected. Image is representative of 3 independent specimens per genotype. **b**) quantification of the ANTXR1^+^ area as a percentage of the total PDGFRA^+^ (fibroblast) area. **c**) WGA (blue) and ANTXR1 (green) were examined in the LV of hypertensive hearts 28 days post-ATII/PE commencement. Image is representative of 3 independent specimens per genotype. **d**) quantification of the ANTXR1^+^ area as a percentage of the total area. Bar = 100 µm. Data in (**b**) and (**d**) represent the mean ± SEM. *P < 0.05, **P < 0.01, ****P < 0.0001. P values were calculated using one-way ANOVA with Tukey’s multiple-comparisons test (**b**) or a two-tailed t-test (**d**). Source data

The prominent gene expression alterations in CFs following T8Ab treatment suggest that *Antxr1*-positive CFs are the principal effectors of ANTXR1 activity in cardiac disease. Co-IF staining further supported this, and demonstrated robust colocalization of ANTXR1 with CF markers, including THBS4, PDGFRA and collagen (Figs. 4g, 5e and 6a,b). Although ANTXR1 was detected in a subset of vascular cells under both MI and hypertensive conditions (Supplementary Fig. 15), the most extensive colocalization occurred in CFs. To directly test the role of CFs in mediating ANTXR1 function in vivo, we generated fibroblast-specific *Antxr1* knockout mice using the tamoxifen-inducible Col1a2-CreER conditional KO strain. Following confirmation of tamoxifen-inducible cre expression in CFs of Col1a2-cre; mTmG reporter mice (Supplementary Fig. 16), cohorts of three genotypes were generated for comparison: *Col1a2-cre*^*+*^; *Antxr1*^*+/+*^ (fibroblast WT; Fib-WT) and *Col1a2-cre*^*+*^; *Antxr1*^*+/fl*^ (heterozygous; Fib-Het) and *Col1a2-cre*^*+*^; *Antxr1*^*fl/fl*^ (KO; Fib-KO). All animals were maintained on a tamoxifen diet to control for potential off-target effects. Cardiac function post-MI was significantly improved in both ANTXR1 Fib-Het and Fib-KO mice compared to Fib-WT controls (Fig. 6c and Supplementary Table 11). Of note, Fib-Het mice showed improvements comparable to those of Fib-KOs, suggesting that ANTXR1 deleterious activity may depend on expression exceeding a critical threshold. Under hypertensive conditions, ANTXR1 Fib-Het mice similarly exhibited improved cardiac function relative to Fib-WT controls (Fig. 6d and Supplementary Table 12). IF staining confirmed reduced ANTXR1 expression in CFs from both Fib-Het and Fib-KO mice (Extended Data Fig. 6) and CHP staining demonstrated decreased denatured collagen in ANTXR1 Fib-Het hearts (Fig. 6e,f). While minor contributions from other cell types cannot be excluded, these data strongly support ANTXR1-positive CFs as the key mediators of ANTXR1 activity in vivo.

### ANTXR1 facilitates TGFβ signaling

**Fig. 7:**
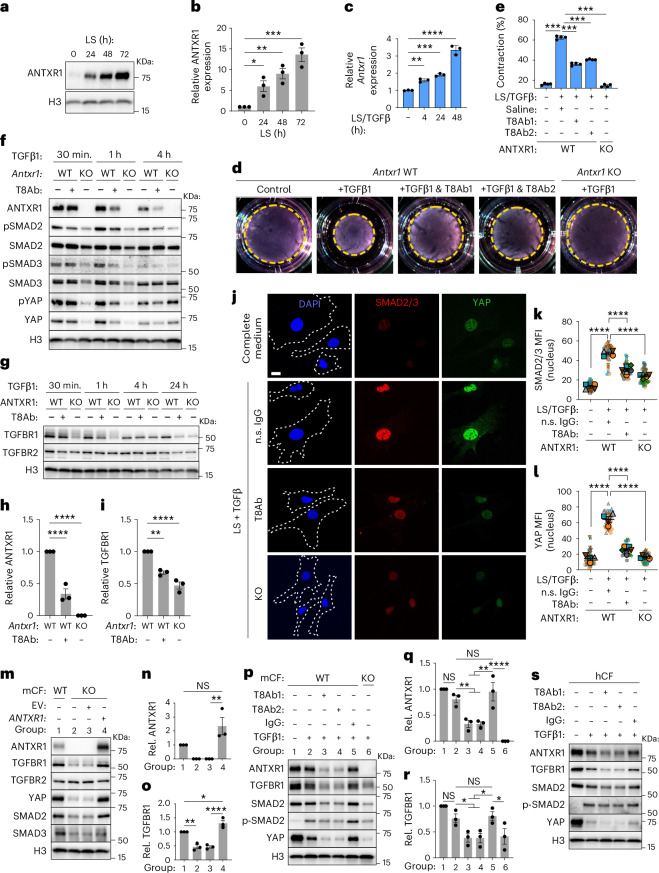
T8Ab blocks TGFβ signaling in primary CFs. **a**, Immunoblot of ANTXR1 in CFs under low serum (LS) conditions. **b**, Quantification of ANTXR1 under LS conditions. *n* = 3 biological replicates from independent experiments including the immunoblot in **a**. **c**, RT–PCR analysis of *Antxr1* mRNA expression in CFs in response to LS/TGFβ treatment. *n* = 3 biological replicates. **d**, Gel contraction assay showing the impact of T8Ab treatment on CF response to TGFβ1. T8Ab1: L2, T8Ab2: YL9. **e**, Quantification of the gel contraction assay. *n* = 4 biological replicates from independent experiments. Control, nonstimulated. **f**, Immunoblot of the TGFβ signaling pathway in ANTXR1 WT and KO CFs following LS/TGFβ1 treatment. **g**, Immunoblot of TGFBRs in ANTXR1 WT and KO CFs treated with LS/TGFβ1. **h**,**i**, Quantification of the ANTXR1 (**h**) and TGFBR1 (**i**) protein level in CFs following 1 h of TGFβ1 treatment. *n* = 3 biological replicates and includes the immunoblots shown in **f** and **g**. **j**, SMAD2/3 (red) and YAP (green) IF staining of ANTXR1 WT (top three rows) or KO (bottom row) CFs 1 h post-treatment with LS/TGFβ1. n.s. IgG, nonspecific IgG. Scale bar, 20 µm. **k**,**l**, Quantification of SMAD2/3 (**k**) and YAP (**l**) IF staining. *n* = 4 per group (WT control and WT IgG), 6 (WT +T8Ab) or 5 (KO) independent biological replicates, each depicted with a distinct color or shape. For each sample, multiple fields were imaged (small symbols), and the larger symbol corresponds to the average of that sample. **m**, Rescue of TGFβ signaling by restoring ANTXR1 expression in the KO CFs. EV, empty vector. **n**,**o**, Quantification of the ANTXR1 (**n**) and TGFBR1 (**o**) protein level in CFs following ANTXR1 rescue. *n* = 3 biological replicates and includes the immunoblot shown in **m**. **p**, Immunoblot comparing T8Ab1 (L2) and T8Ab2 (YL9) activity on TGFβ signaling in *Antxr1* WT mouse CFs (mCFs). *Antxr1* KO CFs were used as a control. **q**,**r**, Quantification of the ANTXR1 (**q**) and TGFBR1 (**r**) protein level in CFs following ANTXR1 antibody blockade. *n* = 3 biological replicates and includes the immunoblot shown in **p**. **s**, The effect of T8Abs on TGFβ signaling in hCFs. For immunoblots, H3 antibody served as a loading control. Results represent mean ± s.e.m. (**b**,**h**,**i**,**k**,**l**,**n**,**o**,**q**,**r**) or mean ± s.d. (**c**,**e**) from independent biological replicates. *P* values were derived by one-way ANOVA with Tukey’s multiple-comparisons test (**b**,**c**,**e**,**h**,**i**,**k**,**l**,**n**,**o**,**q**,**r**). *P < 0.05, **P < 0.01, ***P < 0.001, ****P < 0.0001. Source data

To further elucidate the mechanistic role of ANTXR1 in cardiac fibrosis, immortalized Antxr1 WT primary CFs from *Antxr1* conditional KO mice were established and treated with adeno-cre to create an *Antxr1* KO subline for comparison. Consistent with previous findings in CAFs^9^, ANTXR1 expression was induced under low serum (LS; 0.5%) conditions compared to 10% serum (Fig. 7a,b). Given the central role of TGFβ in CF activation, myofibroblast differentiation and fibrosis^6,56,57^, and its modulation by T8Ab in both MI and hypertension models (Figs. 4f and 5f and Supplementary Figs. 9b, 10 and 13) we next investigated the impact of ANTXR1 on TGFβ signaling. TGFβ treatment of serum-deprived CFs for 4, 24 or 48 h induced robust upregulation of canonical profibrotic genes (*Acta2*, *Col1a1, Cthrc1* and *Postn*) as well as *Antxr1* itself (Fig. 7c and Supplementary Fig. 17a). Induction of ACTA2 protein, the original myofibroblast marker, and activation of SMAD and YAP pathways were confirmed by immunoblotting (Supplementary Fig. 17b). These data confirmed the successful reprogramming of our established primary CFs into a myofibroblast-like state by TGFβ, as previously reported^58,59^.

Next, the role of ANTXR1 on TGFβ functional activity was evaluated using a collagen gel contraction assay, a mechanosensing assay that measures the ability of CFs to remodel their surrounding collagen I matrices in response to TGFβ. As expected^41,56,60^, TGFβ induced robust gel contraction (Fig. 7d,e). T8Abs largely blocked TGFβ induced collagen gel contraction in WT CFs, whereas CF-ANTXR1-KO cells were unresponsive to TGFβ, further confirming the requirement for ANTXR1 in mediating this signaling axis. MMP14, a key effector of TGFβ-driven collagen remodeling^61,62^, was also reduced upon ANTXR1 blockade (Supplementary Fig. 18). Next, immunoblotting was used to evaluate the role of ANTXR1 in TGFβ signaling activity in CFs (Fig. 7f–i and Supplementary Fig. 19). T8Ab treatment reduced total and phosphorylated levels of SMAD2, SMAD3 and YAP, major downstream mediators of TGFβ signaling under mechanical stress^63,64^. Genetic ablation of *Antxr1* also blocked SMAD2/3 and YAP, but in a more accelerated manner. Inhibition or deletion of YAP specifically in CFs has been shown to reduce fibrosis and improve cardiac function, following MI and hypertension^65,66^. TGFβ downstream signaling was further validated in cultured CFs by IF staining where both antibody treatment and genetic loss of *Antxr1* significantly abolished the nuclear localization of active SMAD2/3 and YAP (Fig. 7j–l). YAP nuclear levels were also decreased in vivo in response to ANTXR1 antagonism 14 days post-MI (Supplementary Fig. 20). These data identify ANTXR1 as a critical mediator of the TGFβ-SMAD2/3-YAP profibrotic signaling axis in CFs.

### ANTXR1 stabilizes TGFβ receptor 1

**Extended Data Fig. 7:**
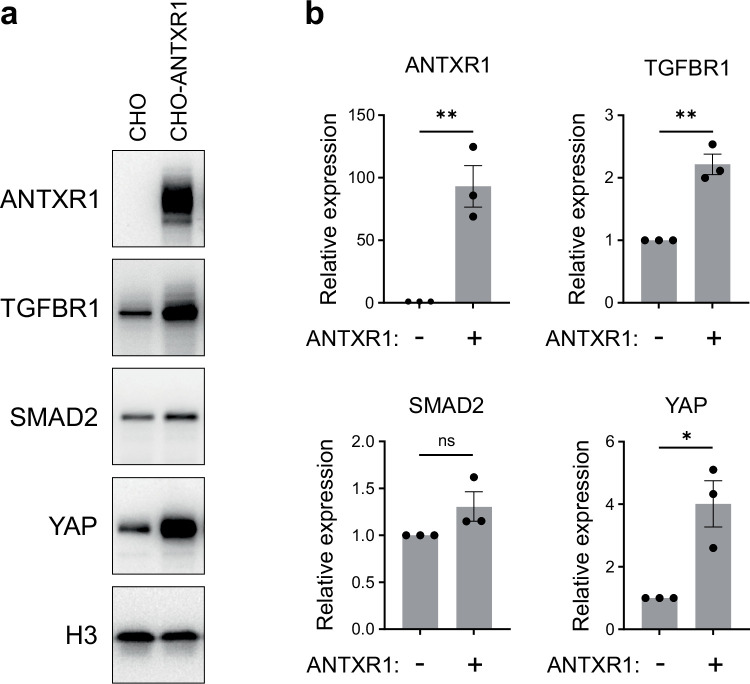
Ectopic overexpression of ANTXR1 stabilizes TGFBR1 levels in CHO cells. **a**) Immunoblots showing ANTXR1, TGFBR1, SMAD2 and YAP in CHO and CHO-ANTXR1 cells. H3 was used as a loading control. **b**) Quantification of the proteins shown in (**a**) from three independent experiments. Source data

**Extended Data Fig. 8:**
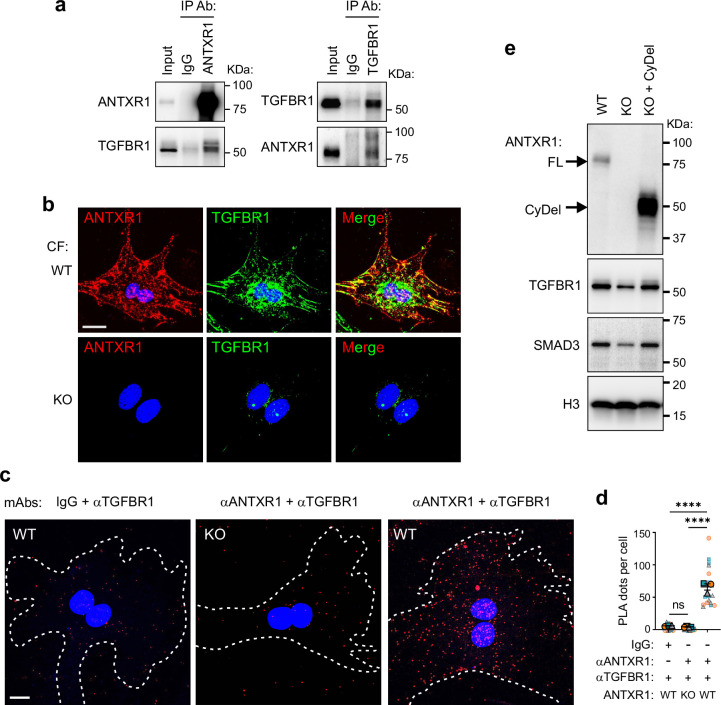
ANTXR1 and TGFBR1 are closely associated in CF. **a**) Immunoblotting of coImmunoprecipitation (IP) experiments assessing the interaction between ANTXR1 and TGFBR1 in CF. Non-specific IgG was used as a non-binding control. Results are representative of at least 3 independent experiments. **b**) Co-immunofluorescence staining assessing ANTXR1 (red) and TGFBR1 (green) in ANTXR1 wildtype (WT) and knockout (KO) cardiac fibroblasts (CF). Bar: 20 µm. **c**) Representative images of a proximity ligation assay testing interactions between ANTXR1 and TGFBR1 on the surface of live ANTXR1 WT and KO CFs. Bar: 20 µm. **d**) Quantification of PLA assays. *P* values were assessed using a one-way ANOVA with Tukey’s multiple-comparisons test. Data represent the mean ± SEM from independent hearts samples indicated by the use of different colors/shapes. For each sample, multiple fields were imaged (small symbols), and the larger symbol corresponds to the average of that sample. ****P < 0.0001. ns, not significant. **e**) Immunoblots showing TGFBR1 and SMAD3 levels in ANTXR1 wildtype (WT), ANTXR1 knockout (KO), and ANTXR1 KO cardiac fibroblasts overexpressing ANTXR1 with a deletion of its cytosolic domain (ANTXR1-CyDel). H3 was used as a loading control. Results are representative of at least 3 independent experiments.

**Extended Data Fig. 9:**
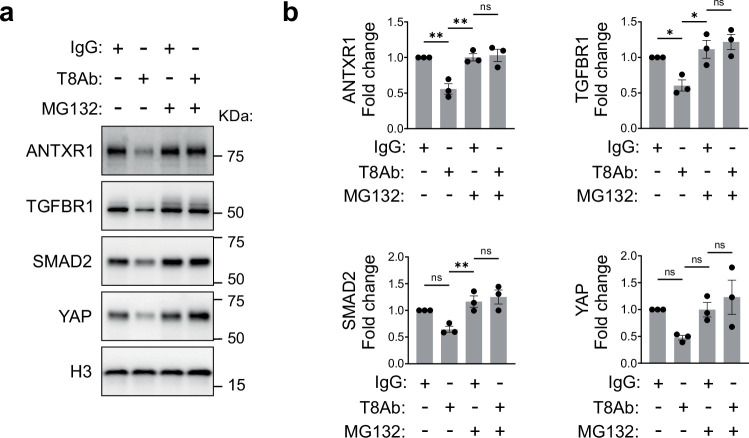
ANTXR1 protects TGFBR1 from proteasomal degradation. **a**) Immunoblots showing ANTXR1, TGFBR1, SMAD2 and YAP levels in response to treatment with T8Ab and the proteasome inhibitor MG132. H3 was used as a loading control. **b**) Quantification of the proteins shown in (**a**) from three independent experiments. Data represent the mean ± SEM. *P* values were assessed using a one-way ANOVA with Tukey’s multiple-comparisons test. *P < 0.05, **P < 0.01. ns, non-significant.

Intrigued by the dependency of TGFβ signaling on ANTXR1, we examined potential interactions with canonical TGFβ receptors. TGFBR1 protein levels were markedly reduced in *Antxr1* KO CFs and progressively diminished in T8Ab-treated *Antxr1* WT CFs (Fig. 7g,i). In contrast, TGFBR2 levels remained unchanged. Re-expression of ANTXR1 in KO cells restored TGFBR1 and downstream signaling components (Fig. 7m–o and Supplementary Fig. 19b). Induction of TGFBR1, SMAD and YAP protein was also observed following ectopic overexpression in ANTXR1 negative CHO cells (Extended Data Fig. 7). These data suggested a potential interaction between TGFBR1 and ANTXR1. Upon coimmunoprecipitation in CFs, TGFBR1 was detected in ANTXR1 immunoprecipitates and ANTXR1 was found in TGFBR1 immunoprecipitates (Extended Data Fig. 8a), indicating that ANTXR1 may stabilize TGFBR1 on the cell surface. To assess cell surface colocalization, co-IF staining was performed, which revealed a substantial overlap of ANTXR1 and TGFBR1 in ANTXR1 WT CFs (Extended Data Fig. 8b). TGFBR1 was undetectable at the surface of *Antxr1* KO CFs consistent with its downregulation in cellular lysates (compare Fig. 7m,o with Extended Data Fig. 8b). A proximity ligation assay further validated the interaction (Extended Data Fig. 8c,d). Expression of a truncated ANTXR1 lacking its cytosolic domain was sufficient to stabilize TGFBR1, suggesting that extracellular and/or transmembrane domains mediate this effect (Extended Data Fig. 8e). Next, we examined whether ANTXR1’s ability to stabilize TGFBR1 required increased transcription, but found no decrease in *Tgfbr1* mRNA following ANTXR1 antagonism (Supplementary Fig. 21); however, TGFBR1 protein loss following T8Ab treatment was fully rescued by the proteosome inhibitor MG132, indicating that ANTXR1 controls TGFBR1 stability at the post-translational level (Extended Data Fig. 9). Together, these results suggest that ANTXR1 regulates fibrosis at least in part through its interaction with TGFBR1 on the cell surface of CFs.

Like TGFBR1, ANTXR1 is highly conserved, with ~98% amino acid identity between mouse and human proteins. To evaluate the role of ANTXR1 in TGFβ signaling in human primary CFs (hCFs), we tested both T8Ab (L2; T8Ab1) and another cross-species (human/mouse) reactive fully human anti-ANTXR1 antibody, clone YL9 (T8Ab2), which we determined could also block TGFβ signaling in mouse CFs (Fig. 7p–r and Supplementary Fig. 19c). Epitope mapping revealed that both antibodies targeted a similar region on the ANTXR1 extracellular domain (Supplementary Fig. 22). Treatment with both ANTXR1 antibodies decreased TGFβ signaling in hCFs, resulting in decreased levels of TGFBR1, SMAD2/3 and YAP (Fig. 7s).

**Fig. 8:**
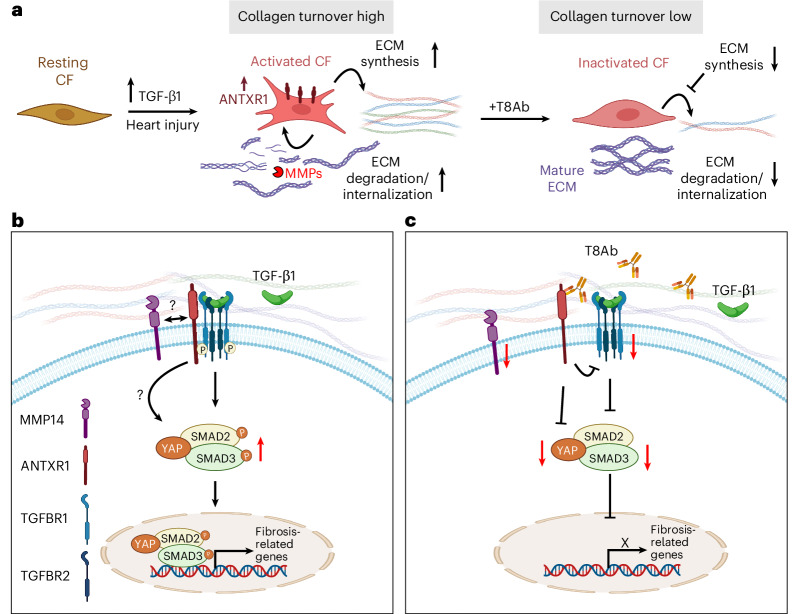
Model illustrating ANTXR1’s role in TGFβ1 driven cardiac fibrosis. **a**, ANTXR1 upregulation in activated CFs following cardiac insult enhances ECM remodeling via TGFβ pathway activation, whereas T8Abs block ECM synthesis and uptake. While not shown here, during normal physiology, or in early stages of injury before TGFβ1 has been fully activated, ANTXR1 blockade may induce ECM synthesis. **b**, ANTXR1 regulates TGFβ1 signaling by interacting with TGFBR1 during cardiac stress. **c**, T8Ab treatment disrupts the ANTXR1–TGFBR1 complex, leading to downregulated YAP and SMAD2/3 signaling. Image created with BioRender.com.

**Extended Data Fig. 10:**
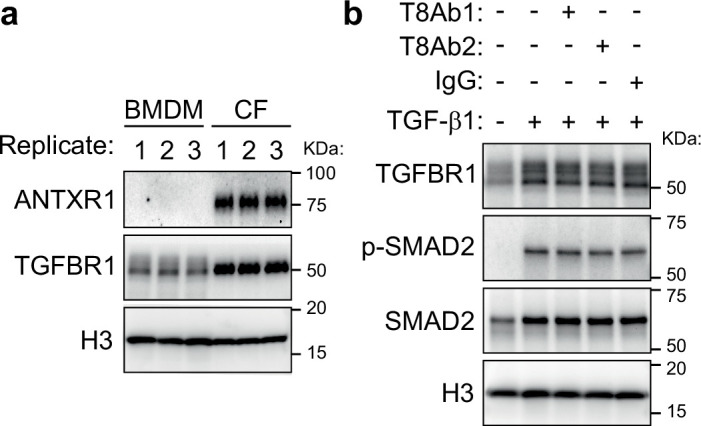
ANTXR1 neutralizing antibodies do not alter TGFβ signaling in macrophages. **a**) Immunoblotting showing endogenous ANTXR1 and TGFBR1 levels in bone marrow-derived macrophages (BMDM) and cardiac fibroblasts (CF). The BMDM replicates were derived from 3 different donor mice, while the ANTXR1 wildtype cardiac fibroblasts (CF) replicates were from three independent biological samples. **b**) Immunoblotting showing the impact of non-specific IgG, T8Ab1 or T8Ab2 treatment on TGF-β1 signaling in BMDM as assessed by SMAD2 phosphorylation (p-SMAD2). Results are representative of at least 3 independent experiments. H3 served as a loading control in (**a**) and (**b**).

While TGFβ signaling drives maladaptive collagen remodeling in CFs, TGFβ/SMAD3 signaling in macrophages has been reported to be cardioprotective^67^. *Antxr1* mRNA and protein in macrophages were undetectable (Figs. 4c and 5c and Extended Data Fig. 10a), suggesting that TGFβ signaling in this cell type may not depend on ANTXR1. As expected, T8Abs selectively antagonized TGFβ signaling in CFs but not macrophages (Fig. 7p,r and Extended Data Fig. 10b). These findings underscore the cell-type specificity of ANTXR1 targeting and highlight its potential to selectively inhibit maladaptive fibroblast signaling, while preserving protective TGFβ responses in other cell types (Fig. 8).

## Discussion

In healthy tissues, mature fibrillar collagen is highly stable, with a half-life of 80–120 days^68^. In fibrotic diseases, however, collagen turnover is dramatically increased, with a modest rise in synthesis over degradation that gradually leads to excess collagen accumulation. This net increase in collagen-rich ECM, or fibrosis, in organs such as the lung, kidney and heart has long been associated with disease, supporting the long-held view that excess ECM is inherently pathological; however, findings from genetically engineered mouse models have begun to challenge this paradigm^69^, suggesting that collagen turnover and/or alterations in matrix composition may be more critical than total collagen abundance in driving disease progression. For example, a *Col1a1* conditional KO model demonstrated a protective role for collagen in obstructive kidney injury^70^. Similarly, in cardiac disease, early collagen deposition following MI or pressure overload helps stabilize the myocardium and prevent catastrophic rupture^37,71^. In contrast, sustained remodeling of collagen at the scar–muscle border zone is considered maladaptive, leading to cardiomyocyte loss and expansion of injury^4,5^. In the present study, ANTXR1 was markedly upregulated in activated CFs following cardiac injury. By facilitating collagen turnover in response to stress, our data implicate ANTXR1 as a pathogenic driver of heart disease induced by ischemia, hypertension and obesity. ANTXR1 antagonism accelerated formation of the initial protective scar, enhanced production of cardioprotective matricellular proteins and suppressed late-stage maladaptive collagen remodeling.

In response to hypertension, the myocardium initially adapts via physiological hypertrophy (increased muscle mass), which transiently compensates for elevated blood pressure. Although physiological hypertrophy is initially reversible, prolonged stress, particularly in the context of persistent and escalating blood pressure, leads to progressive interstitial fibrosis, further increasing LV stiffness^72^. Although this newly formed ECM may help protect the cardiac wall from rupture, excessive CF expansion and ECM remodeling lead to reduced contractile function and further cardiomyocyte loss. By preventing excessive collagen turnover (a potential antecedent of cardiac dysfunction) ANTXR1 antibodies hold potential to arrest chronic damage and preserve cardiomyocyte viability.

What mechanisms underlie the functional improvements seen with ANTXR1 blockade? Previous cancer studies established that ANTXR1 is essential for collagen processing and uptake in response to stress^9^, consistent with our finding that ANTXR1 antagonism preserved intact, non-denatured collagen after injury. While a direct connection between ANTXR1 and TGFβ signaling has not previously been reported, our data demonstrate that ANTXR1 promotes canonic TGFβ–SMAD signaling, a key pathway driving collagen synthesis and maladaptive fibrosis in CFs^6^. Notably, on day 7 post-MI, ANTXR1 blockade stimulated ECM-related gene expression, including collagen and matricellular proteins, via an, as yet undefined, fibroblast-specific mechanism. While the basis of this effect requires further study, one possibility is that ANTXR1 inhibition augments ERK1/2 signaling, which was selectively elevated in CFs on MI day 7 following T8Ab treatment (Supplementary Fig. 9a). ERK1/2 activation has been shown to promote collagen production and confer cardioprotection following ischemic injury^73,74^. Furthermore, T8Ab blocked collagen degradation and promoted a matrix-preserving program characterized by *Timp1* and *Timp3* overexpression. By day 14, ANTXR1 blockade inhibited the expression of TGFβ-inducible matrix genes such as *Col1a1* and *Col1a2*, effectively halting maladaptive ECM remodeling. These findings underscore the time-sensitive effects of ANTXR1 neutralization: early augmentation of beneficial matrix formation followed by suppression of chronic pathological remodeling. Notably, previous studies have shown that hypodermic injection of decellularized ‘mature’ collagen into infarcted myocardium can exert cardioprotective effects, a procedure that has advanced to early-stage clinical trials^75,76^; however, this approach may not inhibit ongoing collagen degradation, and repeated catheter-based delivery carries a risk of procedural damage to the myocardium. In contrast, systemic delivery of ANTXR1 neutralizing antibodies may promote early matrix deposition and prevent its degradation at later stages, offering a more-controlled and less-invasive strategy to foster a protective ECM profile.

In summary, following cardiac stress, ANTXR1 protein is selectively induced in newly activated CFs, where it amplifies maladaptive collagen TGFβ signaling and promotes pathological collagen remodeling. ANTXR1 neutralization via antibody blockade enhanced early scar formation, suppressed late-stage TGF β-driven collagen turnover post-MI and improved cardiac function in multiple models of injury. While previous efforts to improve heart function by targeting TGFβ signaling have met with limited clinical success, ANTXR1 inhibition offers a unique approach: selective attenuation of collagen turnover in CFs without disrupting TGFβ-mediated cardioprotective responses in other cell types. These findings position ANTXR1 as a promising therapeutic target for the treatment of diverse forms of heart disease.

## Methods

### Experimental animal models

All mice were housed in an Association for Assessment and Accreditation of Laboratory Animal Care-accredited, pathogen-free facility, and all procedures were approved by the National Cancer Institute (NCI) Frederick Animal Care and Use Committee, in accordance with the Guide for Care and Use of Laboratory Animals (National Research Council, 2011). Mice were fed a standard autoclavable diet (5L79, LabDiet) ad libitum and maintained under conventional housing unless specified otherwise. Cardiac studies were performed on C57BL/6NCrl mice (Charles Rivers Laboratory) or *Antxr1* WT, KO or floxed mice maintained in our laboratory on an inbred C57BL/6NCrl background.

### Clinical samples

Anonymized human MI tissue samples were obtained from the Cooperative Human Tissue Network or the Duke Human Heart Repository with approval from the National Institutes of Health (NIH) Office of Human Subject Research. All protocols were IRB-approved (institutional review board Pro00005621), with informed patient consent. Sample demographics are listed in Supplementary Table 1.

### Experimental design

The study assessed the role of ANTXR1 in multiple murine models of heart disease, including MI, pressure overload, an obesity-driven HFpEF. Mice were housed under standard conditions (25 ± 2 °C, 45–55% relative humidity; 12-h light–dark cycle) with access to water and standard chow. All tests were conducted on 8–12-week-old C57BL/6NCrl mice (Charles Rivers Laboratory) or 7–18-week-old ΑΝΤXR1 KO, WT or floxed mice on the C57BL/6NCrl background^8^. Males were used for all studies unless indicated otherwise. For conditional KO studies, floxed *Antxr1* mice were crossed with tamoxifen-inducible *Col1a2-CreER* mice (The Jackson Laboratory, strain no. 029235) to create *Col1a2-cre-ER*(cre+); *Antxr1*^*+/+*^ (*Antxr1* WT), *Col1a2-cre-ER*(cre+); *Antxr1*^*+/fl*^ (*Antxr1* Het) and *Col1a2-cre-ER*(cre+); *Antxr1*^*fl//fl*^ (*Antxr1* KO) mice. The mTmG reporter strain (Jackson Laboratory, strain no. 007676) and the Col1a2-CreER strain were backcrossed more than ten generations to C57BL6-NCrl. Cre activity was induced by feeding a tamoxifen diet (400 mg tamoxifen citrate per kg diet; Inotivco cat no. TD.130860) ad libitum, starting 3 weeks before MI surgery or ATII/PE pump implantation. *Antxr1* floxed mice are available from the Jackson Laboratory (strain no. 037486).

### Myocardial infarction model

MI was induced in C57BL/6-NCr *Antxr1* WT and KO mice as described previously^77^. In brief, mice were anesthetized with 2,2,2-tribromoethanol (Millipore Sigma) in tert-amyl alcohol (Millipore Sigma) via intraperitoneal injection (0.2 ml per 10 g body weight). The surgical area was shaved and sterilized. After direct visualization through a neck incision, mice were intubated and ventilated (tidal volume, 1.0 ml; rate, 110 bpm; R405 Mouse Ventilator, RWD). A left thoracotomy exposed the heart through the fourth intercostal space and the left anterior descending coronary artery was ligated with 8–0 nylon suture (DemeTECH, NL146800,65F0P). The chest was closed and animals weaned from ventilation to avoid pneumothorax. After surgery, the animal cages were placed on a heating pad (37–39 °C) for at least 7 days. Left ventricular function was assessed via echocardiography on the days indicated as described below. T8Ab or PBS (control) was administered intraperitoneally three times weekly, beginning day 1 post-surgery.

### Hypertension model

Hypertension was induced by subcutaneous implantation of a 28-day osmotic minipump (Alzet, Model 2004) delivering angiotensin II (1.5 μg g^−1 ^d^−1^; ≥93% pure, Sigma-Aldrich) and phenylephrine (50 μg g^−1 ^d^−1^; Sigma-Aldrich) as previously described^78,79^. Control animals received 0.9% NaCl. For prevention studies, mice were treated with 15 mg kg^−1^ of T8Ab or PBS control via intraperitoneal injection three times weekly starting on day 1 post-implantation. To assess T8Ab therapeutic efficacy post-injury, minipumps were filled with higher-purity angiotensin II (≥98%, BOC Sciences) and phenylephrine (Sigma-Aldrich) at the same doses. Once the left ventricular EF dropped from ~80% to ~40% (day 7), mice received 15 mg kg^−1^ T8Ab or PBS control three times weekly. EF was monitored by echocardiography. On day 28, mice were killed and hearts were collected for analysis.

### Mouse model of heart failure with preserved ejection fraction

HFpEF was induced as previously described^3^. Briefly, mice were given ad libitum access to either a standard diet (cat. no. 2916, Teklad) or HFD (D12492, Research Diet) for the duration of the study. L-NAME (0.5 g l^−1^, Sigma-Aldrich) was added to the drinking water of the HFD group. Water was adjusted to pH to 7.4 before administration.

### Echocardiography and doppler imaging

Transthoracic echocardiography was performed using a Fujifilm VisualSonics Vevo2100 Ultrasound System equipped with an MS400 (18–38 MHz) transducer. Mice were initially anesthetized with 3% isoflurane (Covetrus) and maintained at 1–1.5% during image acquisition. To assess systolic function short-axis B-mode images were used initially followed by long-axis B-mode images in later studies; apical four-chamber views were used for diastolic function and long-axis B-mode views for peak longitudinal strain rate. All measurements were analyzed using Vevo Lab 2100 software.

### Pulse wave velocity

Pulse wave velocity was measured noninvasively by recording pulse waves at the ascending and abdominal aorta. The distance (Δ*D*) between these points was measured using the platform meter, and the transit time (Δ*T*) was calculated using electrocardiography-based waveform analysis. Pulse wave velocity was calculated as Δ*D*/Δ*T*.

### Exercise exhaustion test

Mice were acclimated to treadmill running for three consecutive days (20 min d^−1^), followed by two rest days. The exhaustion test was conducted on a treadmill (IITC Life Science Model 800) at a 20° incline. Mice ran at 5 m min^−1^ for 4 min, followed by an increase to 14 m min^−1^ until exhaustion, defined as spending ten continuous seconds in the fatigue zone in contact with the electric grid. Running time was recorded at the end point.

### Glucose-tolerance test

Glucose-tolerance tests were conducted as previously described^80^. In brief, mice were fasted for 6 h beginning at 6:00, followed by oral gavage of 2 g kg^−1^ glucose. Tail vein blood was collected at 0, 15, 30, 60 and 120 min using microhematocrit tubes (Thermo Fisher, cat. no. 22-274913), and glucose levels were measured with an Alphatrak2 glucometer (Zoetis). The area under the curve was calculated using GraphPad Prism v.10.

### Single-cell preparation from mouse hearts and sequencing

Mice were anesthetized (Avertin, 0.2 ml per 10 g) and hearts were perfused with ice-cold sterile PBS (~1 ml min^−1^ per heart for 10 min) using a peristaltic pump (Miniplus, GILSON) to remove blood cells^23^. Cardiac tissue was dissociated using the Multi Tissue Dissociation kit 2 (Miltenyi Biotec, cat. no. 130-110-203), which enriches for noncardiomyocyte populations. Viability exceeded ~90% across all samples. Library preparation and sequencing were performed at the Frederick National Laboratory for Cancer Research Sequencing Facility. For the AngII/PE model, ~16,500 cells per sample were processed using Chromium Next GEM Single Cell 3ʹ Reagent Kits v.3.1; for the MI model, 5ʹ Reagent kits v.2 with dual indices were used. Libraries were sequenced on a NovaSeq 6000. AngII/PE samples were sequenced using a 28 + 90-cycle nonsymmetric run, yielding >514 million reads per sample; MI samples used a 151 + 151-cycle symmetric run, yielding >424 million reads per sample. Barcode demultiplexing allowed for one mismatch. Quality scores revealed that over 95.0% of bases in the barcode regions have Q30 or above, at least 91% of bases in the read have Q30 or above and more than 95% of bases in the unique molecular identifier have Q30 or above.

### Analysis of the scRNA-seq dataset

FASTQ files were aligned to the mouse genome (refdata-gex-mm10-2020-A) using Cell Ranger (v.7.0.0, 10x Genomics) with the default parameters on the NIH HPC Biowulf cluster (http://hpc.nih.gov). Output h5 files were analyzed using Seurat (v.4.1.1)^81^ according to the manual. Filtering criteria included: min.cells ≥ 10, min.features ≥ 250, nCount_RNA ≥ 500, nFeature_RNA ≤ 10,000 and percent.mt ≤ 10. Doublets were removed using DoubletFinder (v.2.0.3)^82^. Differential expression analysis was performed using pseudo-bulk RNA-seq with DESeq2 (v.1.36.0)^83^. GO enrichment analysis used clusterProfiler (v.4.4.4)^84^. Visualization included volcano plots (ggplot2 v.3.4.3) and heat maps (ComplexHeatmap v.2.15.1). RNA velocity analysis was conducted with Velocyto v.0.17 (ref. ^85^) and scvelo (v.0.2.4)^86^.

### Cell culture and immortalization of mouse cardiac fibroblasts

CHO-PR230 (CHO) cells, a kind gift from S.H. Leppla (NIAID), were cultured in Hams F-12 medium with 10% FBS. Primary CFs were purified from a *tsA58*^*+*^/*Antxr1*^*flox/flox*^ mice (generated by crossing JAX strain no. 032619 (Immortomouse) and 037486 (B6N.Cg-*Antxr1*^*tm1.1Bstc*^/J)) on a C57BL/6-NCr background. Hearts were dissociated using the Multi Tissue Dissociation kit 2 (Miltenyi) and fibroblasts purified as previously described^87^. In brief, cells were sequentially negatively selected using anti-CD45 (Miltenyi Biotec, cat. no. 130-052-301) and anti-CD31 (Miltenyi Biotec, cat. no. 130-097-418) microbeads followed by positive selection with an anti-fibroblast antibody (Miltenyi Biotec, clone mEF-SK4, cat. no. 130-120-802). CFs were cultured on 0.1% gelatin-coated T75 flasks (STEMCELL, cat. no. 07903) in DMEM/F-12 (Thermo Fisher, cat. no. 11330032) supplemented with 10% FBS (Avantor Seradigm, cat. no. 97068-085), sodium pyruvate (Thermo Fisher, cat. no. 11360070), MEM non-essential amino acids (Thermo Fisher, cat. no. 11140050), penicillin–streptomycin (Corning, cat. no. 30-002-CI) and IFNγ (PeproTech, 5 ng ml^−1^, cat. no. 315-05) at 32 °C (for immortalization) with 5% CO_2_. After two passages, cells were cryopreserved. For experiments, cells (≤10 passages) were cultured at 37 °C in IFNγ-free medium, then starved in 0.5% FBS for 16 h before stimulation with TGF-β (10 ng ml^−1^). Where indicated, T8Ab (10 μg ml^−1^) or MG132 (10 μM; Selleckchem, cat. no. S2619) was added.

### Culture of human cardiac fibroblasts

Primary human ventricular fibroblasts (NHCF-V; Lonza, cat. no. CC-2904) were cultured in 0.1% gelatin-coated T75 flasks using DMEM/F-12 supplemented with 10% FBS, sodium pyruvate, non-essential amino acids and penicillin–streptomycin at 37 °C with 5% CO_2_. After one passage, cells were seeded in 6-cm dishes (2.5 × 10_4_ cells per ml; 6 ml per dish), starved for 16 h in 0.5% FBS, then treated with TGF-β (10 ng ml^−1^, PeproTech, cat. no. 100-21C) ± T8Ab for 1 h before lysis for immunoblotting.

### Gel contraction assay

The gel contraction assay was performed as previously described^41^. In brief, mouse CFs were plated at 5 × 10⁵ cells per flask in DMEM/F-12 with 10% FBS, pyruvate, non-essential amino acids and pen–strep. After 1 day the medium was changed to basic DMEM/F-12 medium containing 0.5% FBS (LS medium) for 16 h. Cells were then trypsinized, collected in LS medium, centrifuged, resuspended in fresh LS medium and kept on ice before adding ice-cold PureCol EZ Gel (Neutralized Type I Collagen Solution, ~5 mg ml^−1^, Advanced BioMatrix, 5074) to prepare a cell/gel mixture with 2.5 × 10^5^ cells per ml and 1 mg ml^−1^ collagen gel. TGF-β (10 ng ml^−1^) ± T8Ab (20 μg ml^−1^) was added before plating 500 μl of the mixture per well (1.25 × 10^5^ cells per well) into an untreated 24-well plate and allowing the cells/gel mixture to solidify at 37 °C, 5% CO_2_. After 2 h at 37 °C for gelation, 500 μl of medium was added per well and gels were detached. After 48 h further incubation at 37 °C, gel images were acquired (Zeiss SteREO Discovery v.20) and contraction was calculated using Fiji (v.2.14.0) as:

$$\mathrm{Contraction}\,( \% )=1-(\mathrm{gel}\,\mathrm{area}/\mathrm{total}\,\mathrm{well}\,\mathrm{area}).$$

### RT–qPCR assay

RNA was extracted using the RNeasy Mini kit (QIAGEN, 74104) and complementary DNA was synthesized from 1 μg RNA using the RevertAid First Strand cDNA kit (Thermo, K1621). RT–qPCR was performed with iTaq Universal SYBR Green Supermix (Bio-Rad, 1725120). Primer sequences are listed in Supplementary Table 13.

### Cell lysates, coimmunoprecipitation and immunoblotting analysis

Cells were rinsed twice with cold PBS and lysed in RIPA buffer (Millipore, cat. no. 20-188) with protease/phosphatase inhibitors. Lysates were sonicated, centrifuged (20,000*g*, 10 min, 4 °C), and denatured in Laemmli sample buffer. For co-IP, cells were rinsed with ice-cold PBS, lysed with buffer (25 mM Tris, pH 7.5, 75 mM NaCl, 0.5% Triton X-100 and 10% glycerol, with inhibitors (Thermo Fisher, cat. no. 78443), incubated on ice for 40 min, centrifuged and quantified (Pierce BCA kit, Thermo, cat. no. 23225). One mg of protein was pre-cleared with 10 μl Dynabeads Protein G (Thermo, cat. no. 10003D) for 1 h at 4 °C, then incubated overnight with 1 μg antibody. After adding 10 μl Dynabeads, samples were rotated for 2 h, washed 5× with lysis buffer and eluted in Laemmli buffer (Bio-Rad, cat. no. 1610747). Samples were boiled for 10 min before SDS–PAGE.

### Proximity ligation assay

Mouse CFs (3.5 × 10³ per well) were plated in eight-well cell culture slides (MatTek, CCS-8) coated with poly-D-lysine (Advanced BioMatrix, 5049) in DMEM/F-12 + 10% FBS, sodium pyruvate and non-essential amino acids. After 24 h, cells were serum-starved (0.5% FBS) for 16 h, then stimulated with TGF-β (10 ng ml^−1^) for 1 h. Slides were placed on ice and the proximity ligation assay was performed as per the manufacturer’s protocol^88^. Reagent details are listed in Supplementary Table 14.

### Histology and immunofluorescence staining

Mouse hearts were collected and fixed overnight in 10% formalin, dehydrated and paraffin embedded. Sections (5 μm) were stained using the Picro Sirius Red Stain kit (Abcam, cat. no. ab150681) to visualize collagen fibers, per manufacturer instructions. For IF staining, hearts were rinsed, infused with OCT, embedded and cryopreserved. Frozen 5-μm sections were cut onto Superfrost Plus slides (Fisher, cat. no. 12-550-15) and fixed with 1% paraformaldehyde (PFA) for 20 min. Sections were blocked and permeabilized for 1 h at room temperature in 1% blocking reagent (Roche, cat. no. 11096176001) with 0.1% Triton X-100. Samples were incubated with c37 anti-rabbit ANTXR1 antibody (Abcam, cat. no. ab241067; 1:100 dilution) for 2 h at room temperature, followed by secondary staining with FITC-labeled anti-rabbit (Jackson ImmunoResearch, cat. no. 111-095-144) and 488-conjugated goat anti-FITC (Thermo Fisher, cat. no. A11055). IF of denatured collagen with biotin-CHP (3Helix, cat. no. BIO300) was performed as previously described^89^ and detected using Texas red-streptavidin. Cardiomyocytes were stained with Alexa Fluor 647-conjugated anti-cardiac troponin T (BD Pharmingen) for 30 min at room temperature. Slides were mounted using Vectashield PLUS Antifade Mounting Medium with 4,6-diamidino-2-phenylindole (DAPI). Images were acquired on a Leica DMi8 microscope with a Yokogawa CSU-W1 spinning disk confocal scanner and Andor Zyla 4.2 sCMOS camera, using a ×40 NA 1.4 oil immersion lens. Deconvolution was performed using Andor Fusion software^90^. For ANTXR1 and TGFBR1 co-staining, stimulated CFs were cooled on ice for 10 min, incubated with m830 anti-ANTXR1 antibody (1 mg ml^−1^; 1:100 dilution)^11^ for 1 h, then washed in cold culture medium and PBS. Cells were fixed with 1% PFA (10 min on ice, 10 min at room temperature), blocked/permeabilized with 1% blocking reagent + 0.1% Triton X-100 for 10 min, then incubated with anti-TGFBR1 (1:100 dilution) for 1 h. For YAP and SMAD2/3 staining, 0.2% Triton X-100 was used. Quantification was performed in Fiji (v.2.14.0).

### ANTXR1 KO and rescue in mouse cardiac fibroblasts

CFs isolated from tsA58^+^/*Antxr1*^*flox/flox*^ mice were transduced with Adenoviral-cre (Ad-Cre-IRES-GFP, cat. no. 1710, Vector Biolabs) to induce *Antxr1* knockout. Two days later, GFP⁺ cells were sorted via flow cytometry, and ANTXR1 deletion was confirmed by western blot. Ad-GFP (Vector Biolabs, cat. no. 1060) was used as a negative control. For rescue experiments, full-length human ANTXR1 and a cytosolic domain deletion mutant (Δ355–564 aa, ANTXR1-CytDel) were cloned into pLenti vector (Addgene, cat. no. 17448). Lentivirus particles were generated for each construct and used to transduce ANTXR1 KO fibroblasts alongside empty vector controls. Stable lines were selected with puromycin (6 μg ml^−1^).

### Flow cytometry assay

Cells were trypsinized and resuspended in 200 μl cold PBS with 0.5% BSA (P-BSA). Primary antibodies (1 μg per 2 × 10⁶ cells) were incubated for 1 h on ice. Cells were washed three times in P-BSA, incubated with fluorophore-conjugated secondary antibodies for 30 min on ice, washed again, and analyzed on a BD LSRFortessa. Data were processed using FlowJo (v.10.8.1).

### Bone marrow-derived macrophage assay

Bone marrow cells were isolated from C57BL/6 mice^91^ by flushing femurs with cold PBS, followed by ACK lysis buffer treatment (Gibco, cat. no. A1049201) to remove red blood cells. Cells were cultured in DMEM/F-12 + 10% FBS, pyruvate, non-essential amino acids, pen–strep and 10 ng ml^−1^ M-CSF (PeproTech, cat. no. 315-02) in six-well plates (6 × 10⁶ cells per well, 5 ml). Medium was changed every 3 days. On day 6, cells were plated at 1.5 × 10⁶ per dish in 6-cm dishes and stimulated with TGF-β (± anti-ANTXR1 antibody), as described for fibroblasts. After 1 h, cells were collected for western blot analysis.

### Isolation of T8Ab2

T8Ab2 (clone YL9) was generated as previously described^92^. In brief, a naive human single-chain variable fragment (scFv) phage display library comprising approximately 10¹¹ unique clones (derived from the B cells of 58 healthy donors) was screened against the extracellular domain of human ANTXR1. After three rounds of panning, monoclonal phage ELISA identified several candidate binders, which were subsequently evaluated for specificity by flow cytometry, Biacore surface plasmon resonance and functional assays. YL9, one of the lead clones with high affinity, stability and the ability to block collagen uptake, was reformatted into a full-length IgG and used in the studies described herein. A comprehensive characterization of this antibody will be reported separately.

### Prediction of protein crystal structure and protein interaction

Structures of the anti-ANTXR1 antibodies T8Ab1 (clone L2) and T8Ab2 (clone YL9) were modeled using ABodyBuilder2 (ref. ^72^). The extracellular domain of ANTXR1 was obtained from the Protein Data Bank (ID 3N2N)^93,94^. Antibody-ECD docking was performed with HADDOCK2.4 using default settings^95,96^. The predicted complex with the highest HADDOCK score of each conformation was selected and the structural graphics were analyzed and visualized in PyMOL (v.2.5.0; Schrödinger). The top-scoring complexes were visualized and analyzed in PyMOL (v.2.5.0; Schrödinger).

### Quantification and statistical analysis

Statistical analyses were performed using GraphPad Prism v.10.2.0. Two-group comparisons used unpaired two-tailed Student’s *t*-tests (or two-tailed Wilcoxon tests for violin plots). For three or more groups, a one-way analysis of variance with Tukey’s post hoc test was used. Kaplan–Meier survival was analyzed by a log-rank (Mantel–Cox) test. Data are presented as mean ± s.e.m. or mean ± s.d., as indicated. Echocardiography data were collected and analyzed blinded to group. Occasional data points were excluded due to technical artifacts (for example, an image out of focus) and statistical outliers were removed. Sample sizes (*n*) are noted in the figure legends. A *P* value < 0.05 was considered statistically significant.

### Reporting summary

Further information on research design is available in the Nature Portfolio Reporting Summary linked to this article.

## Supplementary information

Supplementary Information Supplementary Figs. 1–22, Full immunoblot images for Supplementary Figs. 17b and 18.

Reporting Summary

Supplementary Tables Supplementary Tables 1–14.

## Source data

Source Data Fig. 1 Unprocessed western blots.

Source Data Fig. 1 Statistical source data.

Source Data Fig. 2 Statistical source data.

Source Data Fig. 3 Statistical source data.

Source Data Fig. 4 Statistical source data.

Source Data Fig. 5 Statistical source data.

Source Data Fig. 6 Statistical source data.

Source Data Fig. 7 Unprocessed western blots.

Source Data Fig. 7 Statistical source data.

Source Data Extended Data Fig. 2 Statistical source data.

Source Data Extended Data Fig. 3 Statistical source data.

Source Data Extended Data Fig. 4 Statistical source data.

Source Data Extended Data Fig. 5 Statistical source data.

Source Data Extended Data Fig. 6 Statistical source data.

Source Data Extended Data Fig. 7 Unprocessed western blots.

Source Data Extended Data Fig. 7b Statistical source data.

Source Data Extended Data Fig. 8 Unprocessed western blots.

Source Data Extended Data Fig. 8d Statistical source data.

Source Data Extended Data Fig. 9 Unprocessed western blots.

Source Data Extended Data Fig. 10 Unprocessed western blots.

## Data Availability

All data are available in the main text or the Supplementary Information. The single-cell RNA-seq data have been deposited in the Gene Expression Omnibus under accession no. GSE266597. Public datasets from GSE183852 and the Broad Institute’s Single Cell Portal (SCP1303) were used for Fig. 1c (https://singlecell.broadinstitute.org/single_cell/study/SCP1303/).

## References

[1] Roth, G. A. et al. Global burden of cardiovascular diseases and risk factors, 1990-2019: update from the GBD 2019 study. *J. Am. Coll. Cardiol.* **76**, 2982–3021 (2020). 33309175 10.1016/j.jacc.2020.11.010 PMC7755038

[2] Vaduganathan, M., Mensah, G. A., Turco, J. V., Fuster, V. & Roth, G. A. The global burden of cardiovascular diseases and risk: a compass for future health. *J. Am. Coll. Cardiol.* **80**, 2361–2371 (2022). 36368511 10.1016/j.jacc.2022.11.005

[3] Schiattarella, G. G. et al. Nitrosative stress drives heart failure with preserved ejection fraction. *Nature* **568**, 351–356 (2019). 30971818 10.1038/s41586-019-1100-z PMC6635957

[4] Frangogiannis, N. G. Cardiac fibrosis. *Cardiovasc Res.* **117**, 1450–1488 (2021). 33135058 10.1093/cvr/cvaa324 PMC8152700

[5] Travers, J. G., Kamal, F. A., Robbins, J., Yutzey, K. E. & Blaxall, B. C. Cardiac fibrosis: the fibroblast awakens. *Circ. Res.* **118**, 1021–1040 (2016). 26987915 10.1161/CIRCRESAHA.115.306565 PMC4800485

[6] Frangogiannis, N. G. Transforming growth factor-β in myocardial disease. *Nat. Rev. Cardiol.* **19**, 435–455 (2022). 34983937 10.1038/s41569-021-00646-w

[7] Lopez, B. et al. Diffuse myocardial fibrosis: mechanisms, diagnosis and therapeutic approaches. *Nat. Rev. Cardiol.* **18**, 479–498 (2021). 33568808 10.1038/s41569-020-00504-1

[8] Cullen, M. et al. Host-derived tumor endothelial marker 8 promotes the growth of melanoma. *Cancer Res.* **69**, 6021–6026 (2009). 19622764 10.1158/0008-5472.CAN-09-1086 PMC2721800

[9] Hsu, K. S. et al. Cancer cell survival depends on collagen uptake into tumor-associated stroma. *Nat. Commun.* **13**, 7078 (2022). 36400786 10.1038/s41467-022-34643-5 PMC9674701

[10] St Croix, B. et al. Genes expressed in human tumor endothelium. *Science* **289**, 1197–1202 (2000). 10947988 10.1126/science.289.5482.1197

[11] Szot, C. et al. Tumor stroma-targeted antibody-drug conjugate triggers localized anticancer drug release. *J. Clin. Invest.* **128**, 2927–2943 (2018). 29863500 10.1172/JCI120481 PMC6025988

[12] Nanda, A. et al. TEM8 interacts with the cleaved C5 domain of collagen α 3(VI). *Cancer Res.* **64**, 817–820 (2004). 14871805 10.1158/0008-5472.can-03-2408

[13] Chaudhary, A. et al. TEM8/ANTXR1 blockade inhibits pathological angiogenesis and potentiates tumoricidal responses against multiple cancer types. *Cancer Cell* **21**, 212–226 (2012). 22340594 10.1016/j.ccr.2012.01.004 PMC3289547

[14] Andersen, N. J. et al. Anthrax toxin receptor 1 is essential for arteriogenesis in a mouse model of hindlimb ischemia. *PLoS ONE* **11**, e0146586 (2016). 26785120 10.1371/journal.pone.0146586 PMC4718698

[15] Stranecky, V. et al. Mutations in ANTXR1 cause GAPO syndrome. *Am. J. Hum. Genet.* **92**, 792–799 (2013). 23602711 10.1016/j.ajhg.2013.03.023 PMC3644626

[16] Besschetnova, T. Y. et al. Regulatory mechanisms of anthrax toxin receptor 1-dependent vascular and connective tissue homeostasis. *Matrix Biol.* **42**, 56–73 (2015). 25572963 10.1016/j.matbio.2014.12.002 PMC4409530

[17] Koenig, A. L. et al. Single-cell transcriptomics reveals cell-type-specific diversification in human heart failure. *Nat. Cardiovasc. Res.* **1**, 263–280 (2022). 35959412 10.1038/s44161-022-00028-6 PMC9364913

[18] Chaffin, M. et al. Single-nucleus profiling of human dilated and hypertrophic cardiomyopathy. *Nature* **608**, 174–180 (2022). 35732739 10.1038/s41586-022-04817-8 PMC12591363

[19] Patten, R. D. Models of gender differences in cardiovascular disease. *Drug Discov. Today Dis. Models* **4**, 227–232 (2007). 19081826 10.1016/j.ddmod.2007.11.002 PMC2597869

[20] American Heart Association. Heart failure. https://www.heart.org/en/health-topics/heart-failure.

[21] Boixel, C. et al. Fibrosis of the left atria during progression of heart failure is associated with increased matrix metalloproteinases in the rat. *J. Am. Coll. Cardiol.* **42**, 336–344 (2003). 12875773 10.1016/s0735-1097(03)00578-3

[22] Lo, M. et al. Effector-attenuating substitutions that maintain antibody stability and reduce toxicity in mice. *J. Biol. Chem.* **292**, 3900–3908 (2017). 28077575 10.1074/jbc.M116.767749 PMC5339770

[23] Feng, Y. et al. Engineering CD276/B7-H3-targeted antibody-drug conjugates with enhanced cancer-eradicating capability. *Cell Rep.* **42**, 113503 (2023). 38019654 10.1016/j.celrep.2023.113503 PMC10872261

[24] McLellan, M. A. et al. High-resolution transcriptomic profiling of the heart during chronic stress reveals cellular drivers of cardiac fibrosis and hypertrophy. *Circulation* **142**, 1448–1463 (2020). 32795101 10.1161/CIRCULATIONAHA.119.045115 PMC7547893

[25] Schafer, S. et al. IL-11 is a crucial determinant of cardiovascular fibrosis. *Nature* **552**, 110–115 (2017). 29160304 10.1038/nature24676 PMC5807082

[26] Jeong, D. et al. Matricellular protein CCN5 reverses established cardiac fibrosis. *J. Am. Coll. Cardiol.* **67**, 1556–1568 (2016). 27150688 10.1016/j.jacc.2016.01.030 PMC5887128

[27] Ruiz-Villalba, A. et al. Single-cell RNA sequencing analysis reveals a crucial role for CTHRC1 (collagen triple helix repeat containing 1) cardiac fibroblasts after myocardial infarction. *Circulation* **142**, 1831–1847 (2020). 32972203 10.1161/CIRCULATIONAHA.119.044557 PMC7730974

[28] Huang, Y. et al. Deficiency of cartilage oligomeric matrix protein causes dilated cardiomyopathy. *Basic Res. Cardiol.* **108**, 374 (2013). 23917519 10.1007/s00395-013-0374-9

[29] Palao, T. et al. Thrombospondin-4 knockout in hypertension protects small-artery endothelial function but induces aortic aneurysms. *Am. J. Physiol. Heart Circ. Physiol.* **310**, H1486–H1493 (2016). 26968543 10.1152/ajpheart.00046.2016

[30] Thavapalachandran, S. et al. Platelet-derived growth factor-AB improves scar mechanics and vascularity after myocardial infarction. *Sci. Transl. Med*. **12** (2020). 10.1126/scitranslmed.aay2140 31894101

[31] Eckhouse, S. R. et al. Local hydrogel release of recombinant TIMP-3 attenuates adverse left ventricular remodeling after experimental myocardial infarction. *Sci. Transl. Med.* **6**, 223ra221 (2014). 10.1126/scitranslmed.3007244 PMC4365799 24523321

[32] Kandalam, V. et al. Early activation of matrix metalloproteinases underlies the exacerbated systolic and diastolic dysfunction in mice lacking TIMP3 following myocardial infarction. *Am. J. Physiol. Heart Circ. Physiol.* **299**, H1012–H1023 (2010). 20675565 10.1152/ajpheart.00246.2010 PMC4116393

[33] Kim, J. et al. Cytokine-like 1 regulates cardiac fibrosis via modulation of TGF-β signaling. *PLoS ONE* **11**, e0166480 (2016). 27835665 10.1371/journal.pone.0166480 PMC5105950

[34] Kuwabara, J. T. et al. Consequences of PDGFRα(+) fibroblast reduction in adult murine hearts. *Elife*10.7554/eLife.69854 (2022). 10.7554/eLife.69854 PMC9576271 36149056

[35] Rui, H., Zhao, F., Yuhua, L. & Hong, J. Suppression of SMOC2 alleviates myocardial fibrosis via the ILK/p38 pathway. *Front. Cardiovasc. Med.* **9**, 951704 (2022). 36935650 10.3389/fcvm.2022.951704 PMC10017443

[36] Shimazaki, M. et al. Periostin is essential for cardiac healing after acute myocardial infarction. *J. Exp. Med.* **205**, 295–303 (2008). 18208976 10.1084/jem.20071297 PMC2271007

[37] Oka, T. et al. Genetic manipulation of periostin expression reveals a role in cardiac hypertrophy and ventricular remodeling. *Circ. Res.* **101**, 313–321 (2007). 17569887 10.1161/CIRCRESAHA.107.149047 PMC2680305

[38] Wang, B. et al. The Kruppel-like factor KLF15 inhibits connective tissue growth factor (CTGF) expression in cardiac fibroblasts. *J. Mol. Cell. Cardiol.* **45**, 193–197 (2008). 18586263 10.1016/j.yjmcc.2008.05.005 PMC2566509

[39] Oliver, P. M. et al. Hypertension, cardiac hypertrophy, and sudden death in mice lacking natriuretic peptide receptor A. *Proc. Natl Acad. Sci. USA* **94**, 14730–14735 (1997). 9405681 10.1073/pnas.94.26.14730 PMC25105

[40] Iwasaki, H. et al. PlGF repairs myocardial ischemia through mechanisms of angiogenesis, cardioprotection and recruitment of myo-angiogenic competent marrow progenitors. *PLoS ONE* **6**, e24872 (2011). 21969865 10.1371/journal.pone.0024872 PMC3182165

[41] Alexanian, M. et al. A transcriptional switch governs fibroblast activation in heart disease. *Nature* **595**, 438–443 (2021). 34163071 10.1038/s41586-021-03674-1 PMC8341289

[42] Zeisberg, E. M. et al. Endothelial-to-mesenchymal transition contributes to cardiac fibrosis. *Nat. Med.* **13**, 952–961 (2007). 17660828 10.1038/nm1613

[43] Okayama, K. et al. Hepatocyte growth factor reduces cardiac fibrosis by inhibiting endothelial-mesenchymal transition. *Hypertension* **59**, 958–965 (2012). 22392903 10.1161/HYPERTENSIONAHA.111.183905

[44] Takawale, A. et al. Myocardial recovery from ischemia-reperfusion is compromised in the absence of tissue inhibitor of metalloproteinase 4. *Circ. Heart. Fail.* **7**, 652–662 (2014). 24842912 10.1161/CIRCHEARTFAILURE.114.001113

[45] Dai, Z. et al. Loss of endothelial hypoxia inducible factor-prolyl hydroxylase 2 induces cardiac hypertrophy and fibrosis. *J. Am. Heart Assoc.* **10**, e022077 (2021). 34743552 10.1161/JAHA.121.022077 PMC8751916

[46] Tirronen, A. et al. The ablation of VEGFR-1 signaling promotes pressure overload-induced cardiac dysfunction and sudden death. *Biomolecules*10.3390/biom11030452 (2021). 10.3390/biom11030452 PMC8002705 33802976

[47] Ullah, K., Ai, L., Humayun, Z. & Wu, R. Targeting endothelial HIF2α/ARNT expression for ischemic heart disease therapy. *Biology*10.3390/biology12070995 (2023). 10.3390/biology12070995 PMC10376750 37508425

[48] Takawale, A. et al. Myocardial overexpression of TIMP3 after myocardial infarction exerts beneficial effects by promoting angiogenesis and suppressing early proteolysis. *Am. J. Physiol. Heart Circ. Physiol.* **313**, H224–H236 (2017). 28550172 10.1152/ajpheart.00108.2017

[49] Lu, G. et al. BMP6 knockdown enhances cardiac fibrosis in a mouse myocardial infarction model by upregulating AP-1/CEMIP expression. *Clin. Transl. Med.* **13**, e1296 (2023). 37313693 10.1002/ctm2.1296 PMC10265437

[50] Wright, L. H., Herr, D. J., Brown, S. S., Kasiganesan, H. & Menick, D. R. Angiokine Wisp-1 is increased in myocardial infarction and regulates cardiac endothelial signaling. *JCI Insight*10.1172/jci.insight.95824 (2018). 10.1172/jci.insight.95824 PMC5916251 29467324

[51] Gravning, J., Ahmed, M. S., von Lueder, T. G., Edvardsen, T. & Attramadal, H. CCN2/CTGF attenuates myocardial hypertrophy and cardiac dysfunction upon chronic pressure-overload. *Int. J. Cardiol.* **168**, 2049–2056 (2013). 23452880 10.1016/j.ijcard.2013.01.165

[52] Dorn, L. E., Petrosino, J. M., Wright, P. & Accornero, F. CTGF/CCN2 is an autocrine regulator of cardiac fibrosis. *J. Mol. Cell. Cardiol.* **121**, 205–211 (2018). 30040954 10.1016/j.yjmcc.2018.07.130 PMC6260782

[53] Flinn, M. A. et al. Myofibroblast Ccn3 is regulated by Yap and Wwtr1 and contributes to adverse cardiac outcomes. *Front. Cardiovasc. Med.* **10**, 1142612 (2023). 36998974 10.3389/fcvm.2023.1142612 PMC10043314

[54] Zha, Y. et al. ADAMTS8 promotes cardiac fibrosis partly through activating EGFR dependent pathway. *Front. Cardiovasc. Med.* **9**, 797137 (2022). 35224040 10.3389/fcvm.2022.797137 PMC8866452

[55] Sun, Y. et al. Inhibition of Fap promotes cardiac repair by stabilizing BNP. *Circ. Res.* **132**, 586–600 (2023). 36756875 10.1161/CIRCRESAHA.122.320781

[56] Khalil, H. et al. Fibroblast-specific TGF-β-Smad2/3 signaling underlies cardiac fibrosis. *J. Clin. Invest.* **127**, 3770–3783 (2017). 28891814 10.1172/JCI94753 PMC5617658

[57] Davis, J. & Molkentin, J. D. Myofibroblasts: trust your heart and let fate decide. *J. Mol. Cell. Cardiol.* **70**, 9–18 (2014). 24189039 10.1016/j.yjmcc.2013.10.019 PMC3995855

[58] Younesi, F. S., Miller, A. E., Barker, T. H., Rossi, F. M. V. & Hinz, B. Fibroblast and myofibroblast activation in normal tissue repair and fibrosis. *Nat. Rev. Mol. Cell Biol.*10.1038/s41580-024-00716-0 (2024). 38589640 10.1038/s41580-024-00716-0

[59] Garoffolo, G. et al. Reduction of cardiac fibrosis by interference with YAP-dependent transactivation. *Circ. Res.* **131**, 239–257 (2022). 35770662 10.1161/CIRCRESAHA.121.319373

[60] Lijnen, P., Petrov, V., Rumilla, K. & Fagard, R. Transforming growth factor-β 1 promotes contraction of collagen gel by cardiac fibroblasts through their differentiation into myofibroblasts. *Methods Find. Exp. Clin. Pharmacol.* **25**, 79–86 (2003). 12731452 10.1358/mf.2003.25.2.723680

[61] Koenig, G. C. et al. MT1-MMP-dependent remodeling of cardiac extracellular matrix structure and function following myocardial infarction. *Am. J. Pathol.* **180**, 1863–1878 (2012). 22464947 10.1016/j.ajpath.2012.01.022 PMC3349831

[62] Hanna, A. et al. Collagen denaturation in the infarcted myocardium involves temporally distinct effects of MT1-MMP-dependent proteolysis and mechanical tension. *Matrix Biol.* **99**, 18–42 (2021). 34048934 10.1016/j.matbio.2021.05.005 PMC8591556

[63] Hiemer, S. E., Szymaniak, A. D. & Varelas, X. The transcriptional regulators TAZ and YAP direct transforming growth factor β-induced tumorigenic phenotypes in breast cancer cells. *J. Biol. Chem.* **289**, 13461–13474 (2014). 24648515 10.1074/jbc.M113.529115 PMC4036353

[64] Liu, F. et al. Mechanosignaling through YAP and TAZ drives fibroblast activation and fibrosis. *Am. J. Physiol. Lung Cell. Mol. Physiol.* **308**, L344–L357 (2015). 25502501 10.1152/ajplung.00300.2014 PMC4329470

[65] Francisco, J. et al. Blockade of fibroblast YAP attenuates cardiac fibrosis and dysfunction through MRTF-A inhibition. *JACC Basic Transl. Sci.* **5**, 931–945 (2020). 33015415 10.1016/j.jacbts.2020.07.009 PMC7524792

[66] Mia, M. M. et al. Loss of Yap/Taz in cardiac fibroblasts attenuates adverse remodelling and improves cardiac function. *Cardiovasc. Res.* **118**, 1785–1804 (2022). 34132780 10.1093/cvr/cvab205

[67] Chen, B. et al. Macrophage Smad3 protects the infarcted heart, stimulating phagocytosis and regulating inflammation. *Circ. Res.* **125**, 55–70 (2019). 31092129 10.1161/CIRCRESAHA.119.315069 PMC6681442

[68] Kong, P., Christia, P. & Frangogiannis, N. G. The pathogenesis of cardiac fibrosis. *Cell. Mol. Life Sci.* **71**, 549–574 (2014). 23649149 10.1007/s00018-013-1349-6 PMC3769482

[69] Hofmann, U. et al. A collagen α2(I) mutation impairs healing after experimental myocardial infarction. *Am. J. Pathol.* **180**, 113–122 (2012). 22067913 10.1016/j.ajpath.2011.09.033

[70] Buchtler, S. et al. Cellular origin and functional relevance of collagen I production in the kidney. *J. Am. Soc. Nephrol.* **29**, 1859–1873 (2018). 29777019 10.1681/ASN.2018020138 PMC6050926

[71] Molkentin, J. D. et al. Fibroblast-specific genetic manipulation of p38 mitogen-activated protein kinase in vivo reveals its central regulatory role in fibrosis. *Circulation* **136**, 549–561 (2017). 28356446 10.1161/CIRCULATIONAHA.116.026238 PMC5548661

[72] Pesce, M. et al. Cardiac fibroblasts and mechanosensation in heart development, health and disease. *Nat. Rev. Cardiol.* **20**, 309–324 (2023). 36376437 10.1038/s41569-022-00799-2

[73] Papakrivopoulou, J., Lindahl, G. E., Bishop, J. E. & Laurent, G. J. Differential roles of extracellular signal-regulated kinase 1/2 and p38MAPK in mechanical load-induced procollagen α1(I) gene expression in cardiac fibroblasts. *Cardiovasc. Res.* **61**, 736–744 (2004). 14985070 10.1016/j.cardiores.2003.12.018

[74] Lips, D. J. et al. MEK1-ERK2 signaling pathway protects myocardium from ischemic injury in vivo. *Circulation* **109**, 1938–1941 (2004). 15096454 10.1161/01.CIR.0000127126.73759.23

[75] Seif-Naraghi, S. B. et al. Safety and efficacy of an injectable extracellular matrix hydrogel for treating myocardial infarction. *Sci. Transl. Med.* **5**, 173ra125 (2013). 10.1126/scitranslmed.3005503 PMC3848875 23427245

[76] Traverse, J. H. et al. First-in-man study of a cardiac extracellular matrix hydrogel in early and late myocardial infarction patients. *JACC Basic Transl. Sci.* **4**, 659–669 (2019). 31709316 10.1016/j.jacbts.2019.07.012 PMC6834965

[77] Curcio, A. et al. Competitive displacement of phosphoinositide 3-kinase from β-adrenergic receptor kinase-1 improves postinfarction adverse myocardial remodeling. *Am. J. Physiol. Heart Circ. Physiol.* **291**, H1754–H1760 (2006). 16699071 10.1152/ajpheart.01199.2005

[78] Rurik, J. G. et al. CAR T cells produced in vivo to treat cardiac injury. *Science* **375**, 91–96 (2022). 34990237 10.1126/science.abm0594 PMC9983611

[79] Aghajanian, H. et al. Targeting cardiac fibrosis with engineered T cells. *Nature* **573**, 430–433 (2019). 31511695 10.1038/s41586-019-1546-z PMC6752964

[80] Fulgenzi, G. et al. Novel metabolic role for BDNF in pancreatic β-cell insulin secretion. *Nat. Commun.* **11**, 1950 (2020). 32327658 10.1038/s41467-020-15833-5 PMC7181656

[81] Hao, Y. et al. Integrated analysis of multimodal single-cell data. *Cell* **184**, 3573–3587 e3529 (2021). 34062119 10.1016/j.cell.2021.04.048 PMC8238499

[82] McGinnis, C. S., Murrow, L. M. & Gartner, Z. J. DoubletFinder: doublet detection in single-cell RNA sequencing data using artificial nearest neighbors. *Cell Syst.* **8**, 329–337 e324 (2019). 30954475 10.1016/j.cels.2019.03.003 PMC6853612

[83] Love, M. I., Huber, W. & Anders, S. Moderated estimation of fold change and dispersion for RNA-seq data with DESeq2. *Genome Biol*. **15**, 550 (2014). 25516281 10.1186/s13059-014-0550-8 PMC4302049

[84] Wu, T. et al. clusterProfiler 4.0: a universal enrichment tool for interpreting omics data. *Innovation* **2**, 100141 (2021). 34557778 10.1016/j.xinn.2021.100141 PMC8454663

[85] La Manno, G. et al. RNA velocity of single cells. *Nature* **560**, 494–498 (2018). 30089906 10.1038/s41586-018-0414-6 PMC6130801

[86] Bergen, V., Lange, M., Peidli, S., Wolf, F. A. & Theis, F. J. Generalizing RNA velocity to transient cell states through dynamical modeling. *Nat. Biotechnol.* **38**, 1408–1414 (2020). 32747759 10.1038/s41587-020-0591-3

[87] Melzer, M., Beier, D., Young, P. P. & Saraswati, S. Isolation and characterization of adult cardiac fibroblasts and myofibroblasts. *J. Vis. Exp*. 10.3791/60909 (2020). 10.3791/60909 PMC7325628 32225150

[88] Fredriksson, S. et al. Protein detection using proximity-dependent DNA ligation assays. *Nat. Biotechnol.* **20**, 473–477 (2002). 11981560 10.1038/nbt0502-473

[89] Hwang, J. et al. In situ imaging of tissue remodeling with collagen hybridizing peptides. *ACS Nano.* **11**, 9825–9835 (2017). 28877431 10.1021/acsnano.7b03150 PMC5656977

[90] Osei-Amponsa, V. et al. hRpn13 shapes the proteome and transcriptome through epigenetic factors HDAC8, PADI4, and transcription factor NF-κB p50. *Mol. Cell* **84**, 522–537 e528 (2024). 38151017 10.1016/j.molcel.2023.11.035 PMC10872465

[91] Palmieri, E. M. et al. Nitric oxide orchestrates metabolic rewiring in M1 macrophages by targeting aconitase 2 and pyruvate dehydrogenase. *Nat. Commun.* **11**, 698 (2020). 32019928 10.1038/s41467-020-14433-7 PMC7000728

[92] Seaman, S. et al. Eradication of tumors through simultaneous ablation of CD276/B7-H3-positive tumor cells and tumor vasculature. *Cancer Cell* **31**, 501–515 e508 (2017). 28399408 10.1016/j.ccell.2017.03.005 PMC5458750

[93] Westbrook, J. et al. The Protein Data Bank: unifying the archive. *Nucleic Acids Res.* **30**, 245–248 (2002). 11752306 10.1093/nar/30.1.245 PMC99110

[94] Fu, S. et al. The structure of tumor endothelial marker 8 (TEM8) extracellular domain and implications for its receptor function for recognizing anthrax toxin. *PLoS ONE* **5**, e11203 (2010). 20585457 10.1371/journal.pone.0011203 PMC2887854

[95] van Zundert, G. C. P. et al. The HADDOCK2.2 web server: user-friendly integrative modeling of biomolecular complexes. *J. Mol. Biol.* **428**, 720–725 (2016). 26410586 10.1016/j.jmb.2015.09.014

[96] Honorato, R. V. et al. Structural biology in the clouds: the WeNMR-EOSC ecosystem. *Front. Mol. Biosci.* **8**, 729513 (2021). 34395534 10.3389/fmolb.2021.729513 PMC8356364

