# Supplementary material for: ANTXR1 blockade enhances cardiac function in preclinical models of heart failure
Source: Nat Cardiovasc Res. 2025 Oct 2;4(11):1521–38. doi: 10.1038/s44161-025-00725-y (PMC12611763; doi:10.1038/s44161-025-00725-y)
Supplement: Supplementary file 1 — Supplementary Figs. 1–22, Full immunoblot images for Supplementary Figs. 17b and 18. [file 44161_2025_725_MOESM1_ESM.pdf]

nature cardiovascular research

Supplementary information https://doi.org/10.1038/s44161-025-00725-y

ANTXR1 blockade enhances cardiac function in preclinical models of heart failure

In the format provided by the authors and unedited

Inventory of Supplemental Information:

Supplementary Fig. 1 | Survival following myocardial infarction.
Supplementary Fig. 2 | T8Ab improves cardiac performance in female mice following MI.
Supplementary Fig. 3 | T8Ab prevents fibrosis following hypertension.
Supplementary Fig. 4 | T8Ab-FI preserves heart function following hypertension.
Supplementary Fig. 5 | HFD/L-NAME treatment induces glucose intolerance and arterial stiffness.
Supplementary Fig. 6 | Gene expression alterations in epicardial and smooth muscle cells in response to MI and T8Ab treatment.
Supplementary Fig. 7 | Alterations in hematopoietic cells populations in response to T8Ab treatment.
Supplementary Fig. 8 | Alterations in gene expression in CFs in response to myocardial infarction.
Supplementary Fig. 9 | Pathway alterations in CFs in response to myocardial infarction.
Supplementary Fig. 10 | SMAD3 phosphorylation after MI is significantly reduced in ANTXR1 KO mice.
Supplementary Fig. 11 | ANTXR1 mediated alterations in blood endothelial cells following MI.
Supplementary Fig. 12 | T8Ab induced alterations in macrophages following MI.
Supplementary Fig. 13 | Alterations in gene expression in CFs in response to hypertension.
Supplementary Fig. 14 | Alterations in gene expression in all cardiac cell populations in response to hypertension.
Supplementary Fig. 15 | ANTXR1 is expressed in a fraction of cardiac endothelial cells.
Supplementary Fig. 16 | Col1a2-Cre is expressed in cardiac fibroblasts.
Supplementary Fig. 17 | CF transition into a myofibroblast-like state in response to TGFβ.
Supplementary Fig. 18 | MMP14 levels are reduced following ANTXR1 antagonism.
Supplementary Fig. 19 | Quantification of key immunoblots from Figure 7 of main text.
Supplementary Fig. 20 | YAP levels are reduced in the LV scar region of Antxr1 KO mice.
Supplementary Fig. 21 | Antxr1-dependent mRNA alterations in CF in response to TGFβ.
Supplementary Fig. 22 | T8Ab1 and T8Ab2 bind a similar region on the surface of the ANTXR1 ECD

Full immunoblot images for Figures 17B and 18 of supplementary data

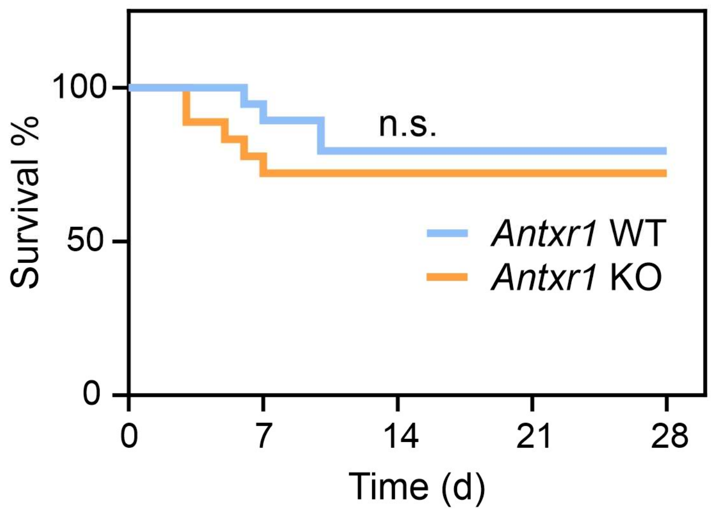

Supplementary Fig. 1 | Survival following myocardial infarction. Kaplan–Meier survival analysis in *Antxr1* WT versus KO mice following MI. P values were from a log-rank (Mantel-Cox) test. N=19/group.

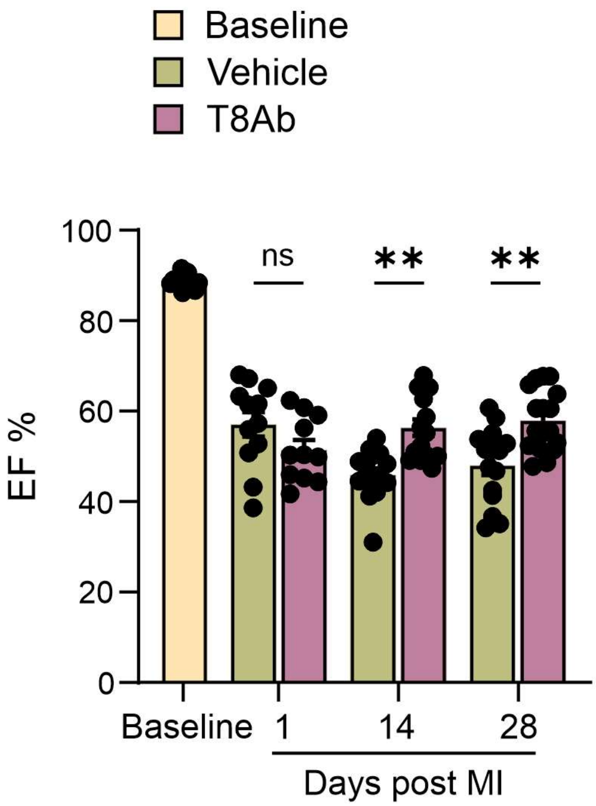

Supplementary Fig. 2 | T8Ab improves cardiac performance in female mice following MI. Echocardiography was used to evaluate the ejection fraction (EF %) at baseline and following MI. Data shown represent the mean ± SEM. *P* values were assessed using a one-way ANOVA with a Tukey's post hoc test. ***P* < 0.01, ns: non-significant. n=15-16/group.

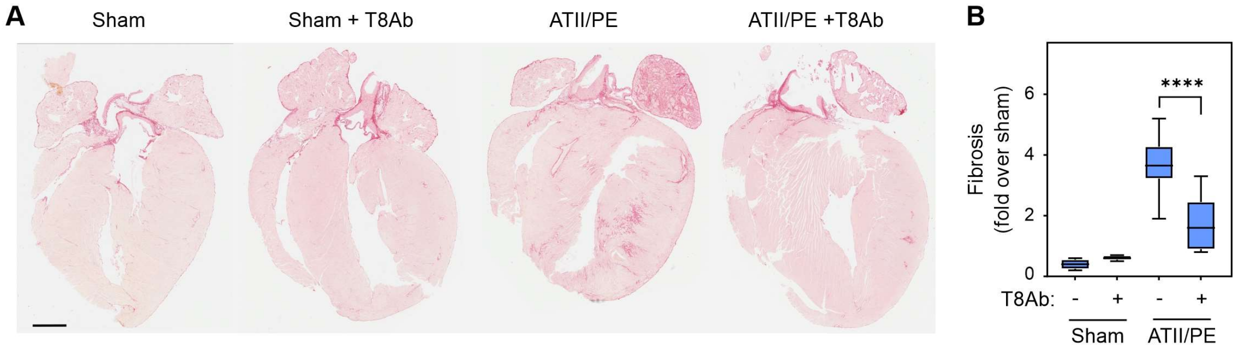

Supplementary Fig. 3 | T8Ab prevents fibrosis following hypertension. A Picrosirius red staining was used to monitor fibrosis after 28 days of exposure to ATII/PE or saline (sham). Treatments with vehicle or T8Ab were administered 3x per week as depicted in Fig. 2F of the main text. A representative sample from each group is shown. Bar: 1 mm. **B** Quantification of the Picrosirius staining. Data shown represent the mean ± SD. N=5/group (vehicle) or 9-10/group (ATII/PE). p-values were assessed using a one-way ANOVA. **** $p < 0.0001$.

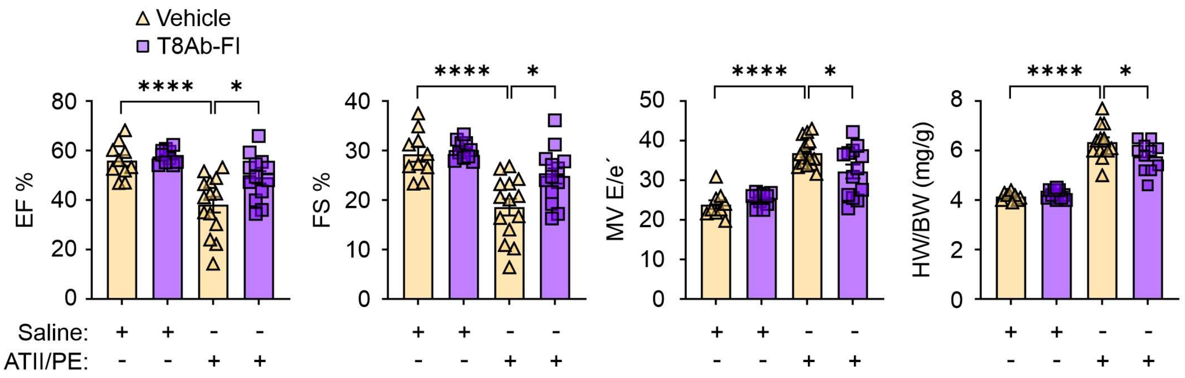

Supplementary Fig. 4 | T8Ab-FI preserves heart function following hypertension. Echocardiography was used to evaluate the ejection fraction (EF %), fractional shortening (FS %) and diastolic function (MV E/e′) in mice after 28 days of exposure to ATII/PE or saline (sham). Heart weight/body weight (HW/BW) ratios were taken at study end. Treatments with vehicle or Fc-inactive T8Ab (T8Ab-FI) were administered 3x per week as depicted in Fig. 2F of the main text. Data shown represent the mean ± SEM. *P* values were assessed using a one-way ANOVA with a Tukey's post hoc test. $^{*}P < 0.05$, $^{****}P < 0.0001$.

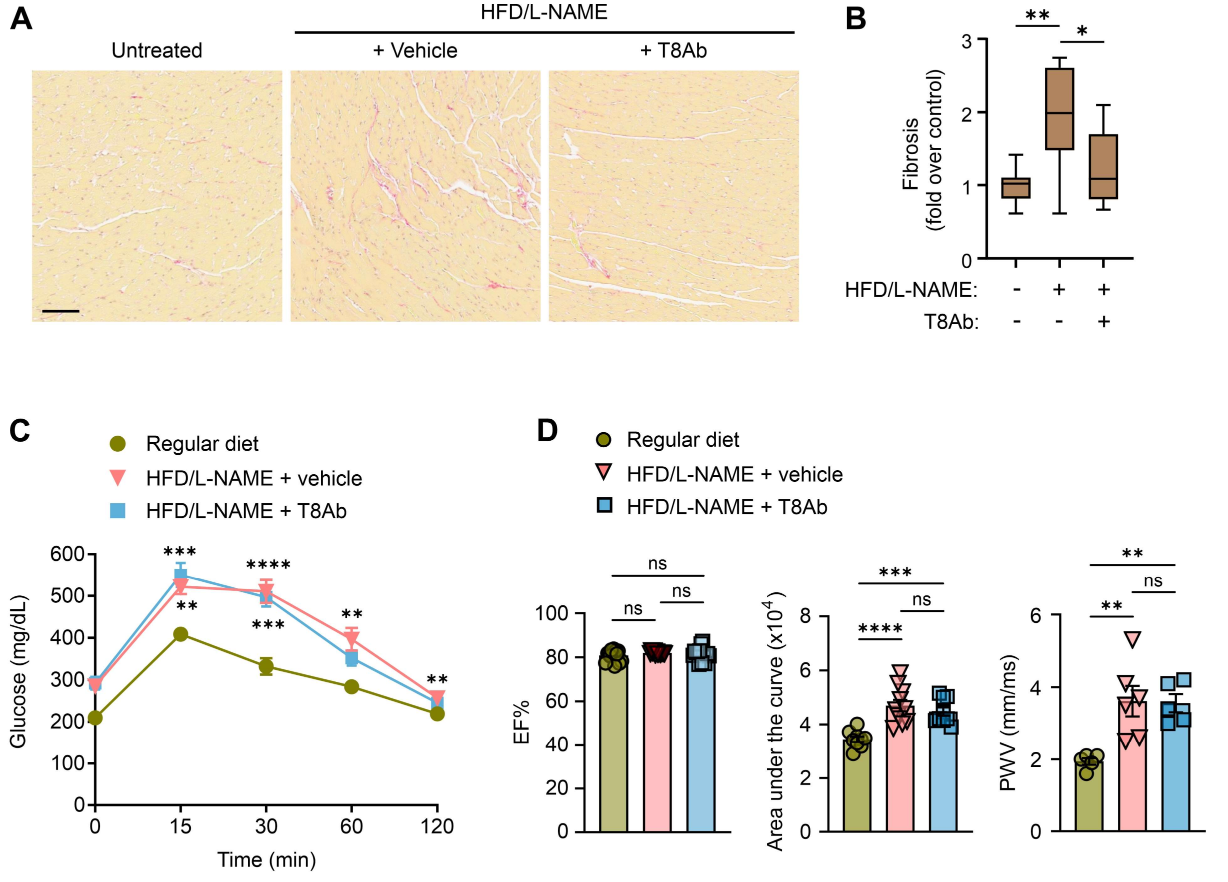

Supplementary Fig. 5 | HFD/L-NAME treatment induces glucose intolerance and arterial stiffness. A Picrosirius red staining was used to monitor fibrosis in mice fed a HFD and treated with L-NAME. Vehicle or T8Ab's were administered 3x per week as depicted in Fig. 3E of the main text. A representative sample from each group is shown. Bar: 100 µm. **B** Quantification of the Picrosirius staining. Data shown represent the mean ± SD. N=8-10/group. p-values were assessed using a one-way ANOVA. *$P < 0.05$, **$P < 0.01$. **C** A glucose tolerance test was used to measure blood glucose levels following a bolus injection of glucose administered 35 days after placing mice on a regular diet + vehicle or a high fat diet (HFD) + L-NAME. n=9-10/group. *P* values represent nearest data point vs. regular diet at same time point. While P values are significant between mice on a regular diet and those on a HFD/L-NAME + vehicle diet at various time points, all *P* values between the HFD/L-NAME + vehicle and the HFD/L-NAME + T8Ab are non-significant (ns). **D** Ejection fraction (EF%), area under the curve (AUC) and Pulse wave velocity (PWV). Glucose intolerance in A was determined from the area under the curve. Note that T8Ab treatment did not impact glucose intolerance. PWV was used to determine arterial stiffness and verify the hypertensive activity of L-NAME. Data shown represent the mean ± SEM. *P* values were assessed using a one-way ANOVA with a Tukey's post hoc test. **$P < 0.01$, ***$P < 0.001$, ****$P < 0.0001$, ns: non-significant.

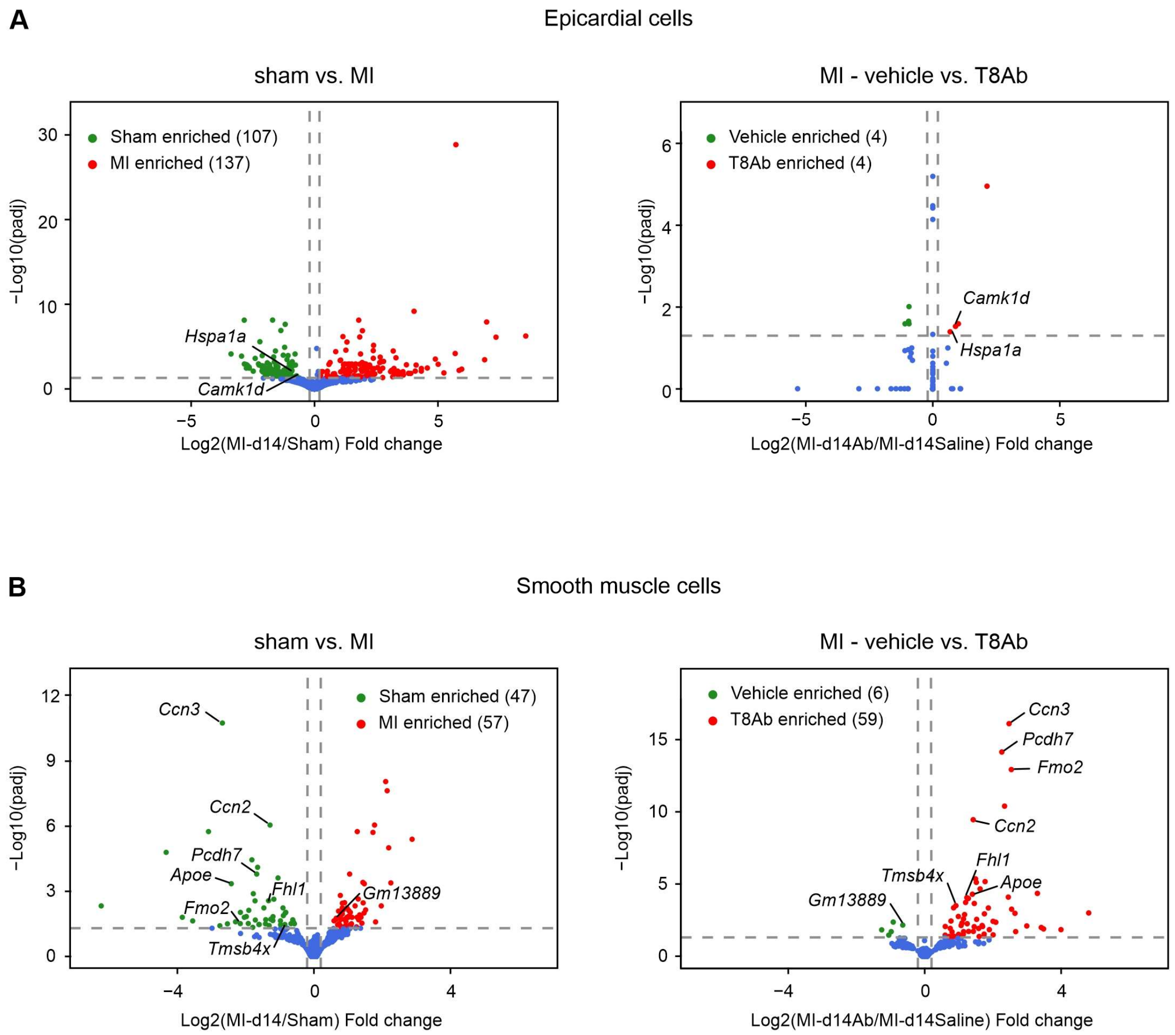

Supplementary Fig. 6 | Gene expression alterations in epicardial and smooth muscle cells in response to MI and T8Ab treatment. A-B Volcano plots depicting differentially expressed genes in epicardial cells (A) or smooth muscle cells (B) in response to MI at d14 vs. sham (left panel) or T8Ab vs. vehicle treatment following MI (right panel). The highlighted genes represent those whose altered expression after MI was reversed by T8Ab treatment.

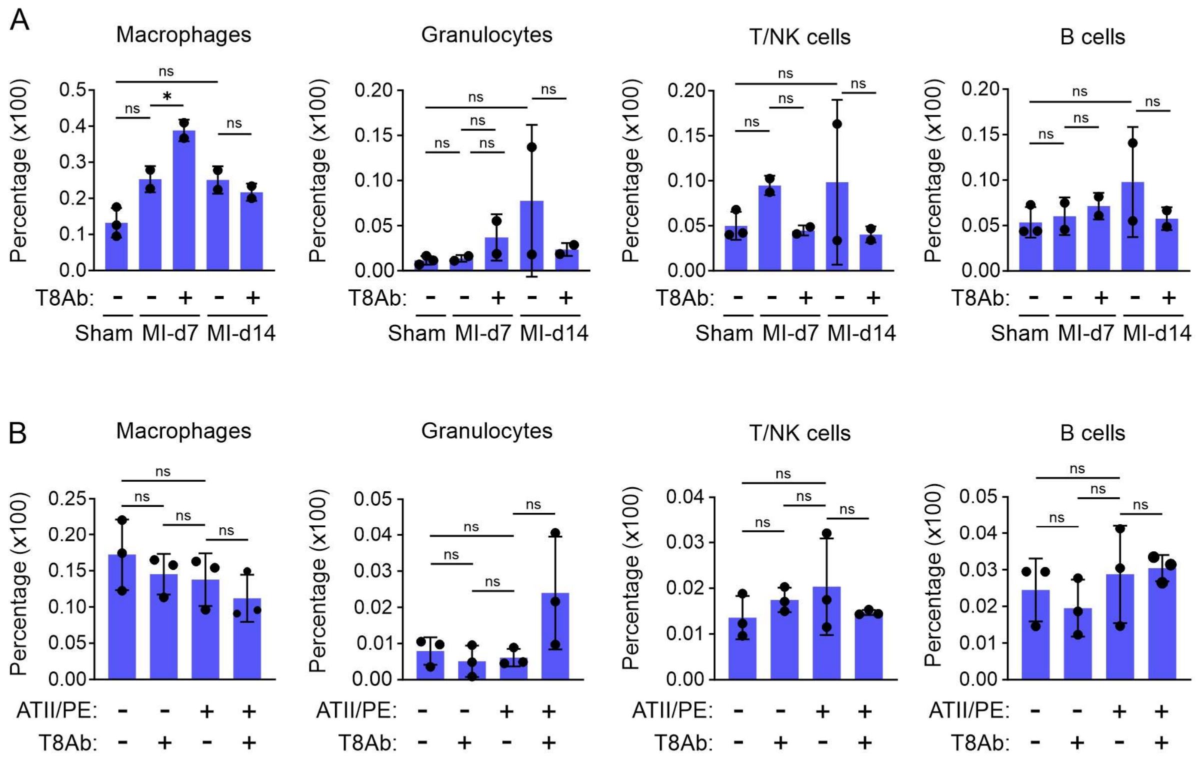

Supplementary Fig. 7 | Alterations in hematopoietic cells populations in response to T8Ab treatment. A-B Bar graphs showing the percentage of each cell type following MI (A) or ATII/PE treatment (B) in the scRNAseq datasets.

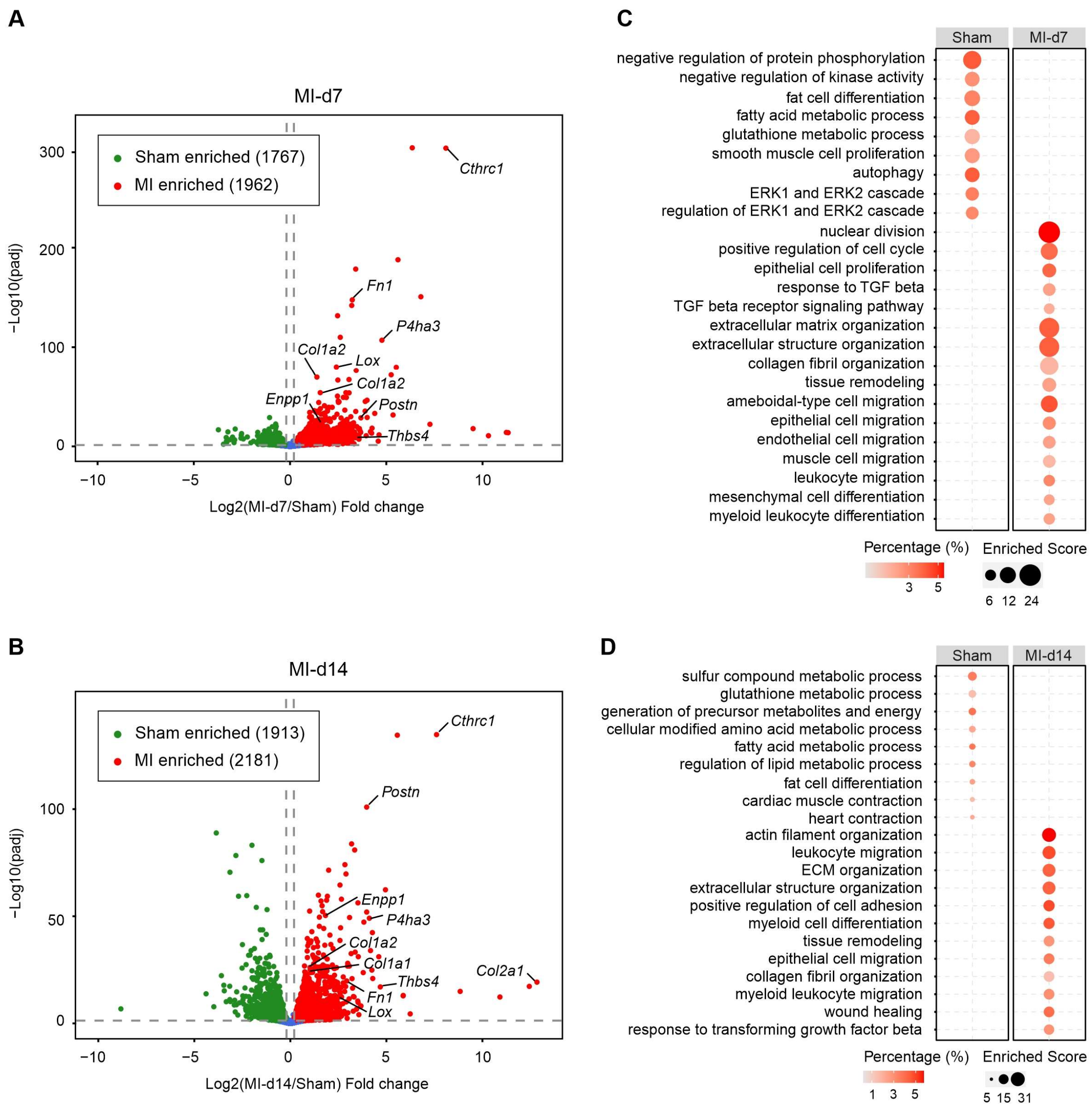

Supplementary Fig. 8 | Alterations in gene expression in CFs in response to myocardial infarction. A-B Volcano plots depicting genes altered in CFs at d7 (A) or d14 (B) post-MI. Selected genes known to be induced in response to MI are highlighted. **C-D** Pathway alterations in CF at d7 (C) or d14 (D) post-MI.

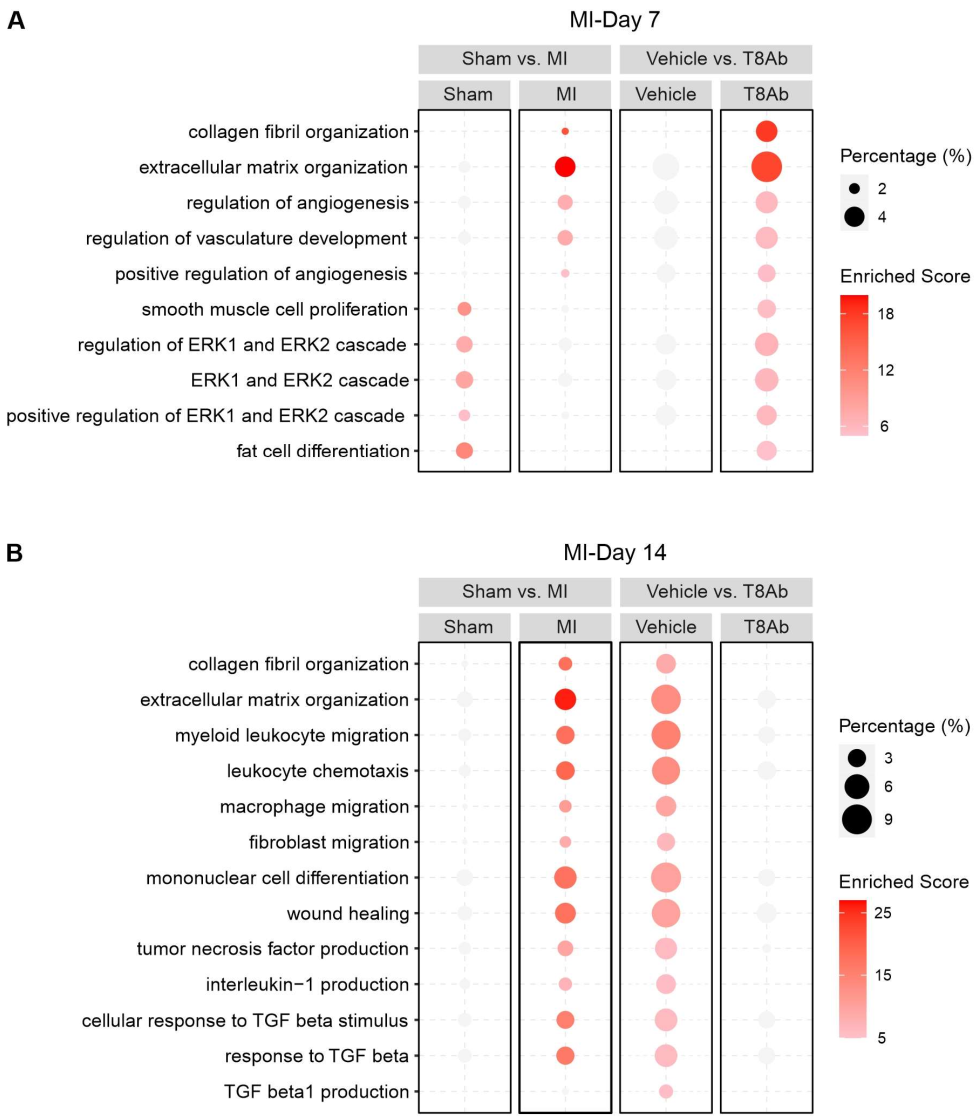

Supplementary Fig. 9 | Pathway alterations in CFs in response to myocardial infarction. A-B Pathway analysis performed on CFs of the indicated groups at d7 (A) or d14 (B) post-MI. Note that T8Ab treatment largely reversed pathway activation by day 14 post-MI.

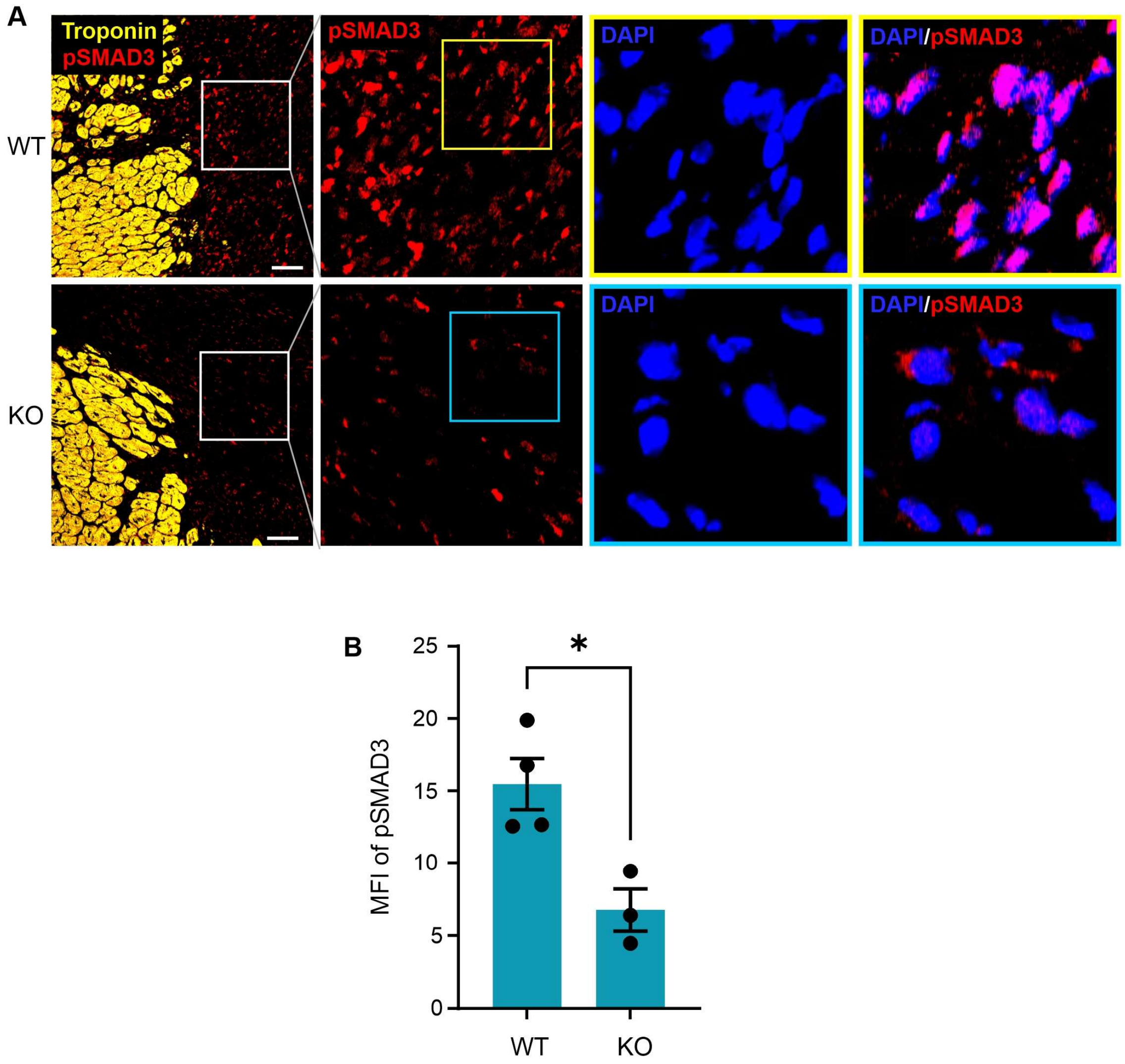

Supplementary Fig. 10 | SMAD3 phosphorylation after MI is significantly reduced in ANTXR1 KO mice. A Immunofluorescence staining of nuclear pSMAD3 (red) levels in the infarct region of ANTXR1 wildtype (WT) and knockout (KO) mice 14 days post-MI. Troponin staining (yellow) shows the cardiomyocytes outside of the infarcted area. Bar: 50 µm. **B** Quantification of the mean fluorescence intensity (MFI) of the nuclear pSMAD3 staining. Note, the nuclear counterstaining with DAPI verified the nuclear location of pSMAD3 (magenta in merge). Data represent the mean ± SEM. *P* values were assessed using a one-way ANOVA. *$P < 0.05$.

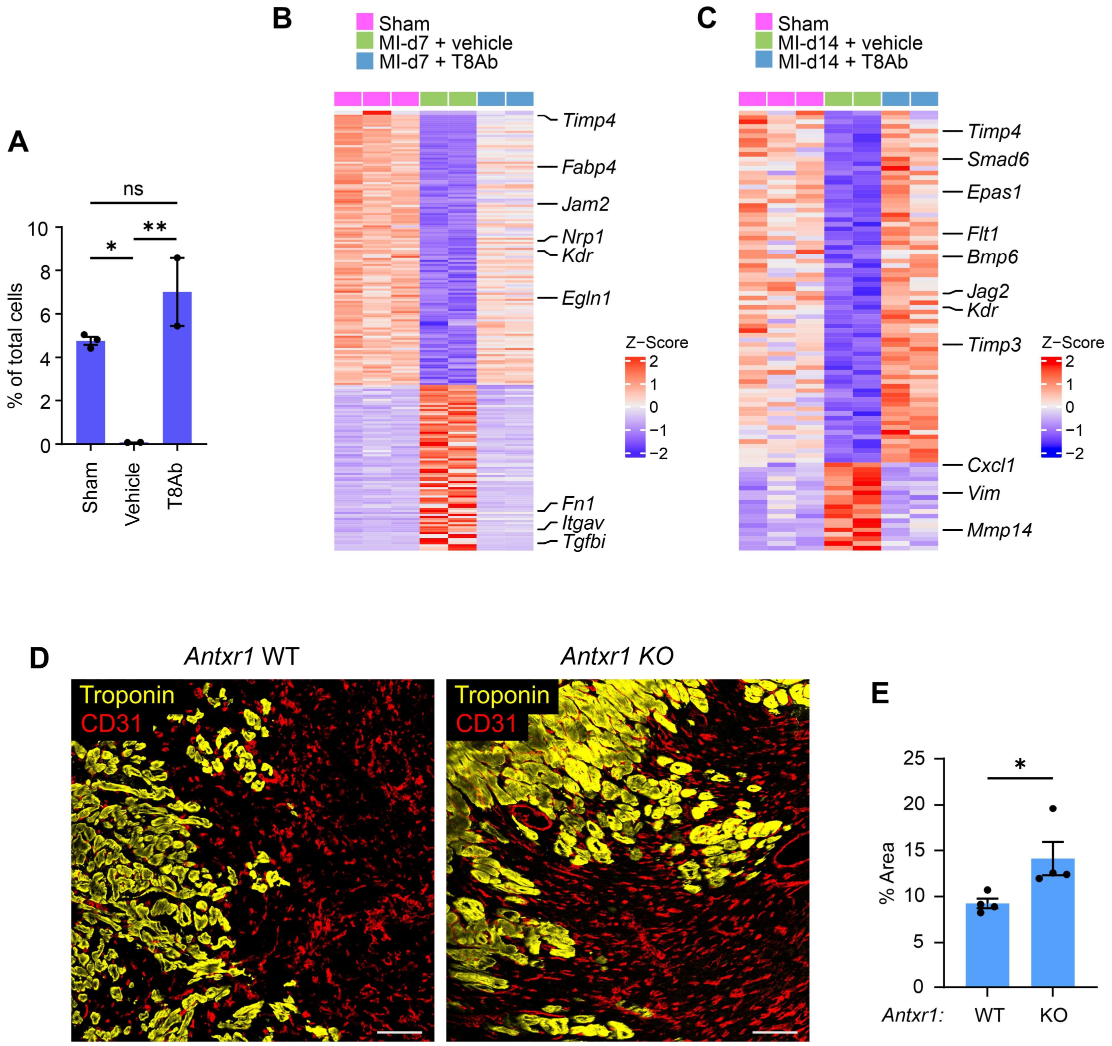

Supplementary Fig. 11 | ANTXR1 mediated alterations in blood endothelial cells (ECs) following MI. A Percentage of blood ECs compared to total cells in the sham group versus the MI group following vehicle or T8Ab treatment at day 7 post surgery. $*P < 0.05$, $**P < 0.01$. ns, not significant. **B-C** Heat map showing genes upregulated or downregulated in blood ECs at day 7 (B) or 14 (C) in vehicle or T8Ab treated mice versus sham controls. Note that T8Ab treatment largely reversed the gene expression alterations in response to MI at both the d7 and d14 time point. **D** Immunofluorescences staining for CD31 (red), a marker of vessels, in the LV of *Antxr1* WT and KO mice 14 days following MI. **E** Quantification of the vessel density from four independent experiments. Bar =100 μm.

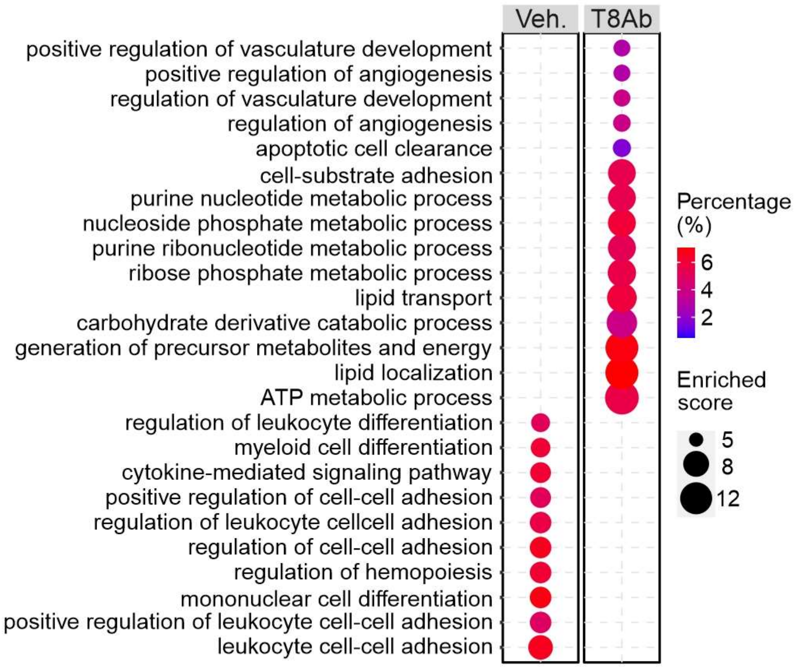

Supplementary Fig. 12 | T8Ab induced alterations in macrophages following MI. GO analysis reveals pathway alterations in macrophages at day 7 post MI in response to T8Ab treatment.

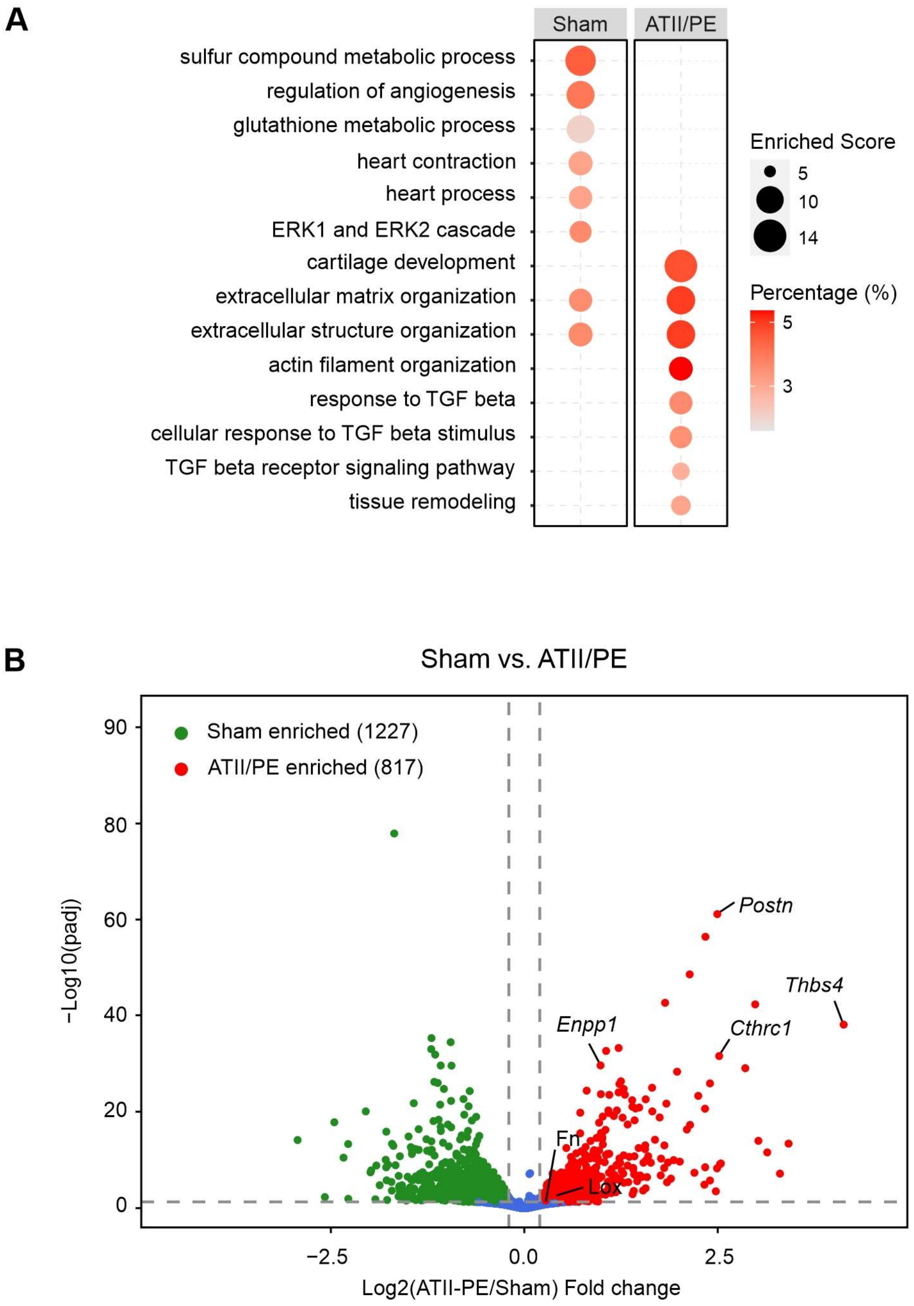

Supplementary Fig. 13 | Alterations in gene expression in CFs in response to hypertension. **A** Pathway alterations in CF after 28 days of exposure to ATII/PE or saline (sham). **B** Volcano plots depicting genes altered in CFs following exposure to ATII/PE or saline. Some genes known to be induced by ATII/PE are highlighted.

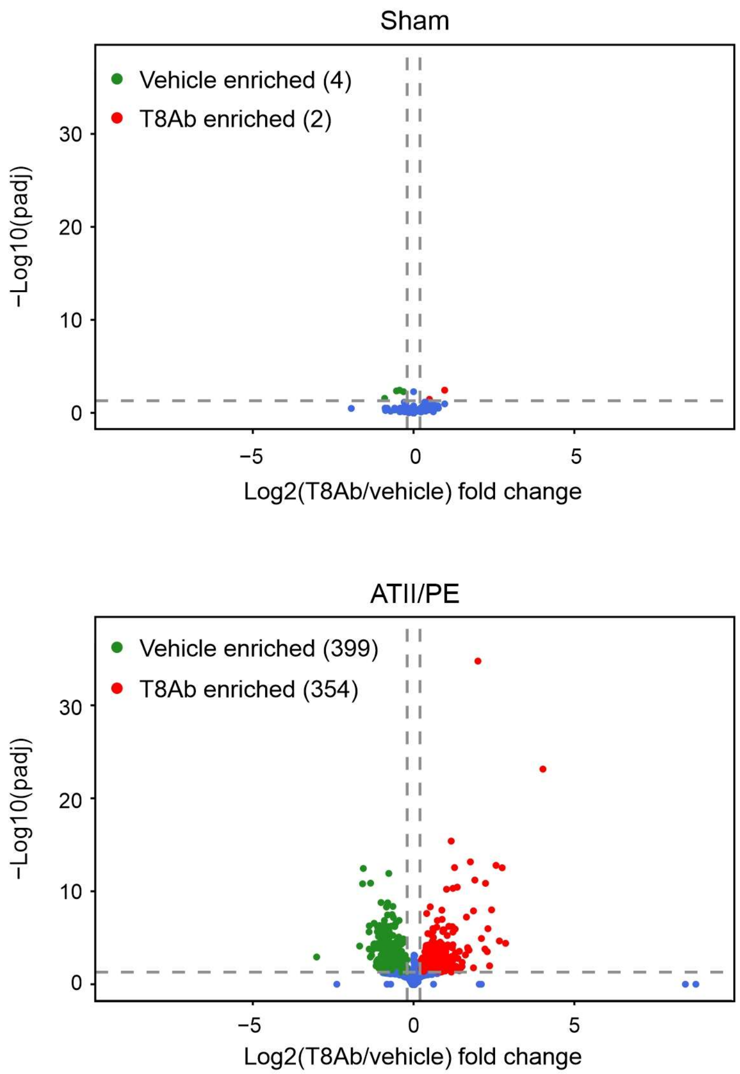

Supplementary Fig. 14 | Alterations in gene expression in all cardiac cell populations in response to hypertension. Volcano plots depicting genes altered in all cardiac cell clusters 28 days after exposure to ATII/PE or saline (sham) and treatment with vehicle or T8Ab.

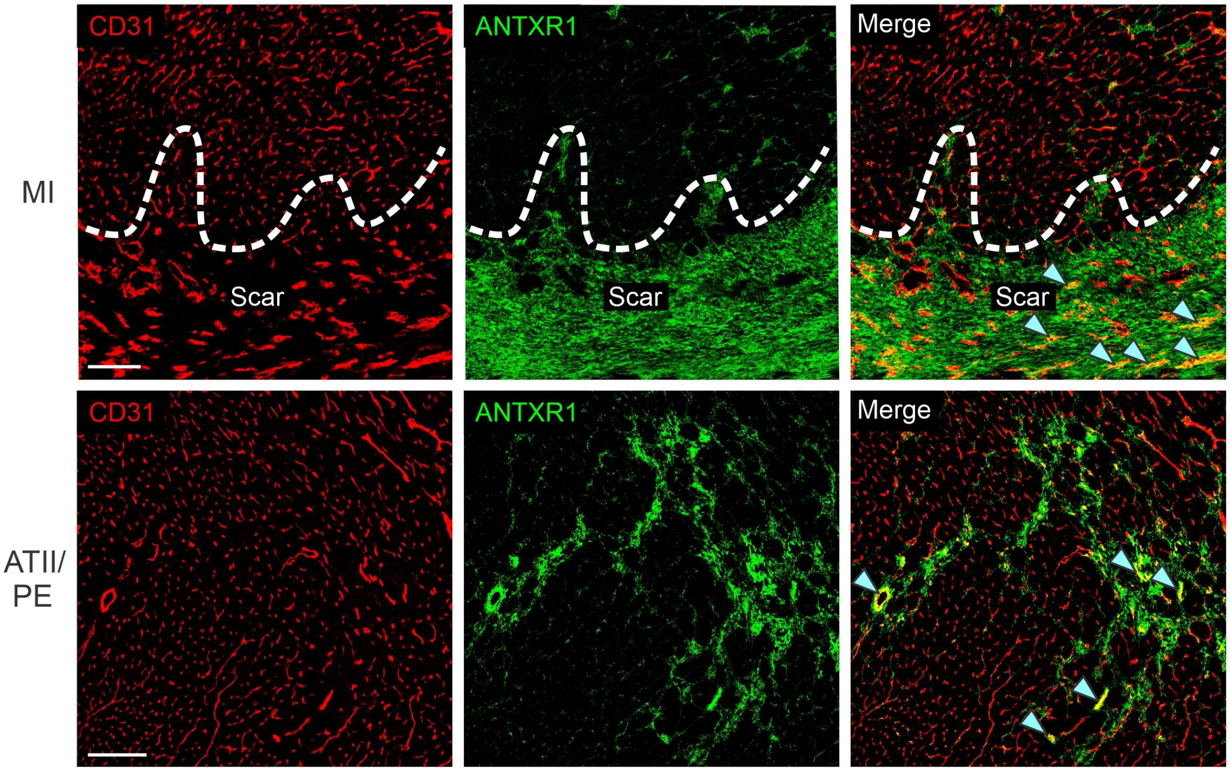

Supplementary Fig. 15 | ANTXR1 is expressed in a fraction of cardiac endothelial cells. Co-IF staining for ANTXR1 (green) and CD31 (red) in the LV 14 days following MI (top panel) or 28 days of treatment with ATII/PE (bottom panel). Bar = 100 µm. Arrowheads highlight ANTXR1 positive endothelial cells.

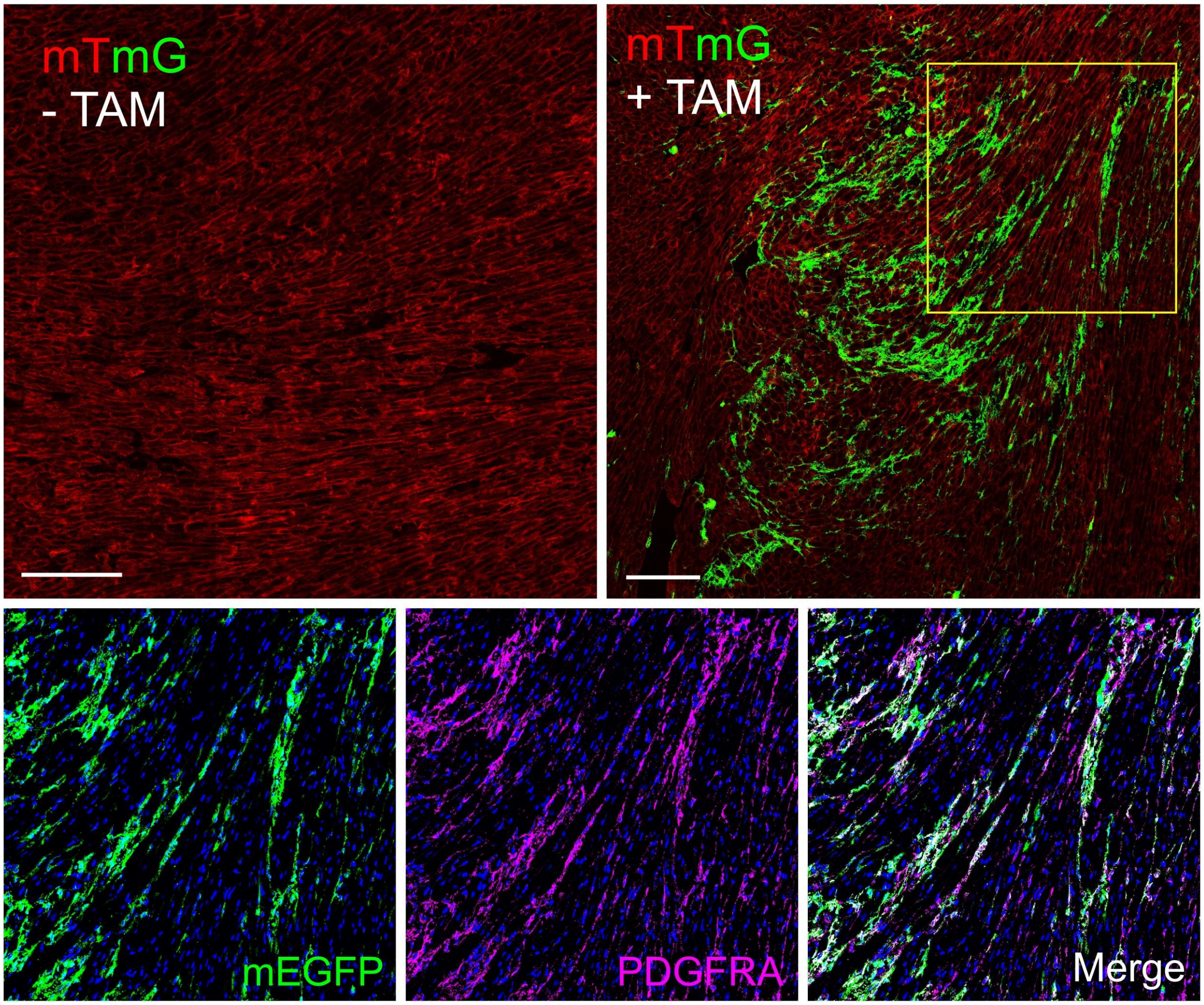

Supplementary Fig. 16 | Col1a2-Cre is expressed in cardiac fibroblasts. To evaluate cre activity, immunofluorescence staining was performed on hearts from mice containing the tamoxifen-inducible Col1a2-CreER transgene and the mTmG reporter. In this experiment, mice were fed a regular (control) diet or a tamoxifen-containing diet and treated with ATII/PE for 28-days. Cre (green) was only detected in mice fed tamoxifen (TAM). The cre-positive cells also co-localized with PDGFRA (magenta), a marker of cardiac fibroblasts. Bar = 200 µm.

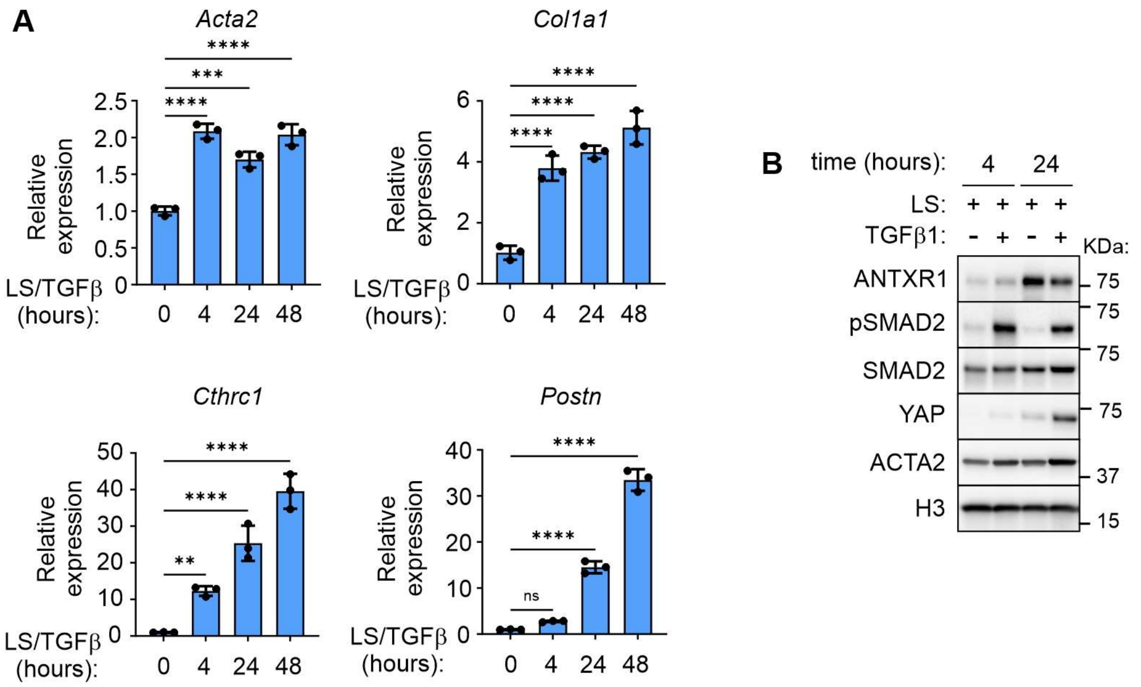

Supplementary Fig. 17 | CF transition into a myofibroblast-like state in response to TGFβ. **A** RT-PCR analysis evaluating the expression of various known TGFβ responsive genes in CFs cultured in low serum (LS) and treated with TGFβ for 4h, 24h or 48. Control cells were cultured in complete media. **B** Immunoblotting analysis verifying the induction of known TGFβ responsive genes, including pSMAD2, YAP and ACTA2, in CF treated with TGFβ. *P*-values were assessed using a one-way ANOVA. *$P < 0.05$, **$P < 0.01$, ***$P < 0.001$, ****$P < 0.0001$. ns, non-significant.

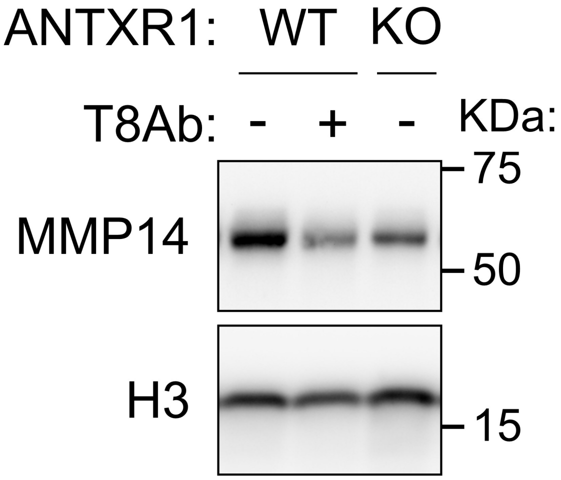

Supplementary Fig. 18 | MMP14 levels are reduced following ANTXR1 antagonism. Immunoblotting assessing the level of MMP14 in ANTXR1 wildtype (WT) cardiac fibroblasts (CF) after T8Ab treatment, or ANTXR1 knockout (KO) CF following treatment with LS/TGFβ for 24 hours. Histone H3 served as a loading control.

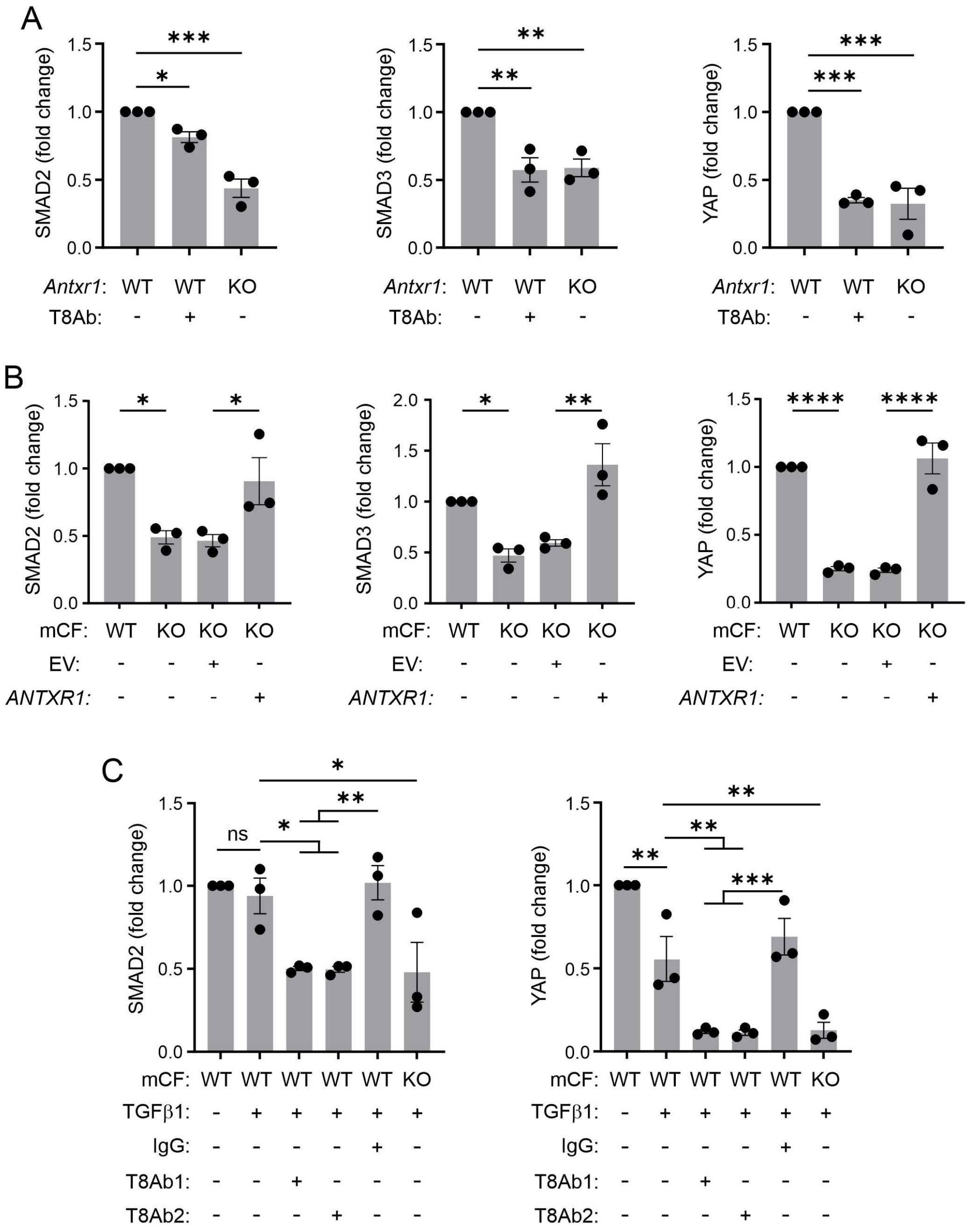

Supplementary Fig. 19 | Quantification of key immunoblots from Figure 7 of main text. A Quantification of SMAD2, SMAD3 and YAP proteins following 1hour of TGFb1 treatment as shown on the immunoblot of Fig. 7F. **B** Quantification of SMAD2, SMAD3 and YAP proteins following ANTXR1 rescue in CF as shown on the immunoblot of Fig. 7M. **C** Quantification of SMAD2 and YAP proteins following ANTXR1 antibody treatment of CF as shown on the immunoblot of Fig. 7P.

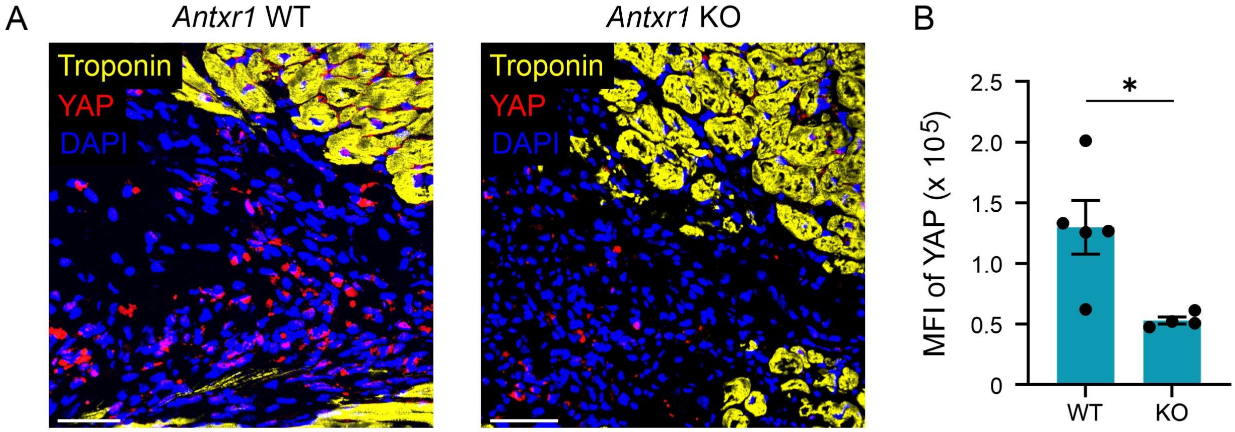

Supplementary Fig. 20 | YAP levels are reduced in the LV scar region of *Antxr1* KO mice. Immunofluorescences staining for YAP (red) in the LV of *Antxr1* WT and KO mice 14 days following MI. Cardiomyocytes were stained with troponin antibodies (yellow) and nuclei were stained with DAPI (blue). **B** Quantification of YAP staining from four independent experiments. Bar =50 µm.

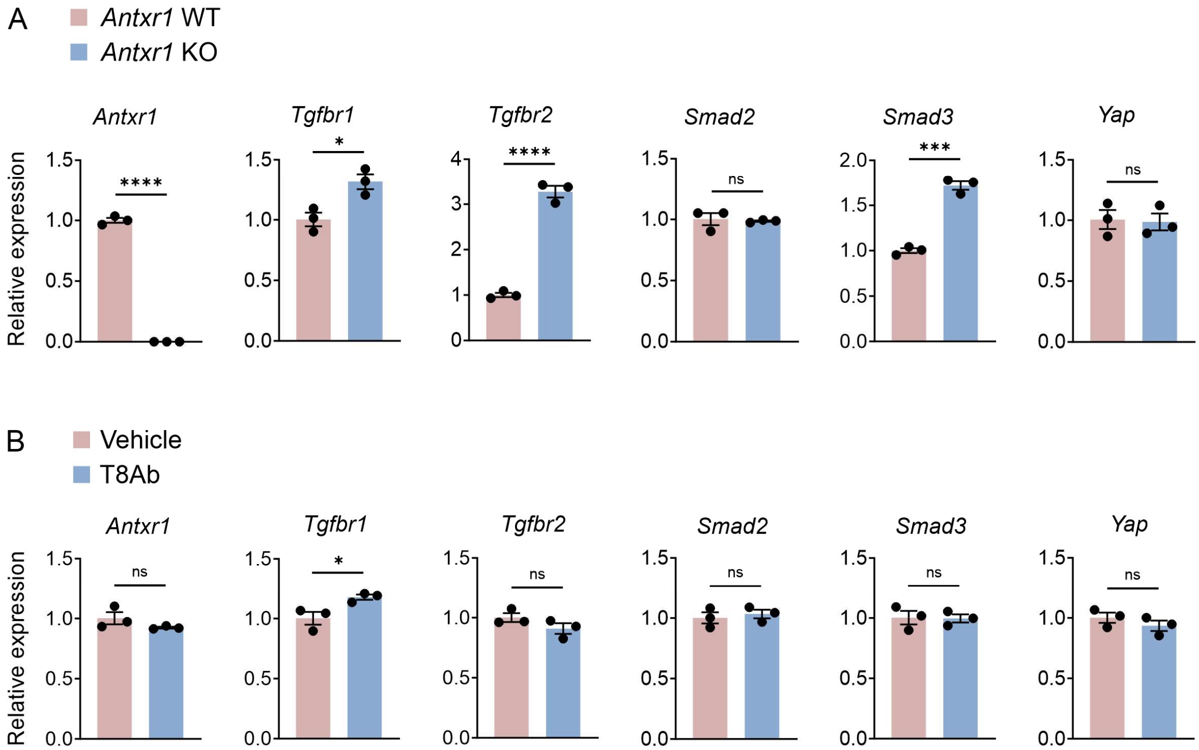

Supplementary Fig. 21 | Antxr1-dependent mRNA alterations in CF in response to TGFβ. **A.** To analyze Antxr1-dependent changes in gene expression, RT-PCR was performed on *Antxr1* WT or KO CF after 24 h of treatment with TGFβ. **B.** RT-PCR was performed on CF treated with TGFβ and T8Abs for 24 h. *$P < 0.05$, ***$P < 0.001$, ****$P < 0.0001$.

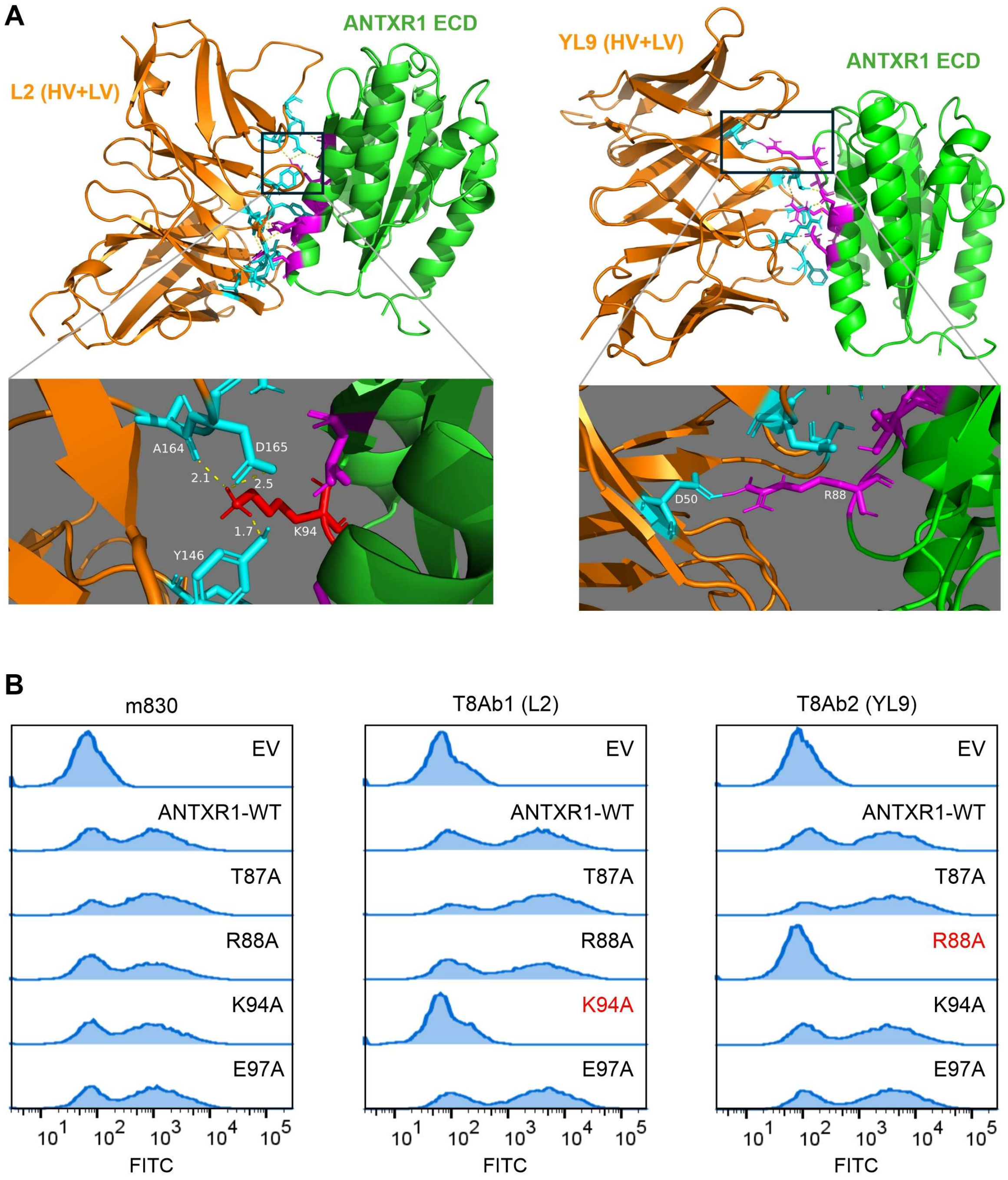

Supplementary Fig. 22 | T8Ab1 and T8Ab2 bind a similar region on the surface of the ANTXR1 ECD. A Antibody docking predicts K94 and R88 at the respective interface between T8Ab1 (L2) and T8Ab2 (YL9) and the ECD of ANTXR1. **B** Flow cytometry showing binding of T8Ab1 (L2) and T8Ab2 (YL9) to CHO cells overexpressing full length wildtype (WT) ANTXR1 or mutant ANTXR1 containing single alanine mutations surrounding the predicted antibody binding site. The two mutations that block binding are highlighted in red. The m830 anti-ANTXR1 antibody was used as a positive control.

Full blot images

Supplementary Fig. 17B

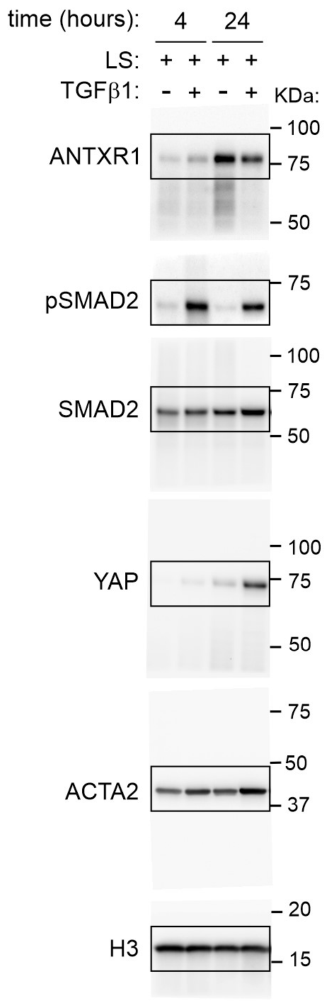

Supplementary Fig. 18

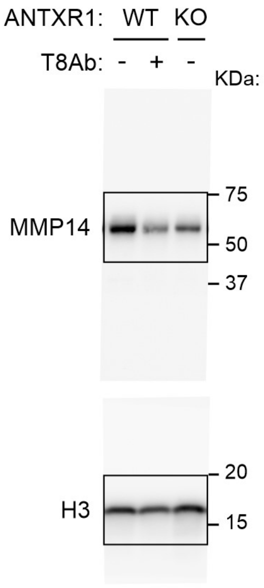

Full immunoblot images for Figures 17B and 18 of supplementary data
