# Supplementary material for: ANTXR1 blockade enhances cardiac function in preclinical models of heart failure
Source: Nat Cardiovasc Res. 2025 Oct 2;4(11):1521–38. doi: 10.1038/s44161-025-00725-y (PMC12611763; doi:10.1038/s44161-025-00725-y)
Supplement: Supplementary file 2 — Reporting Summary [file 44161_2025_725_MOESM2_ESM.pdf]

nature portfolio

Corresponding author(s): Brad St. Croix and Lino Tessarollo

Last updated by author(s): 2025-8-21

Reporting Summary

Nature Portfolio wishes to improve the reproducibility of the work that we publish. This form provides structure for consistency and transparency in reporting. For further information on Nature Portfolio policies, see our Editorial Policies and the Editorial Policy Checklist.

Statistics

For all statistical analyses, confirm that the following items are present in the figure legend, table legend, main text, or Methods section.

n/a	Confirmed	
☐	☒	The exact sample size (*n*) for each experimental group/condition, given as a discrete number and unit of measurement
☐	☒	A statement on whether measurements were taken from distinct samples or whether the same sample was measured repeatedly
☐	☒	The statistical test(s) used AND whether they are one- or two-sided<br>*Only common tests should be described solely by name; describe more complex techniques in the Methods section.*
☒	☐	A description of all covariates tested
☐	☒	A description of any assumptions or corrections, such as tests of normality and adjustment for multiple comparisons
☐	☒	A full description of the statistical parameters including central tendency (e.g. means) or other basic estimates (e.g. regression coefficient) AND variation (e.g. standard deviation) or associated estimates of uncertainty (e.g. confidence intervals)
☐	☒	For null hypothesis testing, the test statistic (e.g. *F*, *t*, *r*) with confidence intervals, effect sizes, degrees of freedom and *P* value noted<br>*Give P values as exact values whenever suitable.*
☒	☐	For Bayesian analysis, information on the choice of priors and Markov chain Monte Carlo settings
☒	☐	For hierarchical and complex designs, identification of the appropriate level for tests and full reporting of outcomes
☒	☐	Estimates of effect sizes (e.g. Cohen's *d*, Pearson's *r*), indicating how they were calculated

Our web collection on statistics for biologists contains articles on many of the points above.

Software and code

Policy information about availability of computer code

Data collection	The fastq files were aligned to the database of the mouse genome (refdata-gex-mm10-2020-A) using cellranger (v7.0.0, 10x Genomics). A Fujifilm VisualSonics Vevo2100 Ultrasound System (VisualSonics Inc, Toronto, ON, Canada) was used for echocardiography. Glucose concentrations were measured using the Alphatrak®2 glucometer (Zoetis). A dissecting microscope (ZEISS SteREO Discovery.V20) was used for the collagen contraction assay. Flow cytometry data was collected on a BD LSRFortessa apparatus.
Data analysis	The h5 files from the output folder were analyzed using the Seurat (v4.1.1). For differentially expressed genes, the pseudo-bulk RNA-seq method with DESeq2 (v1.36.0) was adopted. The clusterProfiler (v4.4.4)58 was used for the GO enrichment analysis. The volcano plots and heatmap were plotted using ggplot2(v3.4.3) and ComplexHeatmap (v2.15.1), separately. For the RNA velocity analysis, velocyto (v0.17) and scvelo (v0.2.4) were used. Other data analyses software: GraphPad Prism software 10.2.0 - Fiji (v2.14.0) - Vevo Lab 2100 software - a BD LSRFortessa apparatus - FlowJo software (v10.8.1) - HADDOCK2.4 server

For manuscripts utilizing custom algorithms or software that are central to the research but not yet described in published literature, software must be made available to editors and reviewers. We strongly encourage code deposition in a community repository (e.g. GitHub). See the Nature Portfolio guidelines for submitting code & software for further information.

Data

Policy information about availability of data

All manuscripts must include a data availability statement. This statement should provide the following information, where applicable:

- Accession codes, unique identifiers, or web links for publicly available datasets
- A description of any restrictions on data availability
- For clinical datasets or third party data, please ensure that the statement adheres to our policy

Single-cell RNA-seq data have been deposited at GEO: GSE266597and are publicly available as of the date of publication. This paper also analyzes existing, publicly available data from GSE183852 and Broad Institute's Single Cell Portal under project ID SCP1303: https://singlecell.broadinstitute.org/single_cell/study/SCP1303/.

Research involving human participants, their data, or biological material

Policy information about studies with human participants or human data. See also policy information about sex, gender (identity/presentation), and sexual orientation and race, ethnicity and racism.

Reporting on sex and gender	ANTXR1 expression was evaluated in human injured hearts from both male and females, see supplementary Table 1.
Reporting on race, ethnicity, or other socially relevant groupings	Yes, reported in supplementary Table 1. All patients were White/Caucasian or Black/African American.
Population characteristics	Reported in manuscript supplementary Table 1.
Recruitment	Anonymised HCM and DCM patients samples from the Duke or CHTN repository were obtained from individuals with hypertension. Non-failing hearts were from individuals with similar age, sex and ethnicity.
Ethics oversight	Anonymised human samples were obtained from the Duke repository with approval from the Duke University Institutional Review Board. Samples from the CHTN repository were obtained with approval from the NIH Office of Human Subject Research.

Note that full information on the approval of the study protocol must also be provided in the manuscript.

Field-specific reporting

Please select the one below that is the best fit for your research. If you are not sure, read the appropriate sections before making your selection.

☒ Life sciences ☐ Behavioural & social sciences ☐ Ecological, evolutionary & environmental sciences

For a reference copy of the document with all sections, see nature.com/documents/nr-reporting-summary-flat.pdf

Life sciences study design

All studies must disclose on these points even when the disclosure is negative.

Sample size	Samples sizes for mouse studies were kept as large as possible, keeping practical considerations in mind - they were governed by availability of ANTXR1 WT, KO and floxed mice, cage space and the number of surgeries that could be preformed in a single day, and literature conducting similar experiments to achieve statistical differences between groups.
Data exclusions	Statistical outliers - values which can arise from technical artifacts in electrocardiography data (e.g., blurry images) - were excluded based on predefined criteria established prior to analysis. Outliers were flagged using GraphPad Prism's ROUT method with a predefined Q value of 1%. This approach applies robust regression to establish a baseline, then uses the FDR framework to identify data points that deviate sufficiently from the model to be classified as outliers. By setting Q to 1%, we ensured that no more than 1% of detected outliers are expected to be false positives - i.e., at least 99% are true outliers. The final sample size remained adequately large to ensure that exclusion of outliers did not materially influence the overall conclusions.
Replication	All in vitro experiments were repeated at least three times and all in vivo experiments were repeated at least twice. All attempts to replicate data were successful.
Randomization	For mice of the same age, animal cages were randomly allocated in our animal facility. For mice that varied in age (i.e. ANTXR1 WT versus het vs KO), mice were randomized into groups containing the same average age and body weight.
Blinding	For scRNA-seq analysis, we provided our sequencing core with only a numerical sample list, ensuring that the identities connected to each number were unknown at the time of data generation. Although the bioinformatician performing downstream analysis was later informed of group assignment, this occurred only after sequencing; we had no prior expectations or knowledge of the results. All blinding steps were predefined and maintained through data acquisition and sequencing, with unblinding limited to the interpretation phase. For Western blot experiments, although the investigator knew the loading order, equal loading was verified using loading controls to mitigate bias. For in vivo echocardiography, all investigators conducting data acquisition and analysis were fully blinded to both study group and genotype. Survival

endpoints were recorded by animal technicians who were blinded to treatment group/genotype. Blinding procedures were established prior to data collection, and no unblinding occurred until analyses were complete.

Reporting for specific materials, systems and methods

We require information from authors about some types of materials, experimental systems and methods used in many studies. Here, indicate whether each material, system or method listed is relevant to your study. If you are not sure if a list item applies to your research, read the appropriate section before selecting a response.

Materials & experimental systems

n/a	Involved in the study
☐	☒ Antibodies
☐	☒ Eukaryotic cell lines
☒	☐ Palaeontology and archaeology
☐	☒ Animals and other organisms
☒	☐ Clinical data
☒	☐ Dual use research of concern
☒	☐ Plants

Methods

n/a	Involved in the study
☒	☐ ChIP-seq
☐	☒ Flow cytometry
☒	☐ MRI-based neuroimaging

Antibodies

Antibodies used

Anti-ANTXR1 rabbit mAb, clone c37, Abcam, ab241067
Anti-CD31/PECAM, clone MEC13.3, Santa Cruz, sc-18916
Anti-CD105, clone MJ7/18, eBioscience, 14-1051-85
Anti-Col1a1, Cell Signaling Technology, 72023T
Anti-GFP (chicken), Abcam, ab13970
Anti-Histone 3, Abcam, ab1791
Anti-SMAD3, clone C67H9, Cell Signaling Technology, 9523S
Anti-SMAD2, clone D43B4, Cell Signaling Technology, 5339S
Anti-pSMAD3, clone EP823Y, Abcam ab52903
Anti-pSMAD2, clone 138D4, Cell Signaling Technology, 3108S
Anti-MMP14, clone EP1264Y, Abcam, ab51074
Anti-αSMA, Millipore, ABT1487
Anti-TGFBR1, clone EPR20923-13, Abcam, ab235578
Anti-TGFBR2, ThermoFisher, BS-0117R
Anti-Troponin TC, clone: 1A11, Santa Cruz Biotechnology, sc-52281
Anti-YAP, clone D8H1X, Cell Signaling Technology, 14074S
Anti-pYAP, Cell Signaling Technology, 4911S
AF647-mouse anti-cardiac troponin T, clone13-11, BD Biosciences, 565744
AF680-WGA, ThermoFIsher, W32465
APC-anti-cardiac troponin I, Abcam, ab305815
Anti-THBS4, clone EPR22922-232, Abcam, ab263898
Anti-SMAD2/3, BD Biosciences, 610843
Anti-TGRBR1, Sigma, ABF17-I
Anti-PDGFRα, R&D Systems, AF1062
human IgG, SouthernBiotech, 0151K-01
rat IgG2a isotype control, Invitrogen, 02-9688
Rabbit IgG, Abcam, ab172730
FITC-Anti-human IgG (H+L), Bethyl, A80-219F
AF594 donkey anti-mouse IgG (H+L), Jackson Immunoresearch, 715-585-150
AF594 donkey anti-rat IgG (H+L), Jackson Immunoresearch, 715-585-153
AF594-donkey anti-rabbit IgG (H+L), Jackson Immunoresearch, 711-585-152
Goat-anti-rabbit IgG (H+L), SouthernBiotech, 4049-05
FITC goat anti-rabbit IgG (H+L), Jackson Immunoresearch, 111-095-144
AF488-donkey anti-goat IgG (H+L), Jackson Immunoresearch, 705-545-147
AF488-donkey anti-chicken IgG (H+L), Jackson Immunoresearch, 703-545-155

Validation

The validation data are available at the following websites:
Anti-ANTXR1 rabbit mAb, c37, Abcam, ab241067; https://www.jci.org/articles/view/120481
Anti-Histone 3, Abcam, ab1791, https://www.abcam.com/en-us/products/primary-antibodies/histone-h3-antibody-nuclear-marker-and-chip-grade-ab1791#
Anti-SMAD3, Cell Signaling Technology, 9523S, https://www.cellsignal.com/products/primary-antibodies/smad3-c67h9-rabbit-mab/9523
Anti-SMAD2, Cell Signaling Technology, 5339S, https://www.cellsignal.com/products/primary-antibodies/smad2-d43b4-xp-rabbit-mab/5339
Anti-pSMAD3, Abcam ab52903, https://www.abcam.com/en-us/products/primary-antibodies/smad3-phospho-s423-s425-antibody-ep823y-ab52903
Anti-pSMAD2, Cell Signaling Technology, 3108S, https://www.cellsignal.com/products/primary-antibodies/phospho-smad2-ser465-467-138d4-rabbit-mab/3108

Anti-MMP14, Abcam, ab51074, https://www.abcam.com/en-us/products/primary-antibodies/mmp14-antibody-ep1264y-ab51074
Anti-αSMA, Millipore, ABT1487, https://www.emdmillipore.com/US/en/product/Anti-alpha-Actin-Antibody-Smooth-Muscle,MM_NF-ABT1487
Anti-TGFBR1, Abcam, ab235578, https://www.abcam.com/en-us/products/primary-antibodies/tgf-beta-receptor-i-antibody-epr20923-13-ab235578
Anti-TGFBR2, ThermoFisher, BS-0117R, https://www.thermofisher.com/antibody/product/TGF-beta-Receptor-2-Antibody-Polyclonal/BS-0117R
Anti-YAP, Cell Signaling Technology, 14074S, https://www.cellsignal.com/products/primary-antibodies/yap-d8h1x-xp-rabbit-mab/14074
Anti-pYAP, Cell Signaling Technology, 4911S, https://www.cellsignal.com/products/primary-antibodies/phospho-yap-ser127-antibody/4911
AF647-mouse anti-cardiac troponin T, BD Biosciences, 565744, https://www.bdbiosciences.com/en-us/products/reagents/flow-cytometry-reagents/research-reagents/single-color-antibodies-ruo/alexa-fluor-647-mouse-anti-cardiac-troponin-t.565744
APC anti-cardiac troponin I, Abcam, ab305815, https://www.abcam.com/en-us/products/primary-antibodies/apc-cardiac-troponin-i-antibody-ep1106y-ab305815
Anti-THBS4, Abcam, ab263898, https://www.abcam.com/en-us/products/primary-antibodies/thbs4-antibody-epr22922-232-ab263898
Anti-SMAD2/3, BD Biosciences, 610843, https://www.bdbiosciences.com/en-us/products/reagents/microscopy-imaging-reagents/immunofluorescence-reagents/purified-mouse-anti-smad2-3.610843
Anti-TGRBR1, Sigma, ABF17-I, https://www.sigmaaldrich.com/US/en/product/mm/abf17i?srsltid=AfmBOorjEHp2ID57uRlwbraHwBBwbWeUy0uS0aBxXciXL6c5qZnVbbxF
Anti-PDGFRα, R&D Systems, AF1062, https://www.rndsystems.com/products/mouse-pdgf-ralpha-antibody_af1062
Human IgG, SouthernBiotech, 0151K-01, https://www.southernbiotech.com/human-igg1-kappa-unlb-0151k-01
Rabbit IgG, Abcam, ab172730, https://www.abcam.com/en-us/products/primary-antibodies/rabbit-igg-monoclonal-epr25a-isotype-control-ab172730
FITC-Anti-human IgG (H+L), Bethyl, A80-219F, https://www.fortislife.com/products/secondary-antibodies/goat-anti-human-igg-heavy-and-light-chain-cross-adsorbed-antibody/BETHYL-A80-219
AF594-donkey anti-rabbit IgG (H+L), Jackson Immunoresearch, 711-585-152, https://www.jacksonimmuno.com/catalog/products/711-585-152
Goat-anti-rabbit IgG (H+L), SouthernBiotech, 4049-05, https://www.southernbiotech.com/goat-anti-rabbit-igg-h-l-mouse-rat-human-ads-hrp-4049-05
FITC goat anti-rabbit IgG (H+L), Jackson Immunoresearch, 111-095-144, https://www.jacksonimmuno.com/catalog/products/111-095-144
AF594 donkey anti-mouse IgG (H+L), Jackson Immunoresearch, 715-585-150, https://www.jacksonimmuno.com/catalog/products/715-585-150
AF488-donkey anti-goat IgG (H+L), Jackson Immunoresearch, 705-545-147, https://www.jacksonimmuno.com/catalog/products/705-545-147

Eukaryotic cell lines

Policy information about cell lines and Sex and Gender in Research

Cell line source(s)	The mouse cardiac fibroblasts were derived mice containing ANTXR1 "floxed" alleles and the SV40 TAg transgene (Immortomouse, The Jackson Laboratory) as described in the methods. Primary human ventricular cardiac fibroblasts (NHCF-V, Cat# CC-2904, Lonza). CHO cells were obtained from Dr. Steve Leppla.
Authentication	The cardiac fibroblasts were authenticated by treating with TGFbeta and looking for markers of myofibroblast activation as described in the manuscript. No other authentication was performed on the primary cells or the CHO cells.
Mycoplasma contamination	The primary human ventricular cardiac fibroblasts (Lonza) and CHO cells were screened for mycoplama contamination by the manufacturer. The cardiac fibroblasts derived from mice were not tested for mycoplasma.
Commonly misidentified lines (See ICLAC register)	None.

Animals and other research organisms

Policy information about studies involving animals; ARRIVE guidelines recommended for reporting animal research, and Sex and Gender in Research

Laboratory animals	Mouse strain used: C57BL6/NCrl or ANTXR1-/- mice which were derived from ANTXR1-flox/flox mice (PMID: 36400786). The col1a2-creER, mTmG reporter and and Immortomouse were from The Jackson Laboratory. All mice were 8-12 weeks old age matched.
Wild animals	None used.
Reporting on sex	Adult male and female mice (8-12-weeks-old) were used for the animal experiments as indicated in the text.
Field-collected samples	None were used in the study.
Ethics oversight	The clinical samples were approved for us by the the Duke University Institutional Review Board under protocol number Pro00005621. All animal studies were reviewed and approved by the NCI Frederick Animal Care and Use committee, as indicated in the methods.

Note that full information on the approval of the study protocol must also be provided in the manuscript.

Plants

Seed stocks	none
Novel plant genotypes	none
Authentication	none

Flow Cytometry

Plots

Confirm that:

- [x] The axis labels state the marker and fluorochrome used (e.g. CD4-FITC).
- [x] The axis scales are clearly visible. Include numbers along axes only for bottom left plot of group (a 'group' is an analysis of identical markers).
- [ ] All plots are contour plots with outliers or pseudocolor plots.
- [ ] A numerical value for number of cells or percentage (with statistics) is provided.

Methodology

Sample preparation	See the methods section of the manuscript for details regarding sample preparation.
Instrument	BD LSRFortessa apparatus.
Software	Data was analyzed using FlowJo software (v10.8.1).
Cell population abundance	The exact percentages were not calculated in supplementary figure 20 because the two ANTXR1 mutants completely abolished antibody binding.
Gating strategy	CHO cells transected with empty vector were used to set the gates. ANTXR1 transfection resulted in a new population of ANTXR1 antibody binding cells that was absent from the EV control. Using this setup, mutants that completely blocked binding were readily identified, and a control anti-ANTXR1 antibody (m830) was used to verify expression of all the mutants on the cell surface.

- [x] Tick this box to confirm that a figure exemplifying the gating strategy is provided in the Supplementary Information.
