# Supplementary figures and images for: ANTXR1 blockade enhances cardiac function in preclinical models of heart failure
Source: Nat Cardiovasc Res. 2025 Oct 2;4(11):1521–38. doi: 10.1038/s44161-025-00725-y (PMC12611763; doi:10.1038/s44161-025-00725-y)

## Full blot images

**Fig. 1b**

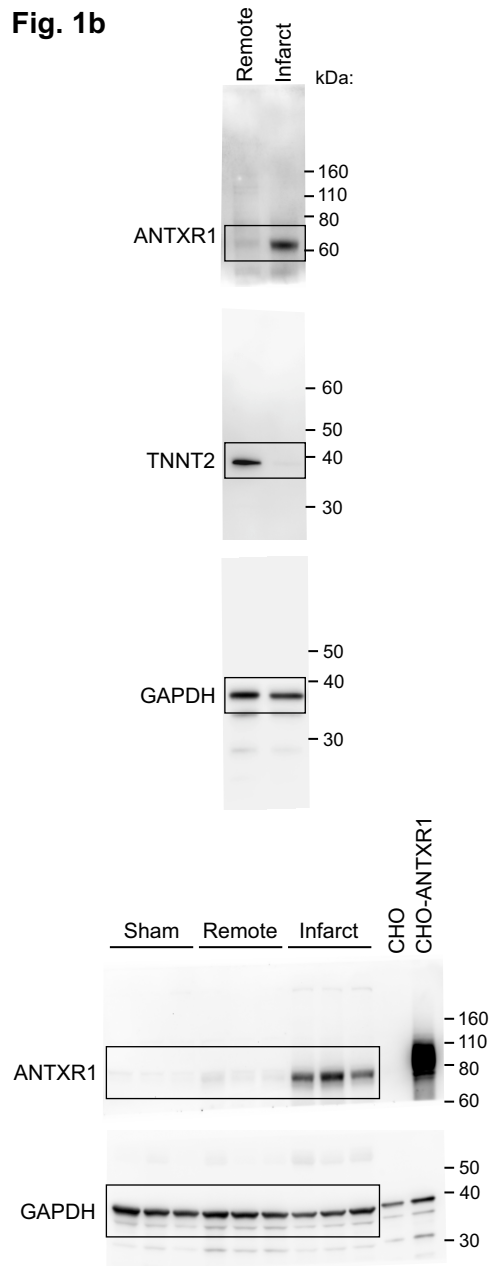

**Fig. 1e**

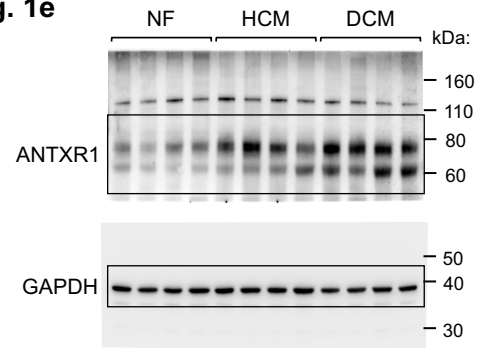

**Fig. 1g**

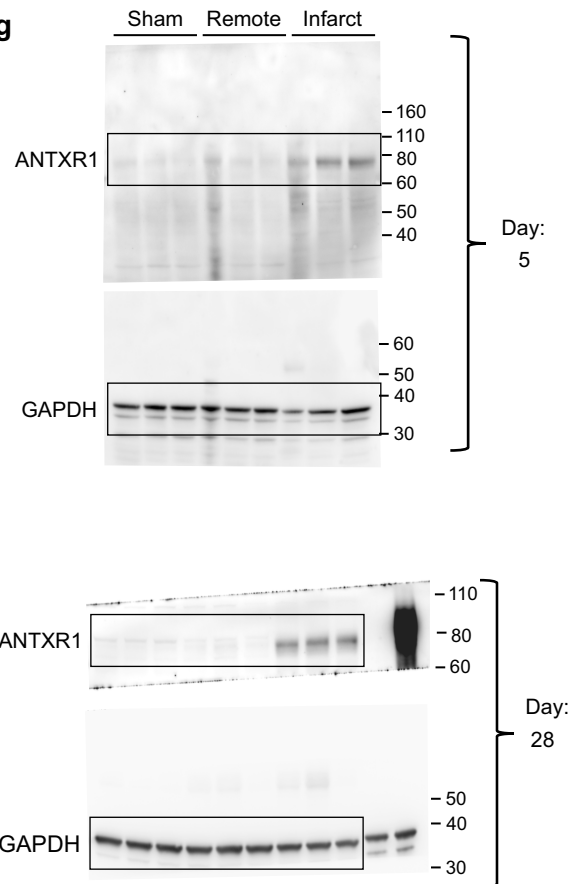

Supplement: Supplementary file 4 — Unprocessed western blots. [file 44161_2025_725_MOESM4_ESM.pdf]

Full blot images

Fig. 7a

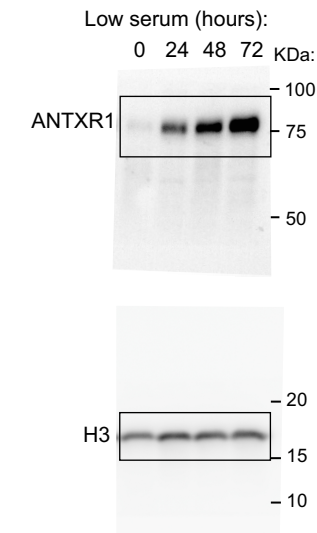

Fig. 7g

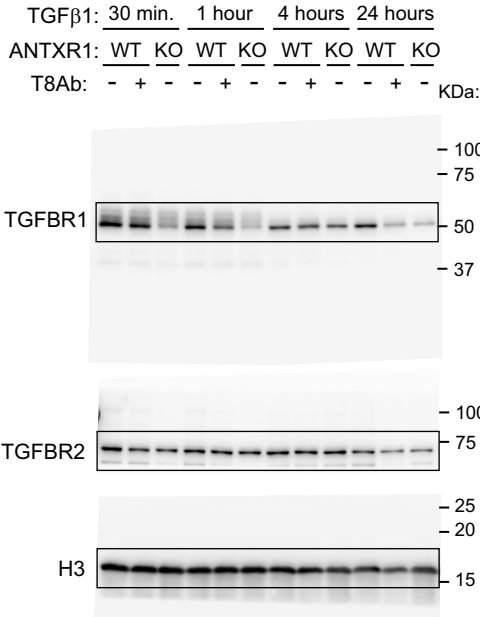

Fig. 7p

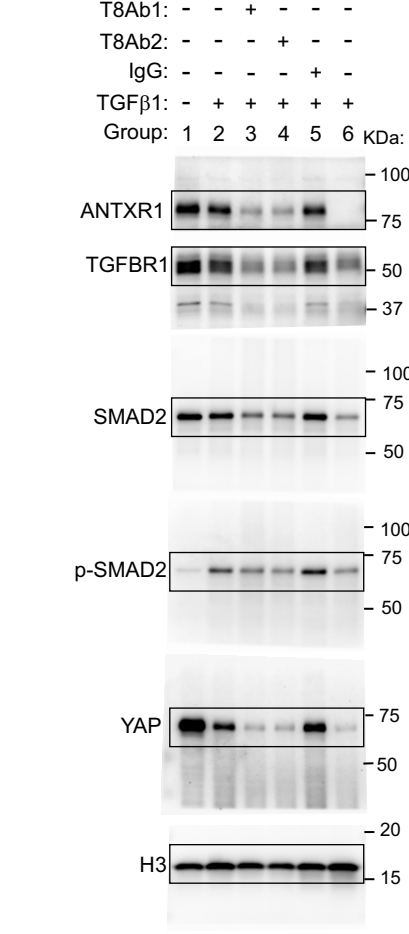

Fig. 7f

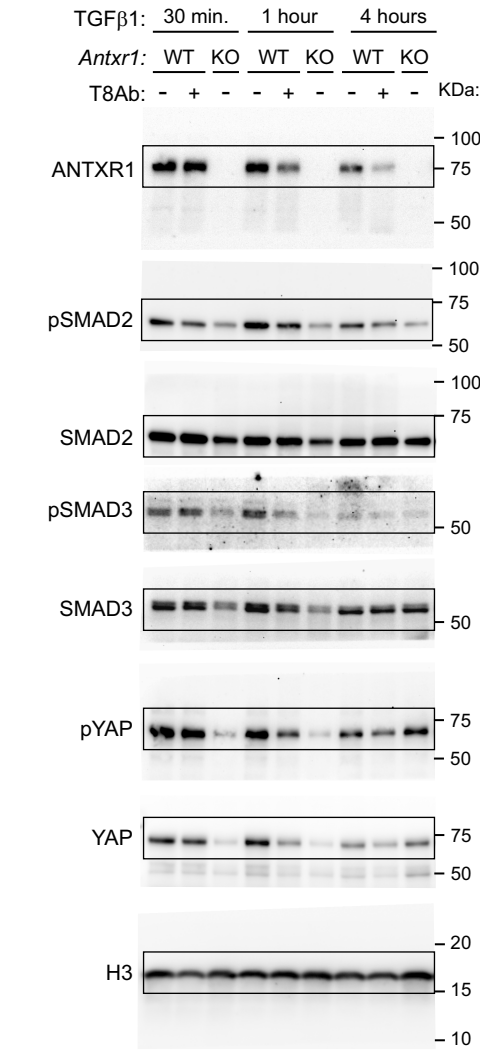

Fig. 7m

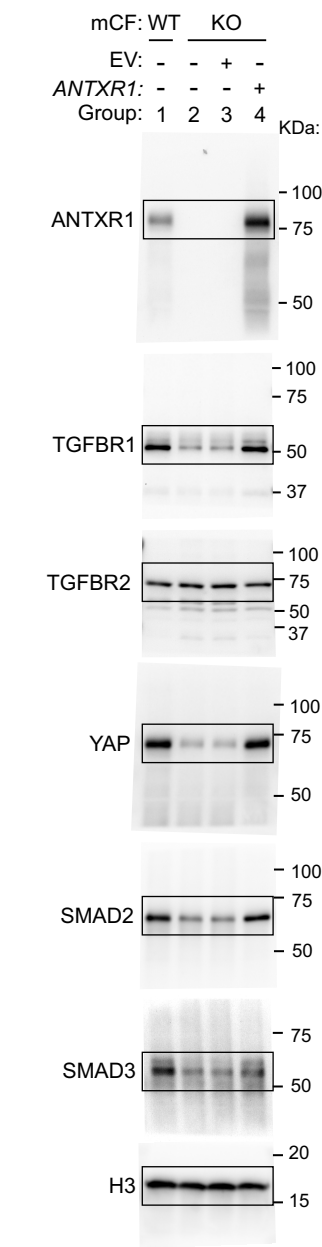

Fig. 7s

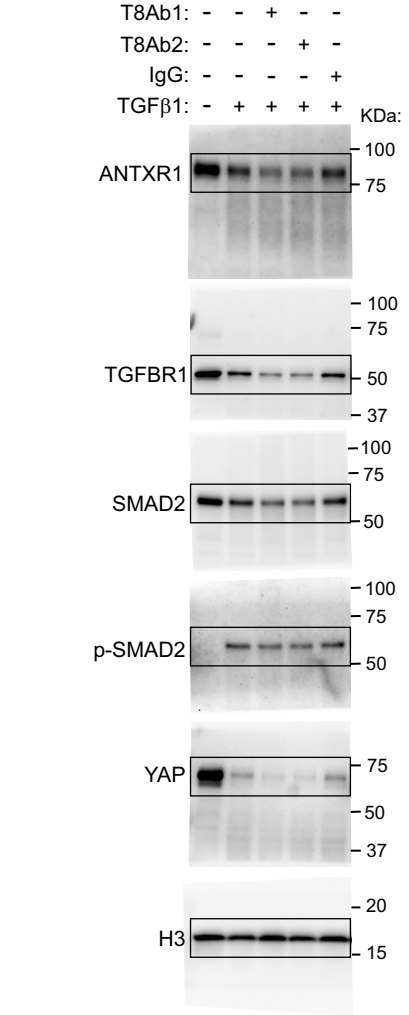

Supplement: Supplementary file 11 — Unprocessed western blots. [file 44161_2025_725_MOESM11_ESM.pdf]

## Full blot images

Extended Data Fig. 7

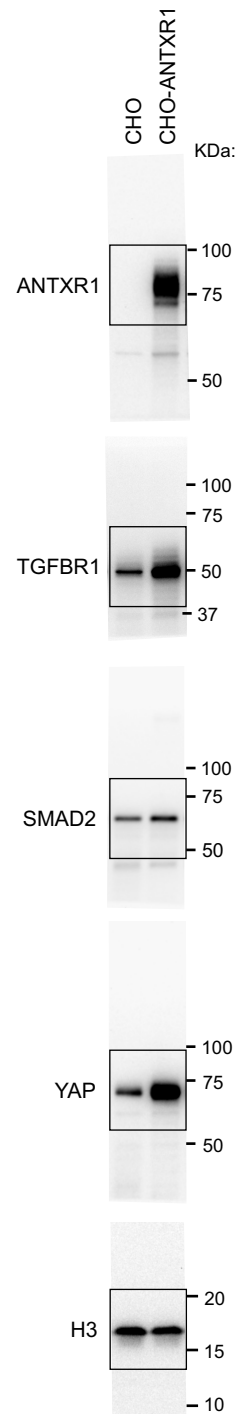

Supplement: Supplementary file 18 — Unprocessed western blots. [file 44161_2025_725_MOESM18_ESM.pdf]

Full blot images

Extended Data Fig. 8a

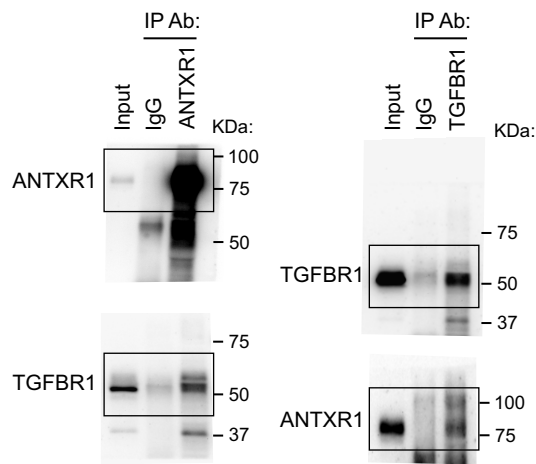

Extended Data Fig. 8e

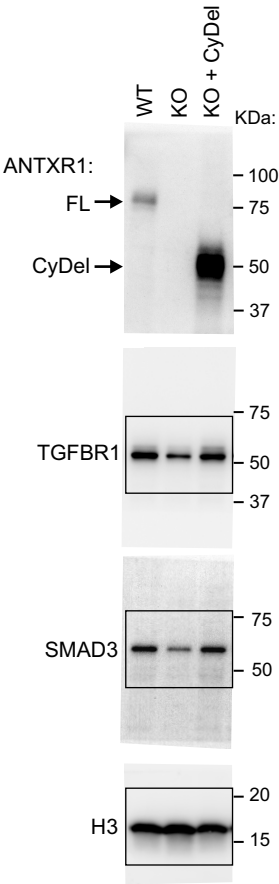

Supplement: Supplementary file 20 — Unprocessed western blots. [file 44161_2025_725_MOESM20_ESM.pdf]

## Full blot images

Extended Data Fig. 9

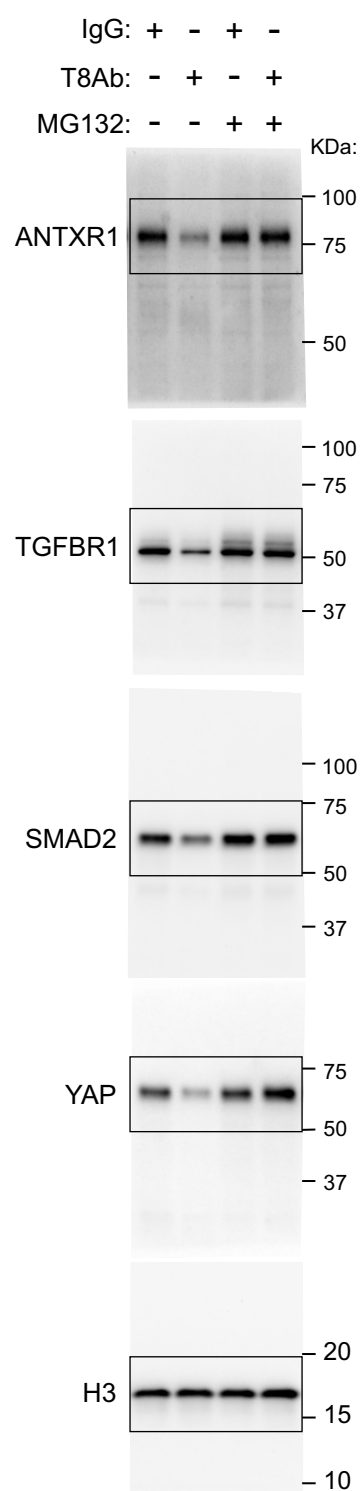

Supplement: Supplementary file 22 — Unprocessed western blots. [file 44161_2025_725_MOESM22_ESM.pdf]

Full blot images

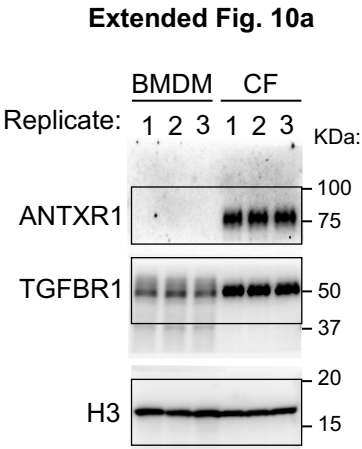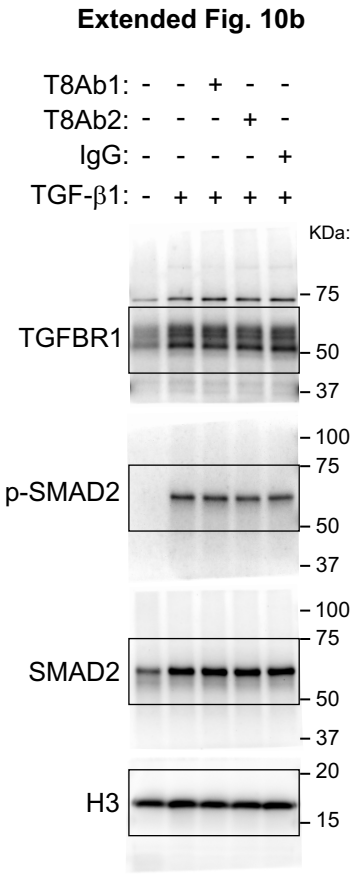

Supplement: Supplementary file 23 — Unprocessed western blots. [file 44161_2025_725_MOESM23_ESM.pdf]
